# Wearable Systems of Reconfigurable Microneedle Electrode Array for Subcutaneous Multiplexed Recording of Myoelectric and Electrochemical Signals

**DOI:** 10.1002/advs.202409075

**Published:** 2024-12-16

**Authors:** Zhengjie Liu, Chuanjie Yao, Xingyuan Xu, Xinshuo Huang, Shuang Huang, Shantao Zheng, Tao Zhang, Yan Li, Fanmao Liu, Yuxiang Wu, Jing Liu, Hui‐jiuan Chen, Xi Xie

**Affiliations:** ^1^ State Key Laboratory of Optoelectronic Materials and Technologies Guangdong Province Key Laboratory of Display Material and Technology School of Electronics and Information Technology Sun Yat‐Sen University Guangzhou 510006 China; ^2^ School of Biomedical Engineering Sun Yat‐Sen University Shenzhen 518107 China; ^3^ Department of Cardiology The First Affiliated Hospital of Jinan University Guangzhou 510630 China; ^4^ Division of Hypertension and Vascular Diseases, NHC Key Laboratory of Assisted Circulation and Vascular Diseases The First Affiliated Hospital of Sun Yat‐Sen University Guangzhou 510080 China; ^5^ Institute of Intelligent Sport and Proactive Health Department of Health and Physical Education Jianghan University Wuhan 430056 China; ^6^ Institute of Precision Medicine, The First Affiliated Hospital Sun Yat‐sen University Guangzhou 510080 China

**Keywords:** electromyogram and electrochemical signals, multi‐parameter detection, pogo pin interface, reconfigurable microneedle electrode array, transdermal recording

## Abstract

The real‐time monitoring of in vivo electrophysiological and biochemical signals provides critical insights into the activities of tissues and organs. As the activity and metabolic state of different sites in the muscle vary, multichannel detection is necessary to capture the functional state of the whole muscle, yet the access to the bio‐information in subcutaneous space remained challenging. This work reports the development of a reconfigurable microneedle electrode array integrated system designed to achieve painless and minimally invasive monitoring of subcutaneous electromyogram (EMG), oxygen species, and pH through an array of thumbtack‐shaped microneedle (TSMN) electrode. By assembling discrete TSMNs into an array, the system enables multi‐parameter detection with single microneedle resolution. The PEDOT: PSS layer is electrochemically deposited on the TSMNs, enhancing their signal‐sensing capabilities and electrochemical properties. Additionally, the design of the pogo pin interface ensures reliable signal transmission and stable device performance, while allowing flexible replacement of the TSMNs, which enhances system maintainability and longevity. Validation experiments conducted on in vivo animal models demonstrate the system's capability in real‐time monitoring of muscle fatigue and indicators related to sciatic nerve injury. These results advance the development of wearable technologies for monitoring subcutaneous physiological and biochemical information for diagnosing neuromuscular disorders.

## Graphical abstract

A reconfigurable microneedle electrode array integrated system is developed for minimally invasive and transdermal monitoring of subcutaneous electromyography, reactive oxygen species, and pH values. By assembling discrete thumbtack‐shaped microneedles into an array, the system enables multi‐parameter detection with single‐microneedle resolution. This design allows for the flexible replacement of the thumbtack‐shaped microneedles, enhancing the maintainability and lifespan of the system.

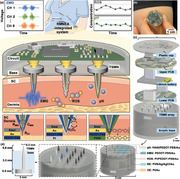

## 1 Introduction

Electrophysiological signals, including electrocardiograms, electroencephalograms, and electromyograms (EMG), in conjunction with biochemical signals, such as biomarkers and neurotransmitters in the blood, play a crucial role in understanding the intricate mechanisms of physiological activities within organisms.^[^ ^1^, ^2^, ^3^ ^]^ Among these, EMG can provide critical information about muscle excitability, functional state, and contraction properties, which are essential for diagnosing, treating, and rehabilitating of muscle diseases.^[^ ^4^, ^5^, ^6^ ^]^ However, relying solely on EMG monitoring is often insufficient to capture the full complexity of muscle function and its pathological changes.^[^ ^7^, ^8^ ^]^ During muscle contraction, changes in neurotransmitters (such as acetylcholine) and ions (such as sodium and calcium) also play a pivotal role.^[^ ^9^, ^10^ ^]^ Abnormalities in these biochemical signals may be closely associated with the occurrence and development of muscle diseases.^[^ ^11^, ^12^ ^]^ Therefore, the simultaneous detection of electrophysiological and biochemical signals in muscle tissue is of important for a comprehensive understanding of muscle function and pathological changes.^[^ ^13^ ^]^ Taking polymyositis as an example, while EMG can reveal its electrical characteristics, such as short duration, low amplitude, and polyphasic waves, these features can also appear in other muscle diseases like muscular dystrophy and metabolic myopathies.^[^ ^14^ ^]^ The combination of biochemical tests, such as the measurement of creatine kinase and lactate dehydrogenase levels in the blood, can provide crucial information about the extent of muscle damage and inflammation activity, aiding in the differentiation between various diseases.^[^ ^15^ ^]^ Given the variability in activity patterns and metabolic levels at different sites within muscle tissue, reliance on single‐channel detection methods has often been insufficient for a comprehensive and in‐depth analysis of muscle functionality.^[^ ^16^, ^17^ ^]^

Microneedle sensing technology presents new solutions for both electrophysiological recording and biochemical sensing.^[^ ^18^, ^19^ ^]^ For physiological electrical recording, microneedles can penetrate the high impedance barrier of the stratum corneum, improving the quality of the electrophysiological signals.^[^ ^20^, ^21^ ^]^ Kim et al. developed stretchable microneedle adhesive patches that painlessly penetrate the stratum corneum, ensuring durable and reliable acquisition of high‐quality electrophysiological signals under different skin conditions.^[^ ^22^ ^]^ In terms of biochemical sensing, microneedles penetrate the stratum corneum to directly detect biomarkers in the interstitial fluid (ISF), providing results that closely correlate with those in blood.^[^ ^23^, ^24^ ^]^ In addition, microneedles interact directly with biomarkers in the subcutaneous ISF, making them less susceptible to external environmental variations.^[^ ^25^, ^26^ ^]^ Furthermore, the design of microneedle arrays allows for the simultaneous identification of multiple biochemical markers, thereby promoting comprehensive health monitoring.^[^ ^27^, ^28^ ^]^ For example, Joseph Wang et al. have developed a wearable sensor device consisting of multiple sensing microneedle arrays and electronic components for continuous real‐time monitoring of glucose, alcohol, and lactate.^[^ ^29^ ^]^

However, current microneedle sensing technology is mainly used to monitor electrophysiological signals or biochemical indicators separately. The acquisition of electrophysiological signals necessitates a highly conductive modification layer, whereas electrochemical detection relies on chemically modified layers that specifically react with biochemical molecules. During the fabrication process, these layers may interfere with each other. Several challenges remain in manufacturing and system integration. Microneedles must possess sufficient hardness and strength to penetrate the stratum corneum.^[^ ^30^, ^31^ ^]^ Current methods for electrical connection between microneedles and electronic devices often involve fixing the microneedles to a printed circuit board (PCB) with silver paste or embedding microneedles in micro‐holes in the PCB.^[^ ^32^, ^33^ ^]^ These methods can become complicated and costly if the microneedles need to be replaced due to damage or performance degradation. In addition, external forces applied to the microneedles can destabilize the electrical connections, affecting the accuracy and reliability of the detection results. Although theoretically multiplexed microneedle integration is a straightforward approach, the fabrication of multi‐parameter and multi‐channels microneedle arrays has been difficult to achieve in the practical preparation. This is mainly due to the need of chemical modifications on microneedles to detect specific biochemical markers. The presence of underperforming microneedles may necessitate the re‐fabrication of the entire array, thereby complicating the stable fabrication of multi‐channel electrochemical microneedle sensors.

In this work, a reconfigurable microneedle electrode array (RMNEA) integrated system was developed for minimally invasive and transdermal monitoring of subcutaneous EMG, reactive oxygen species (ROS), and pH levels. The integrated system was based on a thumbtack‐shaped microneedle (TSMN) array design, where discrete TSMNs were assembled into an RMNEA. This configuration enabled each microneedle electrode (MNE) unit to be independently inserted or removed from the array. Such a modular design facilitated multi‐parameter detection with single microneedle resolution while mitigating crosstalk issues associated with direct chemical modification in conventional microneedle patches. The effective integration of multiplexed microneedle electrodes demonstrated good flexibility across various physiological monitoring applications, enhancing both the yield and utilization efficiency of the microneedle sensing system. The electrochemical performance and sensing characteristics of the MNEs were enhanced by electrodepositing a PEDOT: PSS modification layer. In vivo experiments using the RMNEA were conducted to detect muscle fatigue and sciatic nerve injury (SNI) in animal models. The results showed that the RMNEA‐integrated system had monitored both electrophysiological signals and biochemical indicators in subcutaneous tissue. The results demonstrated its potential for real‐time monitoring of muscle fatigue and SNI, which was of promising value in the diagnosis of neuromuscular disease and assessment of muscle function for rehabilitation.

## 2 Results and Discussion

### 2.1 Design and Fabrication of the RMNEA‐Integrated System

**Figure 1:**
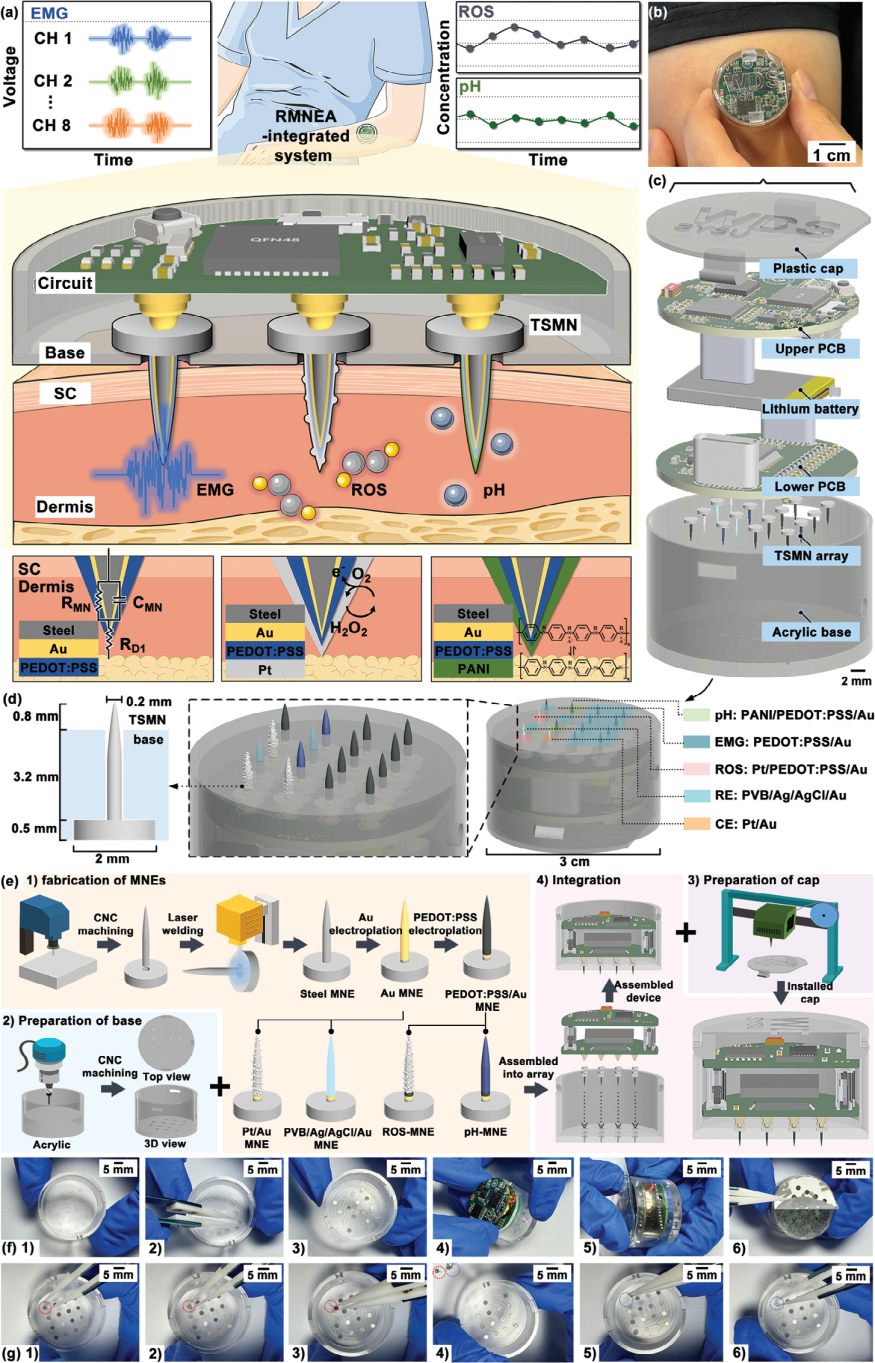
Schematic of the RMNEA‐integrated system based on TSMN arrays and detection device components. a) Schematic illustration of the RMNEA‐integrated system worn on the human arm (top) for real‐time recording of subcutaneous physiological signals. The RMNEA penetrated the skin for minimally invasive detection (middle), allowing accurate measurement of subcutaneous EMG, pH, and ROS concentrations in ISF using modified multilayer MNEs. Schematic of the sensing principles used for physiological signal detection (bottom). b) A photograph demonstrating the concept of the RMNEA‐integrated system worn on a human arm. c) Exploded view of the RMNEA‐integrated system. d) Preparation of the RMNEA using TSMN (image not to scale). The EMG sensor consisted of an eight‐channel electrode array and a reference electrode. The ROS sensor consisted of two ROS working electrodes, a counter electrode, and a reference electrode. The pH sensor consisted of two pH working electrodes and a reference electrode. After insertion into the base, the length of the TSMN tips protruding from the base was ≈800 µm. e) The fabrication process of the RMNEA‐integrated system. f) The assembly process of the RMNEA‐integrated system. g) The process of replacing TSMNs in the RMNEA‐integrated system. The red circles indicated the TSMN to be replaced, and the blue circles indicated the replacement TSMN.

An RMNEA‐integrated system was developed for continuous monitoring of EMG, pH, and ROS in subcutaneous tissues (**Figure** **1**a), which consisted of an RMNEA and a miniaturized device for wireless detection. The sensing electrodes, which were based on a thumbtack‐like structure, with PEDOT: PSS deposited on the surface of TSMN as an effective coating to enhance the electrical and electrochemical performance of the electrodes. For EMG detection, MNEs penetrated the stratum corneum of the skin, thereby reducing the impedance between the electrode and the skin. The PEDOT: PSS layer increased the electrode‐skin contact area, further reducing impedance and improving EMG signal quality. For ROS detection, applying a negative bias to the electrode promoted the decomposition of H₂O₂ molecules. During this process, H₂O₂ molecules were reduced on the MNE surface, producing oxygen and water, while releasing electrons. The electrons then entered the MNE, thereby generating a current signal. The measurement of current magnitude enabled an indirect estimation of the concentration of H₂O₂ in the solution. In acidic environments, polyaniline (PANI) protonated to form the conductive emeraldine salt, whereas, in alkaline environments, it deprotonated to the non‐conductive emeraldine base.^[^ ^34^ ^]^ PANI thus exhibited a rapid and reversible transition between its conductive states in acidic and alkaline environments, with its open circuit potential (OCP) exhibiting a linear relationship with the environmental pH. The collected physiological signals were processed and encoded by the detection device and then transmitted to a display terminal.

The RMNEA‐integrated system was composed of six distinct layers (Figure 1b,c; Figures S1–S6, Supporting Information): a plastic cap, an upper PCB, a lithium battery, a lower PCB, a TSMN array, and an acrylic base. The RMNEA was configured with 16 electrodes, arranged in a 4 × 4 grid configuration (Figure S7, Supporting Information). This array included eight channels for EMG measurement, two channels for ROS measurement, and two channels for pH measurement. The EMG sensor used a two‐electrode system with a common reference electrode. The ROS sensor used a three‐electrode system for electrochemical detection, with a common reference and counter electrode. The pH sensor used a two‐electrode system with a common reference electrode. Each MNE within the system was designed to be independently replaceable for ease of maintenance and flexibility. If an MNE became degraded or damaged, the system allowed it to be easily removed and replaced with a new MNE. The design of the RMNEA improved the cost‐effectiveness and maintenance convenience of the microneedle sensor. This design allowed users to easily replace individual MNEs as needed, thereby extending the life of the integrated system. The depth of penetration of the MNE into the skin was dependent upon the thickness of the base. By substituting bases of different thicknesses, precise control of the penetration depth could be achieved to meet different application requirements. The TSMN possessed a base diameter of 2 mm, a base thickness of 0.5 mm, a needle length of 4 mm, and a needle diameter of 200 µm. By adjusting the base thickness, the protrusion of the needle beyond the base was set at 800 µm.

Figure 1e illustrates the manufacturing process for the RMNEA and detection device components, where the detailed steps are shown as follows. First, a CNC (Computer Numerical Control) machine was used to precisely cut a steel plate to produce a circular base with a diameter of 2 mm. In the center of this circular base, a micro‐hole with a diameter of 200 µm was made using high‐precision cutting tools. The needle tip was then inserted vertically into this central hole. Laser welding was used to attach the needle tip to the base, forming the complete TSMN unit. To improve biocompatibility, the needle tips of the TSMNs were gold‐plated. The gold‐plated MNEs were then coated with Ag/AgCl ink and a polyvinyl butyral (PVB) resin layer to form the reference MNE. For the counter electrode, Pt nanoparticles were electrochemically deposited on the gold‐coated MNEs. To further optimize the performance of the sensor, the gold‐coated MNEs were modified by electro‐deposition of the PEDOT: PSS layer. This treatment reduced the interface impedance between the electrode and the skin and increased the efficiency of electron transfer between the electrode and biological molecules.^[^ ^35^, ^36^ ^]^ Subsequently, functional modifications were applied to the PEDOT: PSS/Au MNEs. Pt layers were electro‐deposited onto the PEDOT: PSS/Au MNEs to increase catalytic activity and improve sensitivity to ROS, thereby optimizing performance for ROS sensing.^[^ ^37^ ^]^ In addition, PANI was electro‐deposited on the PEDOT: PSS/Au MNEs for pH sensing. The base (Figure S8, Supporting Information) was constructed from acrylic sheets that had been processed using a CNC machine. There were 16 holes in the bottom of the base, each of which was precisely matched in diameter and depth to the diameter and thickness required for the TSMN. The plastic cap (Figure S9, Supporting Information) was manufactured using 3D printing technology to precisely fit the base. The design of the cap incorporated specific structures to interlock tightly with the base. The prefabricated TSMNs were then inserted into the corresponding positions in the base, ensuring that each TSMN was securely fixed. Next, the detection device was inserted into the base, with the pogo pins of the device aligned with the TSMN below. Finally, the cap was pressed into place with its clips firmly engaging the base, ensuring a stable electrical connection between the detection device's pogo pins and the TSMNs (Figure S10, Supporting Information). When replacing the TSMN, the TSMN to be replaced was carefully removed from the base. Subsequently, the new TSMN was inserted into the corresponding position on the base. The RMNEA‐integrated system was designed to be attached to the human arm using adhesive tape (Figure S11, Supporting Information). This work possessed advances in several aspects: i) The use of a highly repeatable machining method, specifically CNC machining, was employed to fabricate TSMN with high mechanical strength. The discrete TSMNs were further assembled into RMNEA, achieving detection resolution at the single microneedle level and avoiding crosstalk problems that could arise from direct chemical modification on microneedle patches. ii) By embedding discrete TSMNs into a base, users can freely combine and assemble TSMNs with different detection functions according to specific needs, allowing for rapid adaptation to diverse application scenarios. The assembly‐based system design also improves both the yield and the utilization of the system. iii) The minimally invasive detection of electrophysiological and biochemical parameters in subcutaneous tissue, offered the advantages of painlessness and no skin preparation. iv) This system compactly integrated the detection device directly above the TSMNs, minimizing the signal transmission path from the MNEs to the amplifier and enabling an active single microneedle electrode design.

### 2.2 Morphological Characterization and Simulation Testing of MNEs

**Figure 2:**
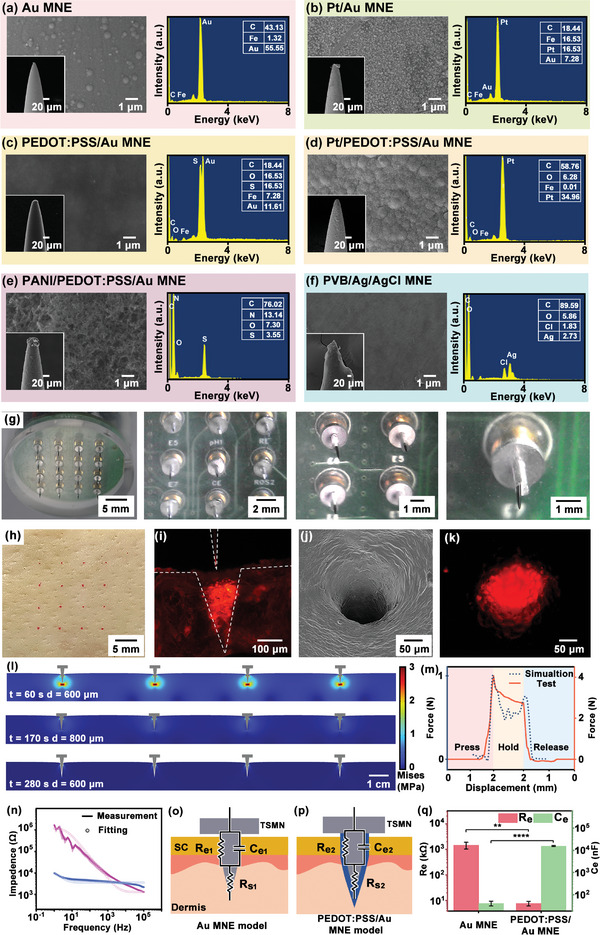
Morphological characterization, mechanical and electrical simulation, and testing of MNE. a) SEM images of Au MNEs, along with corresponding EDX spectra. b) SEM images of Pt/Au MNEs, along with corresponding EDX spectra. c) SEM images of a PEDOT: PSS/Au MNEs at multiple magnifications, along with corresponding EDX spectra. d) SEM images of Pt/PEDOT: PSS/Au MNEs (ROS‐MNEs), along with corresponding EDX spectra. e) SEM images of PANI/PEDOT: PSS/Au MNEs (pH‐MNEs), along with corresponding EDX spectra. f) SEM images of PVB/Ag/AgCl MNEs, along with corresponding EDX spectra. g) Optical images of the RMNEA‐integrated system at different magnifications, illustrating 4 × 4, 3 × 3, 2 × 2, and 1 × 1 array configurations. h) Top view of porcine skin after penetration by the RMNEA. i) Cross‐sectional optical image of porcine skin punctured by the RMNEA, with TSMN microchannels marked using Rhodamine B dye. j) SEM image of microchannels in porcine skin created by TSMN puncturing. k) Fluorescence image showing porcine skin after penetration by the TSMN. l) Stress distribution contour maps of the skin during the insertion and removal of the RMNEA. m) Comparison of force–displacement relationships between simulations and experimental measurements when inserting and removing TSMNs. n) Impedance spectra and fitting analysis of PEDOT: PSS/Au MNEs versus Au MNEs in electrode‐skin impedance measurement (*n* = 3). o) Schematic of the equivalent circuit model for the Au MNE‐skin interface. p) Schematic of the equivalent circuit model for the PEDOT: PSS/Au MNE‐skin interface. q) Comparative analysis of component values in the equivalent circuit models of the electrode‐skin interface for PEDOT: PSS/Au MNEs and Au MNEs (*n* = 3). Data were presented as mean ± SD. Significance was evaluated by a *t‐*test. **p < 0.01, ****p < 0.0001.

The structure and surface morphology of MNEs with various coating modifications were characterized using scanning electron microscopy (SEM). Following the gold electroplating process, the surface of the Au MNEs exhibited a smooth appearance (**Figure** **2**a). Elemental composition analysis via energy‐dispersive X‐ray spectroscopy (EDX) confirmed the presence of Au, C, and Fe on the MNE surface. Following the deposition of Pt onto the gold‐coated Steel MNEs, the surface exhibited a uniform distribution of Pt nanoparticles (Figure 2b). The results of the EDX analysis indicated the presence of Pt, in addition to the previously identified elements, indicating that Pt nanoparticles were uniformly deposited on the MNE surface. When the Au MNEs were further modified by the electro‐deposition of PEDOT: PSS, the MNE surface became smooth and uniform (Figure 2c). EDX analysis confirmed the presence of S, C, and O elements, which confirmed the successful deposition of PEDOT: PSS on the Au MNEs and the formation of a homogeneous film. Further deposition of Pt nanoparticles on the PEDOT: PSS/Au MNEs by SEM showed that the Pt nanoparticles were successfully deposited on the PEDOT: PSS surface (Figure 2d). Following the electrodeposition of Pt, EDX analysis showed distinct Pt peaks with higher intensity, indicating a substantial loading of Pt elements on the MNE surface. The electropolymerization of PANI on the PEDOT: PSS/Au MNEs resulted in the formation of a network of interwoven PANI nanofibers, which collectively constituted a porous layered structure, as observed in SEM images (Figure 2e). EDX analysis identified C, N, S, and O as the predominant elements, further confirming the presence of PANI on the MNE surface. The morphology of the Ag/AgCl MNE coated with PVB mixed solution is shown in Figure 2f. The PVB coating was observed to be fully adherent to the surface of the Ag/AgCl MNEs, forming a continuous and uniform film. The consistency and uniformity of the PVB layer ensured effective ion transfer across the MNE surface. EDX analysis showed a higher proportion of C and O elements, together with the detection of Ag and Cl, confirming that PVB successfully encapsulated the Ag/AgCl MNE surface (Figure S12, Supporting Information). The discrete MNEs were embedded into the base and subsequently assembled with the detection device and the cap, thereby forming an RMNEA‐integrated system. As illustrated in Figure 2g, the TSMNs were positioned in a 4 × 4 array, accurately aligning with the pre‐defined apertures in the base. The enlarged view indicated that each TSMN was spaced 5 mm apart with no electrical connections between adjacent TSMNs, thus preventing crosstalk. Each TSMN was aligned with the corresponding pogo pins on the circuit, thus ensuring that each TSMN possessed an independent signal detection capability. The length of each TSMN extending from the base was exactly 800 µm and maintained a perpendicular orientation to the base. Each TSMN tip possessed a sharp conical shape with the exposed electrode surface remaining intact (Figure S13, Supporting Information). When the device was inserted into the skin, the needle tips penetrated the skin tissue, enabling the monitoring of subcutaneous physiological signals. The part of the base in contact with the outer stratum corneum of the skin isolated surface interference, ensuring that signals from the skin surface did not affect the detection of the TSMNs or the sensing circuitry.

In order to validate the skin penetration capability of the RMNEA, a porcine skin penetration test was performed. First, the TSMN tips were stained with the red fluorescent dye Rhodamine B. The RMNEA was then positioned perpendicular to the surface of fresh porcine skin and a downward force was applied to press the RMNEA against the skin. After maintaining the applied force for 3 min, the RMNEA was removed. As shown in Figure 2h, red spots were observed on the surface of the skin, arranged similarly to the RMNEA, and spaced 5 mm apart. To further examine the penetration depth, the porcine skin was sliced into sections ≈1 mm thick along the TSMN penetration sites. Fluorescence microscopy was employed to image the regions of the skin that had been penetrated by the TSMNs. In Figure 2i, the fluorescent regions corresponding to the TSMN insertion sites were ≈200 µm in diameter, closely matching the diameter of the TSMN tips. Post‐penetration examination through SEM (Figure 2j) and fluorescence imaging (Figure 2k) revealed the formation of circular pores with a diameter of ≈200 µm on the skin surface. The transdermal penetration capability of the RMNEA was preliminarily validated through the porcine skin penetration test. To further quantify the mechanical interactions between the microneedles and the skin, numerical simulations were performed to model the process of RMNEA insertion into the skin. Given the 4 × 4 array configuration of the system, the analysis was simplified to consider the interaction of four TSMNs in a cross‐sectional plane. Each microneedle had a tip radius of 10 µm, a length of 800 µm, and was uniformly spaced 5 mm apart above the skin surface. A downward displacement load (at a rate of 10 µm s^−1^) was applied to each TSMN, simulating insertion into the skin. Upon reaching an insertion depth of 800 µm, the TSMNs were maintained in a stationary position within the skin for a period of 180 s. Subsequently, an upward displacement load was applied to simulate the removal process. The model was meshed in order to ensure an adequate density in the critical contact regions between the TSMNs and the skin tissue.^[^ ^38^ ^]^ The von Mises stress distribution in the skin was obtained with the solid mechanics module (Figure 2l; Figure S14, Supporting Information). During insertion (Figure S15, Supporting Information), the stress was primarily concentrated directly below the needle tip, reaching up to 3 MPa, with relatively lower stress on the sides of the needle tip. As the TSMNs transitioned to the stationary phase (Figure S16, Supporting Information), the stress in the skin gradually decreased over time, attributed to the gradual relaxation of the skin tissue. During the removal phase (Figure S17, Supporting Information), the primary forces became frictional forces on the sides of the needle tip. As the TSMNs were gradually withdrawn, the stress on the skin decreased progressively. To verify the accuracy of the simulation model and to better understand the mechanical behavior between the microneedles and the skin, mechanical tests were performed using porcine skin as a substitute for human skin. During the tests, the RMNEA was mounted on a force gauge probe and an electric moving stage precisely controlled the vertical insertion into the porcine skin (Figure S18, Supporting Information). Figure 2m illustrates the mechanical variations throughout the insertion process. During the insertion phase, the reaction force gradually increased as the RMNEA penetrated the porcine skin. When the reaction force reached 1.5 N, the curve showed noticeable fluctuations, indicating that the RMNEA had successfully penetrated the skin. Following complete penetration, the reaction force exhibited a linear increase with a slope of ≈20 N mm^−1^, reaching a peak of 4.1 N after a 2 mm descent. During the stationary phase, the reaction force decreased from the peak of 4.1 to 2.8 N. This decrease was due to the skin tissue gradually adapting to the presence of the RMNEA and possibly beginning to regain its original elasticity, leading to a reduction in the adhesive forces between the RMNEA and the skin. In the final stage, when the RMNEA was separated from the porcine skin, the reaction force gradually decreased to 0 N. The mechanical tests showed that the variation of microneedle force with displacement was similar to the trends observed in the simulation tests. The RMNEA was repeatedly inserted and removed 10 times on porcine skin, resulting in minimal morphological changes to the TSMN (Figure S19, Supporting Information), with no damage observed.

In addition to investigating the mechanical properties of the microneedle‐skin interaction, the electrical properties were also investigated. This study aimed to investigate the impact of different MNE surface modifications on the performance of microneedle sensors, with a particular focus on comparing PEDOT: PSS/Au MNEs with Au MNEs. Initially, the contact impedances between MNEs and the skin were measured. As shown in Figure 2n, the impedance of both MNE types decreased with increasing frequency. However, the impedance of the PEDOT: PSS/Au MNEs decreased more rapidly than that of the Au MNEs, which showed a more gradual decrease. Specifically, in the frequency range from 0 to 5000 Hz, the impedance of the PEDOT: PSS/Au MNEs was lower than that of the Au MNEs. At a frequency of 1 kHz, the average impedance of the PEDOT: PSS/Au MNEs was 3.89 ± 0.56 kΩ, while the average impedance of the Au MNEs was 8.24 ± 1.58 kΩ. By fitting the parameters of the model to match the calculated impedance as closely as possible to the actual measured impedance, the specific parameters of the circuit models were obtained, as shown in Figure 2o,p. The results showed that the PEDOT: PSS coating reduced the contact impedance between the Au MNEs and the skin. The improved conductivity of the PEDOT: PSS coating facilitated the transfer of charge between the electrode and the skin, resulting in a lower contact resistance (Re) for the PEDOT: PSS/Au MNEs compared to the MNEs (Figure 2q).^[^ ^39^ ^]^ In addition, the PEDOT: PSS coating improved the contact between the electrode and the skin and increased the effective area of the double layer, thereby increasing the double layer capacitance (Ce). Consequently, the double‐layer capacitance for the PEDOT: PSS/Au MNEs was greater than that for the Au MNEs. These results highlighted the important role of the PEDOT: PSS coating in optimizing the electrical interface between the MNEs and the skin.

### 2.3 In Vitro Performance of MNEs

**Figure 3:**
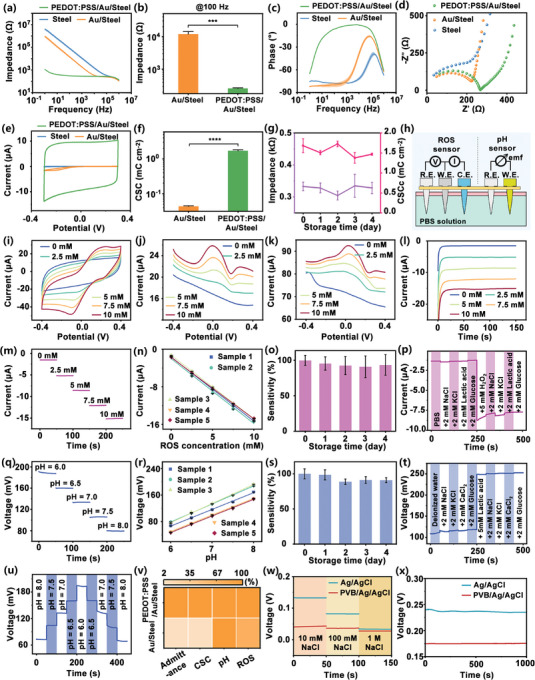
Electrochemical characterization of MNE sensors in vitro. a) Impedance characteristics of Steel, Au/Steel, and PEDOT: PSS/Au/Steel MNEs measured across various frequencies (*n* = 3). b) Comparison of impedance values for Au and PEDOT: PSS MNEs at a fixed frequency of 100 Hz (*n* = 3). c) Phase characteristics of Steel, Au/Steel, and PEDOT: PSS/Au/Steel MNEs across different frequencies (*n* = 3). d) Nyquist plots illustrating the impedance spectra of Steel, Au/Steel, and PEDOT: PSS/Au/Steel MNEs. e) CV of Steel, Au/Steel, and PEDOT: PSS/Au/Steel MNEs in PBS solution, recorded over a potential range of −0.3 to 0.3 V at a scan rate of 100 mV s^−1^. f) Comparison of the CSCc between Au and PEDOT: PSS MNEs (*n* = 3). g) Time‐course monitoring of specific impedance (at 100 Hz) and CSCc of PEDOT: PSS/Au/Steel MNEs over a period of 5 days (*n* = 3). h) Schematic diagrams showing the connections for the three‐electrode system used for ROS measurements and the two‐electrode system used for pH measurements. i) CV response of the ROS‐MNE in H₂O₂ solutions with concentrations ranging from 0 to 10 mm. j) DPV response of the ROS‐MNE to various concentrations of H₂O₂. k) SWV response of the ROS‐MNE to different concentrations of H₂O₂. l) *i–t* response of the ROS‐MNE in solutions with varying concentrations of H₂O₂. m) Linear response of the ROS‐MNE to H₂O₂ concentrations ranging from 0 to 10 mmol L^−1^. n) Consistency in the response of five different ROS‐MNEs tested in parallel for detecting various concentrations of H₂O₂. o) Statistical analysis of the stability of the ROS‐MNE's linear response to different H₂O₂ concentrations over a period of five days, represented as normalized values (*n* = 3). p) Interference testing of the ROS‐MNE in PBS solution, evaluating the impact of adding 2 mm NaCl, 2 mm KCl, 2 mm lactic acid, or 2 mm glucose. q) Performance of the pH‐MNE across a pH range from 5.0 to 8.0. r) Consistency in the response of five different pH‐MNEs tested in parallel for detecting solutions with various pH levels. s) Statistical analysis of the stability of the pH‐MNE's linear response to different pH levels over five days, represented as normalized values (*n* = 3). t) Interference testing of the pH‐MNE in deionized water, with the addition of potential interferents including 2 mm NaCl, 2 mm KCl, 2 mm CaCl₂, or 2 mm glucose. u) Evaluation of the reversibility of the pH‐MNE within a pH range from 5.0 to 8.0. v) Heatmap summarizing the electrochemical performance metrics of the MNEs, including admittance, CSCc, pH detection sensitivity, and ROS detection sensitivity. w) OCP response of Ag/AgCl MNEs with and without PVB coating in NaCl solutions of varying concentrations. x) Long‐term OCP response of Ag/AgCl MNEs with and without PVB coating in deionized water. Data were presented as mean ± SD. Significance was evaluated by *t*‐test. ***p < 0.001, ****p < 0.0001.

The performance of the as‐prepared MNEs was evaluated by in vitro testing. By comparing with the impedance characteristics of Steel, Au/Steel, and PEDOT: PSS/Au/Steel MNEs in PBS solution (**Figure** **3**a), it was found that the PEDOT: PSS/Au/Steel MNEs exhibited the lowest impedance over the frequency range of 1 Hz to 100 kHz, while the Steel MNEs exhibited the highest impedance. This reduction in impedance can be attributed to the role of PEDOT: PSS layer, which increased the charge transfer rates. As shown in Figure 3b, at 100 Hz (the central frequency for EMG), the impedance of the Au/Steel MNEs decreased from 14.53 ± 2.81 to 0.31 ± 0.28 kΩ for the PEDOT: PSS/Au/Steel MNEs. In addition, the phase angle analysis in Figure 3c showed that the PEDOT: PSS/Au/Steel MNEs possessed a lower phase delay compared to the Steel and Au/Steel MNEs, indicating a faster charge transfer process on the PEDOT: PSS/Au/Steel MNE surface. The Nyquist plot of the impedance spectra (Figure 3d) further confirmed that the PEDOT: PSS/Au/Steel MNEs possessed strong diffusion properties. Cyclic voltammetry (CV) measurements were performed on Steel, Au/Steel, and PEDOT: PSS/Au/Steel MNEs in PBS solution, with the results shown in Figure 3e. The comparison of the redox peak current densities in the CV curves showed that the PEDOT: PSS/Au/Steel MNEs had a higher current density, which was attributed to the larger electrochemically active surface area provided by the PEDOT: PSS coating. Furthermore, in the multi‐cycle CV measurements, the PEDOT: PSS/Au/Steel MNEs showed high stability with no significant loss of current or reduction of the oxidation peak over time (Figure S20, Supporting Information). These observations demonstrated the superior electrochemical performance of the PEDOT: PSS/Au/Steel MNEs, rendering them suitable for applications requiring efficient charge transfer and stability. Further calculations (Figure 3f) were carried out to evaluate the cathodic storage capacity (CSCc) of the MNEs. For the PEDOT: PSS/Au/Steel MNEs, the deposition of PEDOT: PSS increased the active surface area of the MNEs, resulting in a higher CSCc compared to the MNEs without PEDOT: PSS coating. The above results indicated that the PEDOT: PSS/Au/Steel MNEs exhibited superior electrical performance and reduced interface impedance. These characteristics were crucial for electrophysiological signal detection because they reduced the contact resistance between the electrode and the skin. This reduction improved the performance at the electrode–skin interface and increased the accuracy of electrophysiological signal detection. The introduction of the PEDOT: PSS coating also increased the MNE's surface area, providing more sites for electrochemical reactions. More reaction sites allowed greater exchange and transfer of charges at the electrode surface, thereby increasing the efficiency and rate of electrochemical reactions.^[^ ^40^ ^]^ The utilization of the PEDOT: PSS layer helped to improve the accuracy and reliability of electrochemical and electrophysiological measurements (Figure S21, Supporting Information).^[^ ^41^ ^]^ In order to evaluate the long‐term electrochemical stability (Figure 3g) of the PEDOT: PSS/Au/Steel MNEs, their impedance, and CSCc were monitored for 5 consecutive days. The results showed that the PEDOT: PSS/Au/Steel MNEs maintained high stability, with impedance variations of less than 3.8% and CSCc changes of less than 9.5%. This stability underlined their robustness for prolonged use in bioelectronic applications.

Further modifications were made to the PEDOT: PSS/Au/Steel MNEs through electrodeposition, allowing the detection of ROS and pH signals. As illustrated in Figure 3h, a three‐electrode electrochemical system was used to test the ROS‐MNEs, whereas a two‐electrode electrochemical system was used to test the pH‐MNEs. To evaluate the performance of the ROS‐MNE, hydrogen peroxide (H₂O₂) was selected as the test analyte. In PBS solution, varying concentrations of H₂O₂ were tested using CV, differential pulse voltammetry (DPV), and square wave voltammetry (SWV). In the CV tests (Figure 3i), distinct redox peaks were observed at a potential of ±0.1 V. Further analysis showed that the redox peak currents increased linearly with increasing concentrations of H₂O₂. For the DPV tests (Figure 3j), the peak currents on the DPV curves also increased progressively with higher concentrations of H₂O₂. Similarly, the SWV tests (Figure 3k) showed an increase in the peak currents of the square wave curves with increasing concentrations of H₂O₂. A comprehensive analysis of the CV, DPV, and SWV electrochemical methods indicated that the sensor had good electrochemical response characteristics across a range of H₂O₂ concentrations, with a wide linear range and high sensitivity. Based on the redox peak information obtained from the CV tests, a bias voltage of −0.1 V was applied to record the amplitude of the current signal, thereby enabling dynamic detection of H₂O₂ in solution. As shown in Figure 3l, the amperometric response of the fabricated Pt/PEDOT: PSS/Au MNEs was tested over a concentration gradient from 0 to 10 mm. The steady‐state current values were used to quantitatively analyze the concentration of H₂O₂, revealing a linear increase in steady‐state current with increasing H₂O₂ concentration (Figure 3m). This linear relationship between the current response and H₂O₂ concentration indicated a direct correlation for accurate detection. To assess reproducibility (Figure 3n), the amperometric responses of five ROS‐MNE samples from different batches were compared. The response of each sample increased with higher H₂O₂ concentrations, demonstrating good consistency between different sensors. Statistical analysis (Figure S22, Supporting Information) showed that the average sensitivity of PEDOT: PSS/Au/Steel MNEs was −1.37 ± 0.02 µA mm ^−1^ (*R* ^2^ ≥ 0.99), which was higher than that of Au/Steel MNEs of −1.01 ± 0.03 µA mm ^−1^ (*R* ^2^ ≥ 0.99). In addition, the long‐term stability of the ROS‐MNEs was investigated to explore their storage stability. The sensitivity changes were calibrated by setting the sensitivity of the initial day as 100% and measuring the subsequent daily sensitivity variations. The ROS‐MNEs demonstrated relatively stable current levels over five days, with values of 95.54 ± 9.39%, 92.56 ± 12.74%, 91.02 ± 15.22%, and 93.55 ± 14.46% (Figure 3o). As the ISF in the human body contains various metabolites and other biological molecules, the ROS‐MNEs may be subject to interference from these substances, potentially affecting the accuracy of H₂O₂ detection. To assess the selectivity of the ROS‐MNEs, NaCl, KCl, lactate, and glucose were sequentially added to the test solution (Figure 3p). The results (Figure S22, Supporting Information) demonstrated that the ROS‐MNEs exhibited a significant current response only when H₂O₂ was added, while the presence of interfering substances produced only small current responses (<3.83%). This confirmed that the ROS‐MNEs could selectively detect H₂O₂ without being affected by other interfering substances. The pH‐MNEs were fabricated by electrodepositing PANI on the PEDOT: PSS layer (Figure S23, Supporting Information). The pH‐MNEs were then tested in different pH buffer solutions, with the potential signal showing a linear variation over the pH range of 5.0 to 8.0 (Figure 3q). The response of five pH‐MNEs was compared to assess reproducibility (Figure 3r). The results showed that the pH‐MNEs exhibited similar trends in response to changes in pH and had comparable sensitivities. Statistical analysis (Figure S24, Supporting Information) also showed that the average sensitivity of PEDOT: PSS/Au/Steel MNEs was closer to that of Au/Steel MNEs, with a sensitivity of 50.32 mV pH^−1^ (*R* ^2^ ≥ 0.99) for PEDOT: PSS/Au/Steel MNEs and 53.94 mV pH^−1^ (*R* ^2^ ≥ 0.99) for Au/Steel MNEs. To investigate the stability of the pH‐MNEs over time, the changes in sensitivity were measured over a period of 5 days (Figure 3s). Using the sensitivity of the first day as a baseline (set to 100%), the measured sensitivities were 98.57 ± 7.14%, 88.81 ± 3.82%, 91.17 ± 4.74% and 91.02 ± 3.46%, indicating relatively stable performance of the pH‐MNEs over 5 days. To evaluate the selectivity of the pH‐MNEs, deionized water was added sequentially with NaCl, KCl, CaCl₂, and glucose (Figure 3t). The results (Figure S24, Supporting Information) demonstrated that the pH‐MNEs exhibited a significant voltage response only when lactic acid was added, while the presence of interfering substances resulted in minimal voltage responses (<0.76%). This confirmed that the pH‐MNEs were unaffected by other interfering substances and could selectively detect pH changes. To investigate the reversibility of the sensors (Figure 3u), a reversibility evaluation was performed in pH buffer solutions ranging from pH 5.0 to 8.0. The results showed rapid and reversible transitions between acidic and basic conditions, indicating good reversibility of the pH‐MNEs. The findings suggested that both the ROS‐MNEs and pH‐MNEs were capable of detecting their respective analytes with good sensitivity, exhibiting a linear response signal corresponding to the changes in analyte concentration. To comprehensively investigate the electrochemical properties of MNEs modified with PEDOT: PSS, a series of comparative experiments were conducted against unmodified Au/Steel MNEs and PEDOT: PSS/Au/Steel MNEs. As illustrated in Figure 3v, a series of standardized tests were performed to evaluate the impedance characteristics, CSCc, ROS response, and pH sensing capabilities of both types of MNEs. The results demonstrated that compared to unmodified Au/Steel MNEs, the PEDOT: PSS/Au/Steel MNEs exhibited enhancements in impedance characteristics, CSCc, and ROS response. In particular, the incorporation of PEDOT: PSS reduced the electrode impedance, thereby improving the charge transfer efficiency. Furthermore, the PEDOT: PSS enhancement increased the effective surface area of the electrodes, which in turn enhanced their response to ROS. However, in terms of pH sensing, the PEDOT: PSS/Au/Steel MNEs showed comparable performance to the unmodified Au/Steel MNEs. This phenomenon can be attributed to the pH sensing mechanism, which mainly depends on the intrinsic properties of the metal surface of the electrodes.

In addition, the accuracy and reliability of the reference electrode had a significant impact on the overall performance of the electrochemical system. Therefore, the performance of the custom reference MNEs was rigorously validated. Comparative tests were carried out on Ag/AgCl MNEs with and without a PVB coating immersed in NaCl solutions. As shown in Figure 3w, both types of MNEs showed a stepwise increase in potential in response to increasing NaCl concentrations, indicating their sensitivity to changes in ion concentration within the solution. However, the Ag/AgCl MNEs coated with PVB showed better potential stability than the uncoated Ag/AgCl MNEs. In particular, the uncoated Ag/AgCl MNEs exhibited considerable potential fluctuations in response to varying concentrations of NaCl, whereas the PVB‐coated MNEs maintained a more stable potential profile. Subsequently, the long‐term stability of both types of MNEs was evaluated in deionized water over a period of 1000 s (Figure 3x). The PVB‐coated Ag/AgCl MNEs showed improved stability, maintaining a consistently lower background current signal throughout the test period compared to their uncoated counterparts. These findings indicated that the incorporation of a PVB coating had the potential to enhance the performance of the reference MNE, rendering it more suitable for applications requiring prolonged stability.

### 2.4 Design and Development of the Detection Device

**Figure 4:**
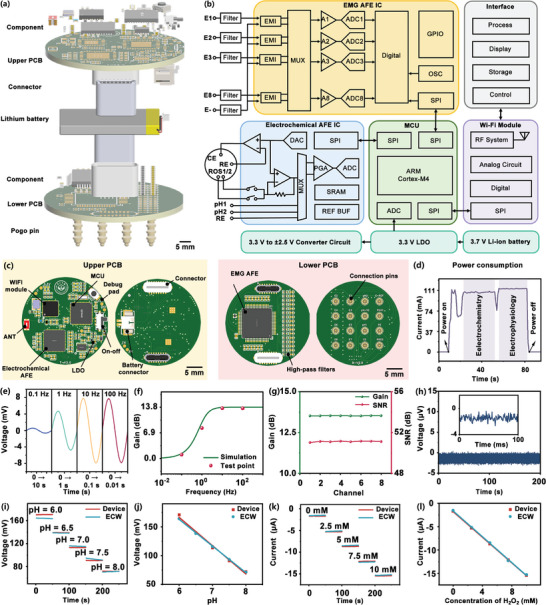
Detection device design and functional validation. a) Exploded view illustrating the components of the detection device. b) Simplified functional block diagram. The diagram outlined the key components and their interactions within the detection device, recreated based on the data sheets for AD5941 and ADS1299 to detail the signal processing and control pathways. c) Front and rear views of the two PCBs of the detection device. d) Power consumption of the detection device. e) Signal waveform acquired by the detection device from a signal generator (a sine wave with an amplitude of 8 mV). f) Frequency response simulation of the EMG filtering circuit. g) Gain and SNR across eight channels for the same test signal. h) Baseline noise detection over 200 s. The inset provided a closer look at a 100 ms segment. i) OCP of the pH‐MNE measured using the detection device and ECW. j) Linearity comparison of the detection device and ECW for pH measurements. k) Amperometric response of the ROS‐MNE measured by the detection device and ECW. l) Linearity comparison of the detection device and ECW for ROS concentration measurements.

A detection device was developed that integrated EMG and electrochemical AFE chips, a microcontroller unit (MCU), a power management module, a Wi‐Fi communication module, and a lithium battery. The utilization of AFE chips has enabled a reduction in the overall size of the detection device, resulting in a compact size and weight of only 15.56 g (Figures S25,S26, Supporting Information). As shown in **Figure** **4**a, the detection device was constructed in three layers: the top and bottom layers each had a PCB, while the middle layer had a lithium battery. The lower PCB was connected to the TSMNs via pogo pins. The simplified block diagram in Figure 4b outlined the main circuitry and electronic components of the detection device, enabling the detection of multiple physiological signals, including eight‐channel EMG, two‐channel pH potential, and two‐channel ROS. The lithium‐ion battery provided a voltage of 3.7 V, which was converted by the power module to meet the voltage requirements of the other modules. The MCU communicated with the software via the Wi‐Fi communication module and controlled the two AFE chips via the serial peripheral interface. Upon receipt of measurement commands transmitted wirelessly from the software, the MCU wrote these commands to the AFE chips. Subsequently, the MCU decoded the data and transmitted the measurement results from the AFE chips back to the software via the Wi‐Fi communication module. For the eight‐channel EMG acquisition, baseline drift problems encountered during the measurement process were mitigated using an RC high‐pass filter with a cut‐off frequency of 1 Hz. The EMG AFE chip (ADS1299) then amplified the potential signals detected by the MNEs using a programmable gain amplifier. These amplified signals were then subjected to analogue‐to‐digital conversion (ADC) to ensure low‐noise signal processing. In order to achieve the design strategy for active microneedle electrodes, the EMG AFE chip was integrated above the RMNEA, placing the amplifier in close proximity to MNEs. This arrangement aimed to minimize noise contamination of the EMG signals during transmission through long wires, thereby increasing the stability and reliability of the EMG signals in complex environments. The electrochemical AFE chip (AD5941) was used for the two‐channel pH potential and two‐channel ROS detection. This chip integrated various configurable modules required for electrochemical measurements and features multiplexers that allow the user to select the input channels for measurement, including multiple external current or voltage inputs. In the configuration of the pH potential detection setup, the AD5941 was set up in a two‐electrode system. In this configuration, the two pH‐MNEs were multiplexed to the positive input of the ADC, while the combined reference MNE was connected to the negative input of the ADC. In order to facilitate the detection of ROS, the AD5941 was configured in a three‐electrode system. In this configuration, the two ROS‐MNEs were multiplexed to a trans‐impedance amplifier, while the counter and reference MNEs were connected to the potentiostatic circuit. A low‐power Wi‐Fi communication module was employed to wirelessly transmit data to the computer, where custom software was used for waveform display and data storage (Figures S27–S30, Supporting Information). To prevent interference between the detection of electrochemical signals and electrophysiological signals, a time‐division acquisition method was used during signal acquisition. By acquiring EMG signals and electrochemical signals in separate time windows, mutual interference was reduced, ensuring signal accuracy during simultaneous multi‐parameter monitoring. Users could send commands to the acquisition circuit through the software to control acquisition parameters and switch between acquisition channels.

The power consumption of the detection device was evaluated by measuring the average power draw under a 3.7 V power supply. Figure 4c and Figure S31 (Supporting Information) presented the layout and photographs of the PCBs. The schematics for each individual PCB were presented in Figures S32 and S33 (Supporting Information). Figure 4d illustrates the power consumption profile of the device over a 100‐second duration while performing different operations. Upon startup, the device completed its initialization and entered an idle mode, exhibiting a peak current of 63 mA. Subsequently, a peak current of 106 mA was observed during electrochemical detection. During EMG detection, the peak current increased further, reaching 114 mA. The detection device was able to maintain an idle state for 140 min (Figure S34, Supporting Information). The temperature increase during device operation was assessed using infrared thermography (Figure S35, Supporting Information). To verify the functionality of the detection device, performance tests were conducted. An 8‐channel input was subjected to a sinusoidal wave with an amplitude of 8 mV, and the frequency was varied in order to characterize the gain of EMG detection across different frequencies. As illustrated in Figure 4e, the recorded signals were consistent with the simulated frequency response depicted in Figure 4f. When the sinusoidal frequency was set to 10 Hz, the voltage gain and signal‐to‐noise ratio (SNR) of all eight channels were evaluated, as illustrated in Figure 4g. The results showed a uniform gain of 51 dB across all channels, demonstrating good channel‐to‐channel consistency. The baseline noise of the system (Figure 4h), recorded with the working electrode shorted to the reference electrode over a 200‐second period, was found to be less than 3 µV. The detection results for H₂O₂ concentration and pH values were consistent with those obtained using a commercial electrochemical workstation (ECW). Both methods produced similar standard curves based on signal concentration, as shown in Figure 4i–l and Figure S36 (Supporting Information).

### 2.5 In Vivo Detection of Muscle Fatigue by RMNEA‐Integrated System

**Figure 5:**
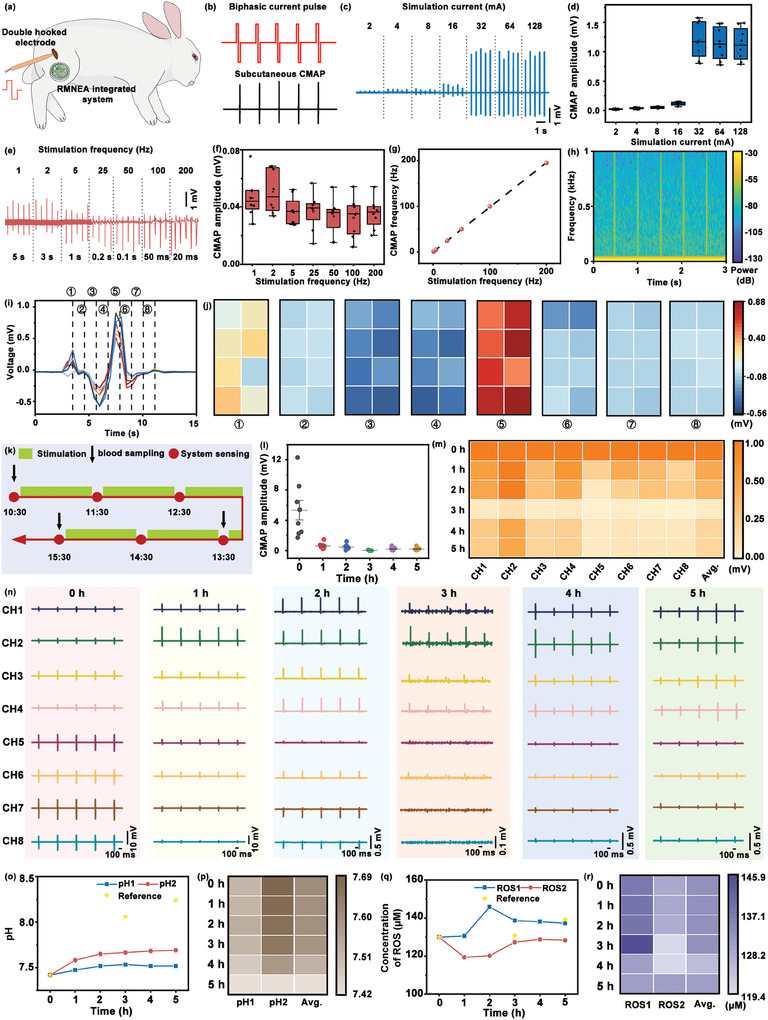
In vivo detection of muscle fatigue induced by prolonged stimulation of the sciatic nerve. a) Schematic illustration of the in vivo experimental setup. The sciatic nerve was carefully exposed and clamped with double‐hooked electrodes. Electrical stimulation was applied to evoke CMAPs. The RMNEA‐integrated system was positioned on the leg muscle to record the evoked signals. b) CMAP responses recorded during a series of 2 mA stimulation pulse trains. c) CMAPs recorded from the sciatic nerve at different stimulation currents at 2 Hz. d) Statistical analysis of CMAP amplitudes induced by different current intensities (*n* = 5). e) CMAPs recorded from the sciatic nerve at different stimulation frequencies. f) Statistical analysis of CMAP amplitudes induced by different stimulation frequencies (*n* = 5). g) Comparison between the captured CMAP signal frequency and the applied stimulation frequency. h) Power spectrum of the recorded CMAP signals. i) Overlay of CMAP signals detected across 8 channels. j) Analysis of amplitude variations in different phases of a single CMAP signal. k) Timeline of the 5‐hour continuous CMAP monitoring experiment. l) Statistical summary of CMAP amplitudes over the 5‐hour monitoring period. m) Heat map summarizing CMAP amplitudes across 8 channels over the monitoring period. n) Segments of CMAP signals recorded across 8 channels during the 5‐hour experiment. o) Statistical summary of pH measurements over 5 h. Using 0 h blood reference data as a baseline, the voltage signals from the RMNEA‐integrated system were converted into pH values according to in vitro standard curves. The yellow star indicates the reference value. p) Heat map summarizing pH measurements across two channels. q) Statistical summary of ROS concentration measurements over 5 h. Using 0 h blood reference data as a baseline, the current signals from the RMNEA‐integrated system were converted into ROS concentrations according to in vitro standard curves. The yellow star indicates the reference value. r) Heat map summarizing ROS concentrations across two channels.

In order to investigate the potential applications of the RMNEA‐integrated system in the detection of muscle fatigue, in vivo physiological signal monitoring experiments were conducted. The subjects were based on anesthetized rabbits, with continuous leg contractions induced by stimulating the sciatic nerve to simulate muscle fatigue. First, the fur on the rabbit's leg was shaved and the skin cleaned with a disinfectant solution. A longitudinal incision was made in the posterior region of the femur and blunt dissection techniques were used to gradually separate the surrounding tissues until the sciatic nerve was exposed. Once the sciatic nerve was exposed, it was gently hooked using double‐hooked electrodes, ensuring that the electrodes were securely positioned without causing any damage to the nerve. The RMNEA‐integrated system was placed on the cleaned leg surface after this preparation. The integrated system was gently pressed to penetrate the stratum corneum and secured with double‐sided adhesive tape. The experimental setup for stimulation and signal monitoring is shown in **Figures** **5**a and S37 (Supporting Information).

Following the application of electrical stimulation to the sciatic nerve, the resulting action potentials were observed to propagate along the nerve fibers, ultimately reaching the motor endplates. This resulted in the release of acetylcholine at the nerve terminals, which in turn led to the contraction of the associated muscle fibers, thus producing observable leg movements.^[^ ^42^ ^]^ A series of charge‐balanced, anode‐first stimulus pulses were delivered through the double‐hooked electrodes, stimulating the sciatic nerve continuously. The stimulation resulted in the occurrence of regular muscle contractions in the rabbit's leg. As illustrated in Figure 5b, the integrated system recorded a series of compound muscle action potential (CMAP) signals. In order to establish an effective muscle fatigue model, it was essential to investigate the effect of different electrical stimulation conditions on the muscle response of the rabbit and identify the optimal current parameters to establish an effective muscle fatigue model. By maintaining a constant stimulation frequency and applying different magnitudes of current, the amplitude of the CMAP increased in proportion to the current intensity. This was because higher current intensities can cause greater neuronal excitation, leading to the release of more neurotransmitters at the nerve terminals. This ultimately induced more intense muscle contractions, leading to an increase in CMAP amplitude.^[^ ^43^ ^]^ When the current reached 32 mA, the CMAP amplitude appeared to saturate (Figure 5c). The neuromuscular system had reached its maximum state of excitation, with all muscle fibers within the motor units fully activated. Consequently, further increases in current did not result in the activation of additional muscle units, and the CMAP amplitude ceased to increase. A plot of CMAP amplitude versus current intensity (Figure 5d) showed that as the current increased from 2 to 32 mA, the CMAP amplitude increased steadily until it reached a saturation point at 32 mA with a maximum amplitude of 1.2 mV.

When the current magnitude was held constant, the application of stimuli at different frequencies demonstrated a positive correlation between the recorded CMAP frequency and the stimulation frequency (Figure 5e). As the stimulation frequency increased, the contraction frequency of the leg muscles also increased. However, when the frequency reaches 100 Hz, the effectiveness of muscle contraction began to diminish. The relationship between CMAP amplitude and frequency (Figure 5f) indicated that the CMAP amplitude remained relatively consistent across the tested frequency range, suggesting that frequency had a limited impact on CMAP amplitude within this range. As illustrated in Figure 5g, there was a one‐to‐one correspondence between the applied stimulation frequency and the recorded CMAP frequency. The CMAP spectral analysis demonstrated that the primary peak frequency of the CMAP remained within the 0–1 kHz range (Figure 5h). When analyzing a single CMAP signal (Figure 5i), a stimulation artifact was observed, followed by the evoked action potential. These artifacts were usually caused by electrical stimulation pulses as they propagate between the electrode and the nerve, rather than being generated by muscle contraction. The single CMAP signal was divided into eight phases and the amplitude of each phase was analyzed statistically. A heat map showing the spatial distribution of CMAP amplitudes across the multi‐channel electrodes (Figure 5j) showed variations in CMAP responses in different regions of the leg muscles. This analysis offered insights into the localized muscle responses and highlighted the differences in CMAP amplitudes across the various regions.

In the experiments exploring the effects of different electrical stimulation conditions on the muscle responses of rabbits, a current intensity of 32 mA was sufficient to induce noticeable muscle contractions, while a frequency of 5 Hz resulted in a muscle activity pattern that was not too frequent. Consequently, continuous electrical stimulation was applied using the parameters above to simulate prolonged muscle activity and induce muscle fatigue. The current intensity was set at 32 mA, while the frequency was set at 5 Hz. During a five‐hour stimulation period on the rabbit's leg muscles, a series of measurements were taken at hourly intervals, including CMAP, muscle pH, and ROS levels. Additionally, blood samples were collected at 0, 2, and 5 h for comparative purposes (Figure 5k). Using the 0 h blood reference data as a baseline, subsequently collected signals were compared to this reference signal to calculate the changes in voltage and current. Based on the slope of the in vitro calibration curve, the changes in voltage and current were converted into corresponding changes in pH and H₂O₂ concentration. As illustrated in Figure 5l, during the initial hour following the commencement of stimulation, the CMAP amplitude remained unaltered, and pronounced muscle contractions were observed. However, following the initial hour of stimulation, a decline in the CMAP amplitude was observed. As the stimulation proceeded, the CMAP amplitude in some channels exhibited a gradual decline. By the third hour of stimulation, the CMAP amplitude in certain channels had diminished to a level that was markedly weaker than that observed at the outset of the experiment. Notably, along with the decrease in CMAP amplitude, some channels exhibited tremor‐like phenomena characterized by rapid, involuntary, small muscle twitches. These tremors may be associated with muscle fatigue and impairments in neuromuscular conduction. The CMAP amplitude heat map (Figure 5m) demonstrated that during the initial 1–2 h of electrical stimulation, the CMAP amplitude across channels exhibited minimal fluctuations, with only slight differences between channels. However, as the stimulation period continued, a gradual decline in CMAP amplitude was observed in some channels, particularly evident in the latter half of the stimulation period. By the fifth hour of stimulation, the CMAP amplitude in some channels had almost reached zero, indicating severe muscle fatigue in those regions. In addition, there were significant differences in CMAP amplitude between channels (Figure 5n). Some channels maintained relatively high amplitudes throughout the stimulation period, whereas others showed a more rapid decline. These discrepancies may be attributed to variations in fatigue sensitivity among different muscle fibers and the electrical stimulation parameters chosen. As illustrated in Figure 5o,p, the pH values recorded from two channels exhibited minimal fluctuations over the five‐hour stimulation period, demonstrating a consistent acid‐base balance within the muscle tissues. This indicated that the acid‐base balance within the muscle tissues remained relatively stable without significant deviation. Figure 5q,r illustrated a divergent trend in ROS levels, which initially exhibited a decline before displaying a subsequent increase. During the early phase of stimulation, the continuous contraction of the leg muscles required substantial energy and oxygen uptake, which was likely to have suppressed the pathways responsible for ROS production. This suppression may have resulted in the observed decrease in ROS levels. However, in the later stages of stimulation, as muscle fatigue set in and contraction responses weakened or ceased, there was a notable rebound in ROS generation. The observed increase in ROS levels post‐fatigue may be attributed to a recovery or overcompensation in metabolic activity once the intense demand subsided.

### 2.6 In Vivo Detection of SNI by RMNEA‐Integrated System

**Figure 6:**
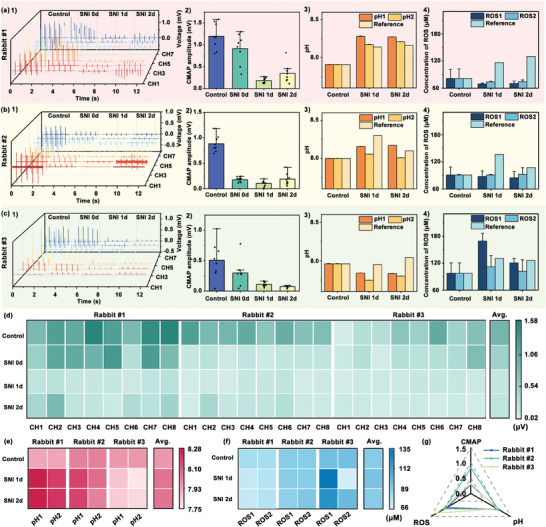
Monitoring of physiological signals of the SNI model. a–c) Monitoring of CMAP, pH, and ROS concentration using the RMNEA‐integrated system in Rabbits #1, #2, and #3: 1) Recorded CMAP signal segments across 8 channels. 2) Statistical analysis of CMAP amplitudes (*n* = 8). 3) Comparative analysis of pH values obtained from RMNEA‐integrated system and standard reference methods. The mean and SD obtained from continuous recording for 2 min. 4) Comparative analysis of ROS levels measured by RMNEA‐integrated system and standard reference methods. The mean and SD obtained from continuous recording for 2 min. d) Heat map of CMAP amplitude fluctuations. e) Heat map of pH dynamics. f) Heat map of ROS dynamics. g) A radar chart summarizing the detection of CMAP, pH, and ROS across the experimental groups. The data were normalized to the control group's maximum values. Data were presented as mean ± SD.

To evaluate the ability of the RMNEA‐integrated system to monitor changes in physiological parameters following SNI and to assess the severity of nerve damage, an experiment was conducted using a rabbit SNI model. The rabbit sciatic nerve was ligated to induce SNI and the RMNEA‐integrated system was used to monitor physiological signals in the leg. Prior to the induction of the model, CMAP, ROS, and pH values were recorded using the RMNEA‐integrated system as control data for comparison with post‐injury data. During the procedure, the sciatic nerve was exposed and gently hooked with double‐hooked electrodes. Electrical stimulation (current amplitude of 2 mA, frequency of 2 Hz, pulse width of 100 µs) was applied to evoke muscle contractions in the leg, and CMAP signals were recorded. Subsequently, the initial pH and ROS values were recorded, after which the sciatic nerve was ligated with sutures to simulate SNI. Subsequently, electrical pulses were reapplied in order to obtain CMAP signals. Once the monitoring of physiological signals had been completed, the RMNEA‐integrated system was removed and the surgical site was cleaned and treated. This included suturing the muscle, subcutaneous tissue, and skin, followed by washing and disinfecting with alcohol. On the first and second days following the model induction, the surgical site was reopened to re‐expose the sciatic nerve, allowing for the re‐recording of CMAP. Concurrently, ROS and pH levels were monitored to further assess the impact of SNI on muscle physiological signals. This experimental procedure was consistently performed on three rabbits, and the corresponding data were collected (**Figure** **6**a–c). Using the reference values of the blood collected from the control group as a baseline, the signals obtained on the second and third days after surgery were compared with this baseline signal to calculate the changes in voltage and current. Based on the slope of the in vitro calibration curve, these changes in voltage and current were converted into corresponding changes in pH and H₂O₂ concentration. The results demonstrated a significant reduction in CMAP amplitude in all three rabbits following SNI. This indicated that nerve ligation hindered or damaged the conduction of nerve impulses, thus impairing muscle activation and contraction responses. On the first day of post‐model induction, a sustained decline in CMAP amplitude was evident in all three rabbits, substantiating the persistent influence of SNI on muscle physiological signals. By the second day post‐model induction, rabbits #1 and #2 exhibited a rebound in CMAP amplitude, while rabbit #3 showed a further reduction. This indicated that rabbits #1 and #2 may have experienced less severe nerve compression damage, thereby enabling partial recovery of nerve function within a relatively short period. In contrast, rabbit #3 was likely to have suffered more severe nerve compression, resulting in the rupture or severe damage of nerve fibers and preventing the prompt recovery of nerve function. In the experiments assessing pH changes, the variations in pH levels between the three rabbits before and after model induction were relatively small. This observation suggested that the model induction process did not affect the overall acid‐base balance of these animals. Specifically, the pH levels of rabbits #1 and #2 showed an upward trend, whereas rabbit #3 showed a slight decrease, indicating individual variability in response to model induction. However, the magnitude of these changes was small and insufficient to detect any clear physiological or pathological changes. For ROS measurements, the trends recorded in both channels were consistent but deviated from the reference values. This discrepancy could be attributed to the localized inflammatory response, which was mainly confined to the surgical site. As the RMNEA‐integrated system was placed on the leg muscle, relatively far from the nerve ligation site, the inflammatory response probably did not extend to the area monitored by the RMNEA‐integrated system. As a result, the recorded ROS levels did not match the expected reference values. Furthermore, other factors may have affected the observed ROS trends, including tissue metabolic activities, or other environmental conditions within the muscle region.^[^ ^44^, ^45^ ^]^ These factors may have interfered to some extent with the assessment of the actual inflammatory response, resulting in a discrepancy between the signals recorded by the RMNEA‐integrated system and the true physiological conditions at the surgical site. The heatmap of CMAP signals across 8 channels in three rabbits (Figure 6d) showed notable trends. Before the sciatic nerve ligation, the CMAP amplitudes were consistently high across all channels, indicative of robust neural activation and muscle response. Immediately following the ligation, a notable decline in CMAP amplitudes was observed across all channels, reflecting the immediate impact of the SNI. On the first day after SNI, there was a further decrease in CMAP amplitudes in all channels, indicating a progressive deterioration in neural function. By the second day post‐injury, a slight recovery in CMAP signals was observed in some channels, though overall signal strength remained weak. The pH heatmap (Figure 6e) demonstrated minimal variation over time. Two rabbits demonstrated a gradual increase in pH levels, while the third exhibited a decrease. This variation may be associated with the inflammatory status of the muscle tissue surrounding the injury. Rabbits with less severe nerve damage could show an increase in pH due to local inflammatory responses leading to alkaline shifts, whereas more severe injuries could result in a decrease in pH due to extensive tissue damage and metabolic acidosis. The ROS heatmap (Figure 6f) demonstrated a sustained increase in H₂O₂ levels in two of the rabbits, which mirrored the rising trend observed in their blood samples. This indicated that the rabbits were experiencing ongoing inflammatory responses. In the third rabbit, the absence of an increase in ROS levels may be attributed to the RMNEA‐integrated system being placed at a greater distance from the injury site, potentially reducing its sensitivity to localized oxidative stress. The radar chart (Figure 6g) provided a comparative analysis of the changes in physiological signals observed post‐injury relative to baseline measurements. The CMAP amplitude was reduced following nerve ligation, indicating a reduction in neuromuscular activity and suggesting impaired nerve function or muscle response. The level of ROS exhibited an upward trend, consistent with increased oxidative stress and inflammation in the injured tissue. In contrast, the changes in pH levels were relatively minor, indicating that the overall acid‐base balance in the tissue fluid remained relatively stable. The RMNEA‐integrated system allowed detailed monitoring of muscle electrophysiological responses, ROS levels, and pH following SNI. These observations were of great importance for the assessment of the extent of neural damage and the tracking of recovery processes. Future research will concentrate on enhancing the performance of the RMNEA‐integrated system, to improve its monitoring precision and stability for wider applications in clinical and research settings.

## 3 Conclusion

In summary, the RMNEA‐integrated system was developed as a wearable device designed for integrated and simultaneous monitoring of electrophysiological signals and biochemical markers in the subcutaneous space, including EMG, ROS, and pH. Through a precise array design and optimized electrode modification layer, the RMNEA‐integrated system overcame the crosstalk issues commonly associated with direct chemical modifications in traditional microneedle patches. It achieved high‐resolution multi‐parameter detection by utilizing individual TSMNs. These TSMNs penetrated the skin's stratum corneum to directly interface with subcutaneous tissues, which not only reduced the impedance of the skin–electrode interface, thereby enhancing the quality of EMG signal acquisition but also facilitated in situ detection of biochemical substances, improving the reliability of biomarker measurements. The electrochemical deposition of the PEDOT: PSS modification layer significantly enhanced the electrochemical performance and signal sensing capabilities of the MNEs, addressing the challenge of limited TSMN surface area. Additionally, the incorporation of pogo pins ensured reliable signal transmission and stable device performance, while allowing for the flexible replacement of microneedles, thereby improving the system's maintainability and longevity. In vivo, testing on animal models further validated the RMNEA‐integrated system's capability to detect subtle changes in muscle electrophysiological activity, ROS responses, and pH balance associated with muscle fatigue and SNI. This demonstrated the immense potential of the RMNEA‐integrated system in the field of muscle disease monitoring, providing significant insights into injury assessment and rehabilitation tracking. Overall, the RMNEA‐integrated system represented a new approach for real‐time, multi‐parameter monitoring of physiological and biochemical indicators in subcutaneous tissues, offering valuable tools for both clinical and research applications in neuromuscular health and beyond.

## 4 Experimental Section

### Preparation of PEDOT: PSS/Au MNEs

The TSMNs (Type 304) were prepared using CNC machining technology and laser welding (Dongguan Chaoyue Metal Materials Precision Products Co., Ltd.). To remove the oxide layer on the surface of TSMN, the sample was treated with an acidic detergent. It was then thoroughly rinsed with deionized water and dried. Subsequently, the sample was immersed in a sodium gold sulfite solution (Yuncaitaotao Company). An ECW (CHI760E, CH Instruments, Inc.) was employed to conduct an electroplating reaction, applying a constant current of −2 mA for 600 s, which resulted in the deposition of a gold layer onto the TSMN. In the next step, the TSMN was immersed in a mixed solution of 0.01 m 3,4‐ethoxylene dioxy thiophene (EDOT) and 0.1 m poly (sodium p‐styrenesulfonate) (NaPSS). Electrochemical polymerization was then performed by applying a constant current of 10 µA for 400 s to deposit a layer of PEDOT: PSS on the Au MNE, forming the PEDOT: PSS/Au MNE. Finally, the electro‐polymerized film was washed with deionized water at room temperature and dried with N₂ spray.

### Preparation of ROS and pH MNEs

For the ROS sensor, the previously fabricated PEDOT: PSS/Au MNE was immersed in a platinum sulfite solution (Yuncaitaotao Company). The MNE surface was coated with platinum nanoparticles via electrochemical deposition, using a constant current of 10 µA for 400 s. This process entailed electrochemical polymerization, which resulted in the deposition of a layer of Pt onto the PEDOT: PSS/Au MNE, thereby forming the ROS‐MNE. For the pH potential sensor, the previously prepared PEDOT: PSS/Au MNE was immersed in a mixed solution of 0.25 m aniline and 0.5 m H₂SO₄. Subsequently, electrochemical deposition was conducted by performing 20 CV cycles in the dispersion solution at a scan rate of 100 mV s^−1^, cycling between −0.2 and 1 V. This process resulted in the deposition of a layer of PANI onto the Au MNE, thus forming the pH‐MNE.

### Preparation of Counter MNE and Reference MNEs

To fabricate the counter electrode, the Au MNE was immersed in a platinum sulfite solution (Yuncaitaotao Company). An ECW (CHI760E, CH Instruments, Inc.) was employed to electrodeposit a Pt layer onto the Au MNE at a current of −2 mA for 1000 s. This resulted in the formation of the Pt/Au MNE, which was subsequently used as the counter MNE. The reference electrode was fabricated using a dip‐coating method. The surface of the Au MNE was coated with Ag/AgCl slurry (JY‐20, Dongfang Building Materials). Subsequently, the Ag/AgCl/Au MNE was dried at 80 °C for a period of 30 min. PVB (79.1 mg) and NaCl (50 mg) were dissolved in 1 mL of methanol, and then the Ag/AgCl/Au MNE was impregnated three more times in the solution. The PVB/Ag/AgCl/Au MNE was obtained through dip‐coating, after which the modified MNE was left to dry overnight at room temperature.

### Electrochemical Characterization of PEDOT: PSS/Au MNEs

An ECW (CHI760E, CH Instruments) was used to perform in vitro electrochemical performance tests on the PEDOT: PSS/Au MNEs. The tests included CV and electrochemical impedance spectroscopy (EIS). A three‐electrode configuration was used for these tests, with a platinum electrode as the counter electrode and the Ag/AgCl MNE as the reference electrode. In the EIS test, the electrolyte employed was a 10 mm PBS solution with a pH level of 7.4. A sinusoidal perturbation of 10 mV was applied, and the impedance was measured across a frequency range from 1 Hz to 1 MHz, with the DC potential fixed at 0 V. For CV, the potential was scanned from −0.3 to +0.3 V at a rate of 100 mV s^−1^. In the first cycle, the CSCc was calculated by integrating the current over the potential range from −0.3 to 0.3 V.

### Electrochemical Characterization of ROS‐MNEs

The electrochemical performance of the ROS‐MNEs was characterized in vitro using an ECW (CHI760E, CH Instruments). Four electrochemical techniques were used for a comprehensive analysis: CV, DPV, SWV, and *i–t*. The standard three‐electrode configuration was used, with a ROS‐MNE as the working electrode, a Pt electrode as the counter electrode, and an Ag/AgCl MNE as the reference electrode. The concentration of H₂O₂ in the solution was gradually increased to 2, 4, 6, 8, and 10 mmol L^−1^ to evaluate the H₂O₂ response. In the CV tests, the potential range was set from −0.4 to +0.4 V, with a scan rate of 100 mV s^−1^. For DPV measurements, the potential range was set to −0.4 to +0.4 V, with a pulse amplitude of 50 mV, a pulse width of 50 ms, and a pulse period of 500 ms. In the SWV analysis, the potential range was adjusted from −0.6 to +0.6 V, with a voltage increment of 4 mV, an amplitude of 30 mV, and a frequency of 50 Hz. During *i–t* measurements, a constant potential close to the H₂O₂ reduction peak at −0.1 V was applied, and the current was recorded over time. To assess the impact of potential interferents on H₂O₂ detection, PBS solution was supplemented with 2 mm NaCl, 2 mm KCl, 2 mm lactic acid, or 2 mm glucose. After the addition of 5 mm H₂O₂, the measurements were repeated after the introduction of the same concentration of interferents as in the first round. This allowed the influence of these substances on the H₂O₂ sensing capabilities of the MNE to be analyzed.

### Electrochemical Characterization of pH‐MNEs

The ECW (CHI760E, CH Instruments) was used to test the in vitro electrochemical performance of the pH‐MNE. OCP measurements were used to assess the response of the pH‐MNE. A buffer solution with a pH range of 5–8 was prepared and calibrated using a commercial pH meter. To assess the selectivity of the pH‐MNE, potential interfering substances were tested in deionized water. These substances included 2 mm NaCl, 2 mm KCl, 2 mm CaCl₂, or 2 mm glucose. After the addition of these interferents, 5 mm lactic acid was added to the solution. The procedure was repeated with the same set of interfering substances to analyze their effect on the pH measurements.

### Characterization and Elemental Analysis of MNE Surface Morphology

The surface morphology and microstructure of the prepared sensing MNEs were examined using a field emission SEM (SUPRA 60, Zeiss). In addition, an EDX system (Oxford Instruments) was used to analyze the elemental composition of the electrode materials. A working voltage of 10 keV was chosen for the EDX tests to achieve optimum resolution and effective detection of the elements of interest. In addition, photographs were taken using a standard camera to provide a macroscopic view of the assembled RMNEAs.

### Characterization of the Penetration Properties of TSMNs

A 2 mg mL^−1^ rhodamine B solution was evenly applied to the tips of the TSMNs using a cotton swab. The TSMN tips were then positioned perpendicular to the surface of the pig skin, and the RMNEA was firmly inserted into the pig skin for 5 min before being removed. The treated skin was examined under a fluorescence microscope (MF41, MSHOT) to observe the transdermal delivery facilitated by the TSMNs. To verify the skin penetration capabilities of the TSMNs, the puncture sites along the TSMNs were dissected with a scalpel to obtain ≈1 mm thick sections of porcine skin. These sections were then placed under a fluorescence microscope for further examination. In addition, small squares of pig skin with needle punctures were cut out, dehydrated, and dried. A thin gold layer, with a thickness of ≈10 nm, was deposited onto the surface of the pig skin. The microstructure of the skin after TSMN penetration was observed using an SEM.

### Mechanical Testing of TSMN Penetration into the Skin

The RMNEA was then attached to the probe of a force gauge (HP‐20, Yueqing Handpi Instruments Co., Ltd.). A fresh porcine skin sample was selected and positioned on the platform under the force gauge to ensure stability during force application. The force gauge was connected to an electric moving stage (GCD‐203050 M, Daheng), which controlled the downward movement at a constant speed of 0.2 mm s^−1^, driving the RMNEA vertically into the pig skin. Upon complete insertion into the pig skin, the movement of the stage was stopped and the RMNEA was allowed to remain embedded in the skin for 3 s. After the specified dwell time, the motorized stage was reactivated to withdraw the TSMNs vertically from the pig skin at the same constant speed as insertion. Throughout the experiment, force gauge data was continuously transmitted to a data acquisition system for real‐time analysis.

### Simulation of TSMN Penetration through the Skin

A finite element model was developed using COMSOL Multiphysics software with the objective of investigating the mechanical behavior of TSMNs during skin penetration. A 2D model was constructed, representing the cross‐sectional view of both the TSMN and the skin. The TSMN was simplified to a trapezoidal geometry, while the skin was divided into two layers: the stratum corneum, with a thickness of 20 µm, and the dermis, with a thickness of 1.5 mm. The physical parameters settings of the skin can be found in the Supporting information. The TSMN was modeled as a rigid body, whereas the skin layers were treated as elastic bodies, adopting the Kelvin–Voigt material model to accurately represent their behavior under large deformation conditions. In the simulation, the boundaries of the skin model at the bottom and side were fixed to prevent displacement during the insertion of the TSMN. A refined mesh was applied specifically to the contact area between the TSMN tip and the skin surface to accurately capture stress concentration and deformation. A contact pair was defined between the TSMN and the skin to simulate their dynamic interaction. The TSMN was subjected to a vertical displacement load, simulating its insertion into the skin at a constant speed of 10 µm s^−1^. Upon reaching an insertion depth of 800 µm, the process was halted to simulate a dwell time, after which the TSMN was withdrawn at the same speed. Transient analysis was performed within the solid mechanics module to model the dynamic response of the skin as the TSMN penetrated and was subsequently withdrawn.

### Impedance Testing and Data Fitting of MNEs to Skin

Two TSMN spaced 5 mm apart were precisely inserted into the epidermis of a rabbit to a depth of 800 µm. An ECW (CHI760E, CH Instruments) was employed to perform impedance measurements over a frequency range of 1 Hz to 100 kHz. During the testing process, particular attention was paid to maintaining the stability and contact of the MNEs with the epidermis in order to ensure the accuracy and reliability of the measurements. To minimize the potential for experimental error, each test was conducted three times, and the results were recorded and averaged. Subsequently, the empirical data obtained from the microneedle–skin interface were imported into the ZView (Scribner Associates Inc.) software for data fitting. This procedure enabled the extraction of specific parameters related to the equivalent circuit model of the microneedle–skin interface.

### Design and Development of the Detection Device

To ensure a compact design, the device was divided into two stacked PCBs, each with a height of 1.2 mm and a radius of 14.5 mm. The accurate alignment of the components during the assembly process was facilitated by the utilization of male (7.2 mm, Honger Technology Co., Ltd.) and female (6.5 mm, Honger Technology Co., Ltd.) connectors on each PCB. One PCB was equipped with male connectors, while the corresponding female connectors were utilized on the other PCB. This design ensured the precise mating of the connectors, thereby preventing incorrect connections. The upper PCB exhibited a complex configuration, integrating an MCU, an electrochemical AFE, a power management module, and a Wi‐Fi communication module. The MCU (STM32F411CCU6TR, STMicroelectronics) was programmed on board using an ST‐LINK programmer and acted as the central coordinator for the device's various functional modules. The electrochemical AFE (AD5940BCBZ‐RL, Analog Devices) was responsible for electrochemical signal processing, supporting a range of measurement types and resolution modes. A multiplexer dynamically connected the RMNEA sensors to the appropriate channels, ensuring that the conditioned voltage remained within the detectable range of the ADC. In the board layout of the AFE, analog ground (AGND) and ground (GND) were isolated to minimize interference. The power management module stabilized the device's power supply by utilizing a low dropout regulator (TPS7A2133PYWDJ, Texas Instruments) to convert the voltage of the lithium battery to a consistent 3.3 V for the MCU, the Wi‐Fi communication module, and two AFE chips. In addition, a low‐noise positive and negative output charge pump (LM27762DSST, Texas Instruments) converted the 3.3 to ±2.5 V, providing the required analog power for the EMG AFE chip (ADS1299, Texas Instruments). The Wi‐Fi communication module (ESP8285, Espressif Systems (Shanghai) Co., Ltd.) enabled wireless data and command transmission between the computer and the detection device.

The lower PCB was equipped with the EMG AFE chip, which processed the collected EMG signals. To suppress baseline drift, an RC low‐pass filter (cut‐off frequency at 1 Hz) was placed in front of the AFE. This AFE supported differential input, signal amplification (Gain = 24), and ADC conversion of the EMG signals, sampling at a rate of 2 kHz. To ensure a secure and reliable connection to the TSMN array at the base, the underside of this PCB was fitted with gold‐plated pogo pins (2 mm, Shenzhen Guangchen Precision Hardware Co., Ltd.). This design not only ensured stable connections but also increased the durability of the system. A 3.7 V lithium‐ion battery (501 220, Shenzhen Zhongshunxing Energy Battery Co., Ltd.) was used as the device's power supply, positioned between the two PCBs. The PCBs were fabricated by Shenzhen Jialichuang Technology Group Co., Ltd. Various components were affixed using solder paste (XGSP80, MECHANIC), and reflow soldering was performed at 200 °C.

### Characterization of the Detection Device

To validate the EMG measurement performance of the detection device, a waveform generator was connected to the EMG input of the detection device. The generator produced sine waves with frequencies ranging from 0.1 to 100 Hz and an amplitude of 8 mV. Data acquisition was then performed using a computer. The Extract Single Tone Information VI in LabVIEW (National Instrument Corp.) was used to extract the signal component with the largest amplitude from the collected data. The ratio of this signal component to the preset amplitude of 8 mV was calculated to evaluate the voltage gain of the circuit at different frequencies. A similar RC filter circuit model was constructed in Multisim (National Instrument Corp.) to simulate the frequency response of the detection device. The simulation results were compared with the actual voltage gain measurements obtained at different frequencies to validate the frequency response of the detection device. SNR analysis was performed for eight channels using SINAD VI in LabVIEW. To evaluate the performance of the detection device in electrochemical measurements, a comparative analysis was conducted with an ECW (CHI760E, CH Instruments). Measurements were conducted under OCP and *i–t* conditions, with the results obtained from the detection device and the ECW being compared. For power consumption analysis, a power analyzer (EKA1080 M, Yingjia Technology) was used to characterize the power consumption of the detection device. Power consumption data was collected at a sampling rate of 1 Hz to generate power consumption curves for the detection device under different operating conditions. An infrared camera (K09, HIKMICRO) was used to monitor the temperature distribution during the detection device operation. A digital multimeter (FLUKE 117C, Fluke) was used to measure the lithium battery voltage.

### RMNEA‐Integrated System Integration

The base and cap were designed using 3ds Max software (Autodesk, USA). The base was designed with 16 pre‐set holes to securely hold the TSMN, ensuring they were firmly inserted and fixed in place, preventing any displacement. The length of TSMNs exposed from the base could be adjusted by varying the thickness of the base bottom. The base incorporated side slots designed to latch the cap, thereby ensuring a secure and tight closure. The fabrication of the base involved the utilization of CNC machining technology, which was employed to carve, grind, and polish acrylic sheets, thus completing the production of the base. The cap was manufactured using SLA photopolymer resin model 8001, through 3D printing technology provided by Shenzhen Jialichuang Technology Group Co., Ltd. The design of the adhesive tapes was created using AutoCAD software (Autodesk, USA), which was then processed from 3 m medical adhesive tape using laser cutting technology (Shenzhen Yichen Adhesive Tape Products Co., Ltd.). The processed tapes were then adhered to the base to ensure a secure fit. Each TSMN in the array was then meticulously positioned on the base. The electronic circuitry was then positioned inside the base, ensuring that the pogo pins were precisely aligned with the TSMNs beneath them. Finally, the cap was placed on the base and snapped into place, completing the assembly of the prototype.

### Animal Studies

Animal studies were conducted under strict ethical guidelines and all protocols were approved by Guangzhou Boyao Biotechnology Co., Ltd. (Approval No. IAEC‐K‐240221‐02) and the Institutional Animal Care and Use Committee of Sun Yat‐sen University (Approval No. SYSU‐IACUC‐2024‐B0814). Five adult male New Zealand rabbits weighing between 2.5 and 3.0 kg were purchased from Guangzhou Boyao Biotechnology Co., Ltd. The rabbits were anesthetized with an intravenous injection of 15 mg k^−1 ^g Zoletil50 and an intramuscular injection of 6 mg k^−1 ^g Sumianxin II. After anesthesia, the rabbits were covered with blankets to maintain body temperature. Continuous anesthesia was maintained by additional injections of Zoletil50 into the leg. Leg hair was removed using surgical scissors and depilatory cream. At the end of the measurements, the animals were euthanized by an overdose of Zoletil50 by intramuscular injection. To determine the concentrations of H₂O₂ and pH levels in the blood of the rabbits, venous blood samples were taken from the leg and centrifuged to extract the serum. Serum H₂O₂ concentration was measured using the CheKine Catalase Activity Assay Kit (Abbkine), and pH was assessed using the LabSen241‐3A micro pH electrode (SANXIN).

### Detection of Physiological Signals in Muscle Fatigue Models

A 3–4 cm incision was made along the course of the sciatic nerve at the back of the leg. The sciatic nerve was carefully exposed using blunt dissection techniques. A double‐hooked electrode (KD‐BHE, Kedou Brain–Machine Technology Co., Ltd.) was gently hooked around the sciatic nerve, ensuring close contact between the electrode and the nerve while avoiding excessive tension to prevent nerve damage. Contact with surrounding muscle tissue was also avoided. The double‐hooked electrodes were then connected to an electrical stimulator (Hangzhou Nuanxinjia Electronics Technology Co., Ltd.), which was set to deliver a biphasic square wave stimulation with a half‐phase period of 200 µs to induce leg twitching through sciatic nerve stimulation. The RMNEA‐integrated system was placed on the depilated area to ensure it was in close contact with the skin and able to penetrate the stratum corneum. After one hour of electrical stimulation at 32 mA and 5 Hz to induce muscle contractions in the leg, the RMNEA‐integrated system recorded the CMAP signals generated. Immediately after recording the CMAP signals, the electrical stimulation was stopped to prevent interference with the subsequent detection of ROS and pH signals. Under conditions free of electrical stimulation interference, the RMNEA‐integrated system recorded ROS and pH signals for ≈3 min each. The mean values of these two signals during their respective recording periods were calculated as the final analysis results. To obtain reference values for blood pH and H₂O₂ concentrations, venous blood samples were taken from the leg at 0, 2, and 5 h. The 0‐hour blood reference data was used as the baseline for calibrating measurements obtained from the RMNEA‐integrated system, enabling comparison between in vitro and in vivo experimental results. Subsequent signals were compared to this baseline to calculate changes in voltage and current. Using the slope of the in vitro calibration curve, these variations in voltage and current were then converted into corresponding changes in pH levels and H₂O₂ concentrations.

### Detection of Physiological Signals in a SNI Model

The sciatic nerve of New Zealand rabbits was surgically exposed by an incision along the posterior leg and gently dissected. Double hooked electrodes were carefully applied to the exposed sciatic nerve and electrical stimulation was applied to evoke CMAP. The RMNEA‐integrated system was used to record the CMAP while simultaneously monitoring the levels of ROS and pH in the associated muscle tissue as control data. Venous blood samples were taken from the leg to determine blood pH and H₂O₂ concentration to provide reference data. To simulate the SNI model, the sciatic nerve was ligated three times with surgical sutures (Shanghai Pudong Jinhuan Medical Products Co., Ltd.). The tension of the sutures was carefully adjusted to induce slight twitching of the calf muscles or toes to ensure adequate tightness. During the initial assessment after modeling, electrical pulses were applied to record CMAP. After this recording, the double‐hooked electrode and the RMNEA‐integrated system were removed. The surgical area was then thoroughly irrigated with saline, and the muscle, subcutaneous tissue, and skin layers were sutured sequentially, followed by alcohol disinfection. On the second and third days after surgery, the sutures were removed and the RMNEA‐integrated system was reapplied to the leg muscles. Double‐hooked electrodes were used to stimulate the sciatic nerve, inducing leg muscle contractions and generating CMAP signals. The RMNEA integrated system was used to record both CMAP and electrochemical measurements. The baseline was established using blood reference values collected from the control group. Signals collected on the first and second days post‐model were then compared with this baseline to calculate changes in voltage and current. Based on the slope of the in vitro calibration curve, these variations were converted into corresponding changes in pH levels and H₂O₂ concentrations. Venous blood samples were collected during each session in order to measure blood pH and H₂O₂ concentrations. These samples provided reference data for each measurement.

### Animal Experiment Data Analysis and Statistics

For CMAP signal processing, no filtering was applied to the signal. The WA Multiscale Peak Detection VI in LabVIEW was directly used to extract the peaks and troughs of five CMAP waveforms. The amplitude was determined by calculating the difference between each peak and valley and then taking the average of these differences to obtain the CMAP amplitude. For ROS or pH sensing data processing, the initially acquired signal was set as the reference signal, and the corresponding ROS concentration or pH value was set to the blood‐measured reference value. In subsequent data acquisitions, the difference between the newly acquired signal each time and the reference signal was utilized to calculate the change in ROS concentration or pH. The conversion was carried out using the following equation:

(1) $$y\;=\frac{\Delta x}{k}+y_{0}$$

where *y* represented the calculated ROS concentration or pH value, Δ*x* was the difference between the current signal and the reference signal, and *k* was the slope derived from the in vitro calibration curve.

### Statistical Analysis

Figure 1a was created using Servier Medical Art templates, which were licensed under a Creative Commons Attribution 3.0 Unported License. All the statistical analyses were conducted and visualized using Origin 2023. The acquired data were calculated and expressed as the mean ± SD. The differences between the two compared groups were determined by a *t*‐test. *P*‐values < 0.05 indicated that the results were considered statistically significant. *p < 0.05, **p < 0.01, ***p < 0.001 and ****p < 0.0001.

## Conflict of Interest

The authors declare no conflict of interest.

## Supporting information



Supporting Information

## Data Availability

The data that support the findings of this study are available from the corresponding author upon reasonable request.
