# Supplementary material for: Wearable Systems of Reconfigurable Microneedle Electrode Array for Subcutaneous Multiplexed Recording of Myoelectric and Electrochemical Signals
Source: Adv Sci (Weinh). 2024 Dec 16;12(24):2409075. doi: 10.1002/advs.202409075 (PMC12199586; doi:10.1002/advs.202409075)
Supplement: Supplementary file 1 — Supporting Information [file ADVS-12-2409075-s001.docx]

Supporting Information

Wearable systems of reconfigurable microneedle electrode array for subcutaneous multiplexed recording of myoelectric and electrochemical signals

Zhengjie Liu, Chuanjie Yao, Xingyuan Xu, Xinshuo Huang, Shuang Huang, Shantao Zheng, Tao Zhang, Yan Li, Fangmao Liu, Yuxiang Wu, Jing Liu, Hui-jiuan Chen*, Xi Xie*

Z. Liu, C. Yao, X. Xu, X. Huang, S. Zheng, H. Chen, X. Xie

State Key Laboratory of Optoelectronic Materials and Technologies; Guangdong Province Key Laboratory of Display Material and Technology; School of Electronics and Information Technology; Sun Yat-Sen University, Guangzhou, 510006, China

E-mail: chenhuix5@mail.sysu.edu.cn, xiexi27@mail.sysu.edu.cn

S. Huang, T. Zhang, X. Xie

School of Biomedical Engineering, Sun Yat-Sen University, Shenzhen, 518107, China

Y. Li

Department of Cardiology, The First Affiliated Hospital of Jinan University, Guangzhou, 510630, China

F. Liu

Division of Hypertension and Vascular Diseases, NHC Key Laboratory of Assisted Circulation and Vascular Diseases, The First Affiliated Hospital, Sun Yat-sen University, Guangzhou, 510080, China

Y. Wu

Institute of Intelligent Sport and Proactive Health, Department of Health and Physical Education, Jianghan University, Wuhan, 430056, China

J. Liu

Institute of Precision Medicine, The First Affiliated Hospital, Sun Yat-sen University, Guangzhou, 510080, China

**Section 1.** Discussion on the advantages of RMNEA-integrated system.

**Note S1.** Discussion on the advantages of RMNEA-integrated system in terms of specificity, sensitivity, or scalability

The novelty of RMNEA integrated system lies in its effective integration of multiplexed microneedles electrodes through a reconfigurable design. Although theoretically multiplexed microneedle integration is a straightforward approach, the fabrication of multi-parameter and multi-channels microneedle array has been difficult to achieve in practical preparation. This is mainly due to the need of chemical modifications on microneedles to detect specific biochemical markers. Simultaneous fabrication of multiple (>10) high-quality electrochemical microneedle electrodes in a compact array is challenging, while a few underperforming microneedles may necessitate re-fabrication of the entire array, complicating stable fabrication of multi-channel electrochemical microneedle sensors.

In terms of specificity, previous systems have typically been limited to detecting a single type of signal. In contrast, the RMNEA integrated system is capable of simultaneously monitoring multiple distinct physiological indicators. A time-separated detection mechanism is employed, carefully designing collection times for electrophysiological and electrochemical signals to minimize cross-interference, thereby enhancing the specificity and quality of the collected data. In conventional microneedle sensing systems, the preparation of multi-parameter electrochemical sensors is challenging due to the need for chemical modifications to detect specific biochemical markers. This often reduces fabrication yield, particularly in multiplexed electrode array, where a few underperforming microneedles may necessitate re-fabrication of the entire array, complicating stable fabrication of multi-channel electrochemical microneedle sensors. In order to address this issue, the RMNEA integrated system utilizes a reconfigurable microneedle array structure, allowing individual microneedle modification. If any microneedle does not meet performance criteria during the modification process, only the affected microneedle is replaced, eliminating the need to remake the entire array. This approach significantly enhances the fabrication efficiency of multi-channel electrochemical microneedle arrays.

Regarding sensitivity, microneedles provide distinct advantages over traditional non-invasive planar electrodes. The microneedles penetrate the stratum corneum, thereby bypassing the high impedance stratum corneum layer and reducing signal interference, resulting in significantly improved electrophysiological signal sensitivity. They can also directly detect chemical signals in interstitial fluid, yielding results highly correlated with blood markers. The RMNEA integrated system utilizes high-sensitivity AFE chips and active microneedle electrode designs, which shorten the signal path from electrode to amplifier, reducing external noise interference and ensuring a high signal-to-noise ratio. To further enhance the sensitivity of electrochemical detection, PEDOT:PSS coating was applied to the electrodes, which lowers impedance and increases electrochemical sensitivity. Consequently, the system can obtain high-quality electrophysiological and electrochemical signals even with a small sensing area.

In terms of scalability, the RMNEA integrated system's reconfigurable design allows flexibility in adjusting the sensor array by altering substrate design, sensor count, spacing, and layout. Additionally, different microneedles can be substituted based on the type of signal being detected, allowing for customized adaptations across a range of physiological monitoring scenarios. This reconfigurability also significantly improves system yield. In conventional microneedle sensor systems, electrochemical sensors often require surface modifications, which can reduce yield during fabrication, especially in large arrays where a single underperforming microneedle may necessitate complete re-fabrication. With the reconfigurable microneedle array, modifications are limited to individual microneedles, allowing easy replacement of a single microneedle without the need for full array reconstruction. The reconfigurable array design also supports reuse. When certain microneedles degrade over time, only those microneedles are replaced while retaining the functional ones. This approach extends sensor lifespan and efficiency while reducing system costs. Compared to existing microneedle systems, this reconfigurable design provides enhanced adaptability across various applications, increases system yield, and optimizes sensor utilization, making the RMNEA integrated system a promising innovation in microneedle sensing.

For wireless data transmission, the system uses Wi-Fi technology to achieve a high EMG sampling rate of 2 kHz per channel, supporting large-scale real-time data transfer. This approach overcomes the bandwidth limitations of other systems, making it particularly suitable for multi-channel, high-speed signal acquisition, while ensuring portability and scalability. These features highlight the advantages of this system in terms of miniaturization, sensitivity, sampling rate, and wireless transmission, offering a more efficient solution for multi-parameter monitoring.

In summary, the RMNEA integrated system not only integrates the strengths of existing technologies but also introduces innovations in detection modes, signal processing, and system architecture, addressing previous technical limitations in multi-parameter microneedle sensing. This combination enables the RMNEA integrated system with enhanced sensitivity and adaptability in physiological monitoring, establishing it as a flexible and reliable platform for high-precision health monitoring applications in the future.

**Note S2.** Discussion on the significance of multi-parameter detection in comparison to single purpose systems.

Compared to single-purpose systems, multi-parameter detection systems enable comprehensive monitoring of the physiological state of target areas across multiple dimensions. This approach provides broader data support for assessing complex physiological conditions, as single-purpose systems often yield only partial information. In clinical settings, many physiological and pathological processes result from the interaction of multiple factors. Hence, simultaneous monitoring of these parameters facilitates real-time understanding of multiple physiological signals and their synergy, enhancing the evaluation of disease progression and treatment efficacy. Taking polymyositis as an example, while EMG can reveal its electrical characteristics, such as short duration, low amplitude, and polyphasic waves, these features can also appear in other muscle diseases like muscular dystrophy and metabolic myopathies. As a result, EMG is frequently inadequate for establishing a definitive diagnosis. In such cases, the combination of biochemical tests, such as the measurement of creatine kinase and lactate dehydrogenase levels in the blood, can provide crucial information about the extent of muscle damage and inflammation activity, aiding in the differentiation between various diseases. Moreover, the simultaneous detection of EMG and metabolic biomarkers further supports rehabilitation and outcome evaluation. For example, in treating patients with mitochondrial myopathies, EMG can assess muscle excitability and damage severity, while biochemical assays reveal pathological states in affected areas, such as oxidative stress and levels of inflammatory markers. Based on this combined information, clinicians can formulate more precise and effective rehabilitation training and nutritional support plans to improve patients’ muscle function and metabolic condition.

Due to variations in activity patterns and metabolic levels across different muscle sites, a single-channel detection method often falls short of fully analyzing muscle function. Thus, developing a multimodal sensing system capable of monitoring both electrophysiological and biochemical signals across multiple channels is crucial. The RMNEA integrated system, with its multi-parameter and multichannel capabilities, enhances flexibility and scalability in practical applications compared to systems with single parameters or channels. The RMNEA integrated system employs time-separated acquisition for multi-parameter detection, which prevents interference between electrophysiological and electrochemical signals. This design ensures the independence of each signal channel, avoiding crosstalk among different signal types.

The RMNEA integrated system incorporates a highly integrated AFE chip that not only supports multi-parameter detection but also achieves multichannel electrochemical signal detection through an internal switch matrix, significantly increasing system adaptability. For both electrophysiological and electrochemical monitoring, the RMNEA integrated system performs multi-parameter detection via a single deployment, and software commands facilitate the switching of detection parameters and channels, allowing users to select either electrophysiological or electrochemical detection as needed. Additionally, the RMNEA integrated system offers substantial scalability for different physiological monitoring scenarios.

The RMNEA integrated system allows flexible adjustment according to various detection signal types by requiring only partial microneedle replacement, eliminating the need to reassemble the entire device. Specifically, microneedles can be chemically or physically modified according to the type of signal being monitored, enabling the RMNEA integrated system to quickly adapt to a variety of applications, including electrophysiological signal detection, electrochemical analysis, and other physiological signal monitoring needs.

**Note S3.** Discussion on the integration of electrochemical sensors and real-time monitoring capabilities in multi parameter detection.

In electrochemical detection, single-parameter systems can be categorized into designs featuring either one microneedle per channel or multiple microneedles per channel. The integration of an electrode with a single microneedle poses challenges for electrical connections due to the microneedle's small dimensions. Several solutions have been proposed in the literature. For instance, the paper "Microneedle pH Sensor: Direct, Label-Free, Real-Time Detection of Cerebrospinal Fluid and Bladder pH" described the use of copper wires soldered to W-shaped microneedles, which are then connected to the detection instrument. Another study, "Conformable microneedle pH sensors via the integration of two different siloxane polymers for mapping peripheral artery disease" utilized magnetron sputtering to deposit a metal layer on a PDMS substrate, achieving electrical connection with the microneedles. While both methods enable connection, the longer distance to the detection device may introduce significant noise and signal loss during transmission.

In contrast, a design featuring multiple microneedles per channel offers a larger surface area and facilitates electrical connections. For example, the study "Reduced Graphene Oxide Nanohybrid–Assembled Microneedles as Mini-Invasive Electrodes for Real-Time Transdermal Biosensing" achieved this by using alligator clips to secure metal microneedle patches to the detection device. Additionally, the research "Microarrow sensor array with enhanced skin adhesion for transdermal continuous monitoring of glucose and reactive oxygen species" employed extended leads in its microneedle patch design to serve as an interface for electrical connection to a PCB.

Single-parameter microneedle detection effectively avoids crosstalk issues during the modification process, as each microneedle's modification layer can be independently controlled, ensuring the realization of specific functions. However, in multi-parameter microneedle detection, different layers are often required for various indicators, which may lead to interference between adjacent microneedles. Furthermore, due to the small size of the microneedles, the performance of nearby microneedles may be affected during the modification of a single microneedle. Utilizing the approach of modifying a single microneedle before integration can effectively mitigate this crosstalk issue. However, conventional connection methods used for integrating microneedles into detection circuits, such as inserting into PCBs, soldering, or using conductive silver paste, present certain limitations. When the performance of a microneedle is suboptimal or damaged, it may result in a decline in the yield of the entire system. To address these challenges, the RMNEA integrated system adopts a reconfigurable design that enhances the integration accuracy of multi-parameter single microneedles, thus improving the fabrication yield of the microneedle array system. The RMNEA integrated system allows for the flexible replacement of microneedles, enabling rapid adjustments of sensor functionalities in response to varying monitoring demands without necessitating the reconstruction of the entire system.

Single-parameter microneedle detection is typically employed for real-time monitoring, such as blood glucose level detection, providing immediate feedback. However, in multi-parameter detection, different electrochemical parameters may require the application of distinct biases, potentially leading to crosstalk between measurements. To mitigate this issue, a time-sequenced method can be adopted, alternating the collection of different parameters. This approach effectively avoids interference between measurements, ensuring the accuracy of each parameter. Moreover, not all indicators necessitate real-time collection. In multi-parameter systems, the acquisition frequency can be adjusted according to specific application requirements. This flexibility enables multi-parameter detection to maintain data quality while better adapting to diverse clinical needs.

The RMNEA integrated system utilizes the internal switch matrix of the AFE to facilitate multi-path electrochemical signal detection. This design allows for switching and collection of multiple electrochemical parameters within the same system without the need for additional hardware. Users can send commands via software to switch detection parameters and channels, enhancing the system's operational convenience and providing a more comprehensive and efficient solution for physiological monitoring.

**Note S4.** Discussion on how RMNEA integrated system promotes the development of existing microneedle systems.

In terms of detection functionality, the proposed system integrates dual-modal monitoring of electrophysiological and electrochemical signals, overcoming the limitations of traditional microneedle systems that typically support only single-modal detection. By employing a time-division approach, interference-free detection of different signals was successfully achieved. This simultaneous monitoring of multimodal signals holds significant value for the real-time assessment of complex physiological states.

Regarding signal quality, the system compactly integrates the sensing circuitry above the microneedles, thus shortening the signal transmission path from the sensors to the amplifiers. This design of single microneedle active electrodes not only minimizes noise interference but also enhances the stability of signal transmission. Compared to conventional microneedle systems that connect via long wires, this configuration significantly improves the system's signal-to-noise ratio and reliability. Both electrophysiological and electrochemical detections utilize an AFE chip, greatly reducing the size of the detection circuitry while effectively mitigating noise interference, thereby enhancing the signal quality, detection sensitivity, and reliability of the sensors. Furthermore, the internal switch matrix of the AFE chip was effectively utilized, enabling the system to perform multi-channel electrochemical signal detection. Although the application of PEDOT:PSS materials has been widely reported, this work further optimized their use in microneedle electrodes, significantly reducing electrode impedance and improving signal quality.

In terms of application flexibility, the modular design connected by pogo pins allows for independent maintenance and replacement of the sensors, substrates, and circuitry. This reconfigurable design enables adjustments to the number, spacing, and arrangement of sensors in the array by modifying the substrate design while facilitating the replacement of microneedles according to the type of detected signal, thereby accommodating various physiological monitoring scenarios.

Concerning system fabrication, the TSMNs can be mass-produced, and the reconfigurable design significantly enhances the yield of the system. In existing microneedle sensor systems, the modification of electrochemical sensors often requires alterations to the microneedle surface, which can lead to a decrease in yield, particularly in large-scale sensor arrays. If the performance of a particular microneedle falls short, the entire sensor array may need to be re-fabricated. To address this challenge, the reconfigurable microneedle array allows for individual microneedle modification. Any underperforming microneedle can be replaced without re-fabricating the entire array. The reconfigurable design also enables reuse, whereby individual microneedles can be replaced if performance declines, extending sensor lifespan and reducing system costs. The system proposed in this work significantly advances the current state of microneedle systems through the integration of multiple technologies.

**Table S1.** Comparison between RMNEA-integrated system and existing microneedle sensing systems

| Characteristic | Conventional method | Our advantage |
| --- | --- | --- |
| Microneedle Fabrication | Complex process requiring photolithography, etching, and other expensive, precise instruments | Simple fabrication using tack-shaped metal microneedles, relying on cost-effective mechanical preparation, facilitating mass production and large-scale application |
| Detection Mode | Single-mode detection, typically used for either electrophysiological or electrochemical measurements | Dual-mode detection for simultaneous monitoring of both electrophysiological and chemical signals |
| System Integration | Microneedles are firmly attached to the substrate, unable to be independently detached or reassembled | Reconfigurable design where microneedles, substrate, and circuitry are modular and can be freely combined, detached, or replaced as needed |
| Detection Circuit | Microneedles are connected to the circuit board with long leads, resulting in extended transmission distances  Typically requires larger separating devices for constructing the detection circuit, leading to a bulkier system | Circuitry positioned above the microneedle array, forming an active electrode structure that minimizes interference from long wiring  The use of AFE chips further minimizes the overall size of the detection system |
| Yield Rate | Integrated design where a damaged or suboptimal microneedle requires replacement of the entire array, impacting overall yield | Modular design allowing for the replacement of individual microneedles without affecting the entire array, enhancing overall yield |
| Reusability | If damage occurs to the microneedle or substrate, the entire structure must be re-fabricated | Reusable microneedle arrays and substrates if undamaged, with only selective replacement of microneedles or electrodes when necessary, lowering cost |
| Application Scenarios | Fixed microneedles and substrate structure, requiring full re-fabrication if application needs change | Independent microneedle and substrate components that can be adapted or replaced for various applications, allowing compatibility with diverse detection scenarios |

**Table S2.** Comparison of this work with microneedle electrodes used for electrophysiological signal monitoring

| Microneedle material | Channel | Single-microneedle accuracy | Microneedle dimensions | Microneedle Spacing | Skin contact impedance | Reconfigurable | Ref. |
| --- | --- | --- | --- | --- | --- | --- | --- |
| Au, Si | 1 | No | 200 μm height | 710 μm | 16.9 kΩ∙cm^2^  (100 Hz) | No | [1] |
| Au, Si | 1 | No | 500 μm height/ 350 µm width | 1.2 mm | - | No | [2] |
| Parylene, silver, SU-8 | 1 | No | 200 μm height/ 400 µm width | 0.43 mm | - | No | [3] |
| PEDOT:PSS,Ti, Au, PI | 1 | No | 400 μm height/ 200 µm width | 500 μm | 1.16 kΩ∙cm^2^  (100 Hz) | No | [4] |
| Si, Ti/Au, Ir, PDMS, Parylene | 25 | Yes | 650 µm height | 800 µm | - | No | [5] |
| PET, Ti/Au,  AgNO3,CMRF | 1 | No | 500 μm height/ 350 µm width | ~1.5 mm | ~50 kΩ∙cm^2^  (100 Hz) | No | [6] |
| Parylene, silver,SU-8 | 1 | No | 230±10 µm height | - | - | No | [7] |
| PEDOT:PSS, Parylene, PDMS, Si | 1 | No | 300 μm  height | 150 μm | 61.2 kΩ·cm^2^  (10 Hz) | No | [8] |
| Cr/Au, Parylene, PI, Ecoflex | 8 | Yes | 500~2000 μm height/ 300~800 µm width | - | - | No | [9] |
| PI, Cr/Au | 1 | No | 800 µm height/ 350 µm width | - | 11.4−21.4  kΩ∙cm^2^ | No | [10] |
| Silicon, Parylene,  Au | 1 | No | 190 µm height | 340 µm | 7.5 kΩ∙cm^2^  (10 Hz) | No | [11] |
| Au, PDMS | 1 | No | 630±46 height / 380±32 µm width | 1.8 mm | - | No | [12] |
| Stainless steel | 1 | No | 657±16 µm height | 1.5 mm | - | No | [13] |
| PEDOT:PSS, Au, Stainless steel | 8 | Yes | 800 µm height/ 200 µm width | 5 mm | 1.48 kΩ∙cm^2^  (100 Hz) | Yes | This work |

**Table S3.** Comparison of this work with microneedle electrodes used for electrochemical signal monitoring

| Microneedle material | Channel | Single-microneedle accuracy | Microneedle dimensions | Analytes  (Sensitivity) | Stability | Reconfigurable | Ref. |
| --- | --- | --- | --- | --- | --- | --- | --- |
| HSM, Carbon, MWCNTs, Stainless steel | 1 | Yes | 500 µm height/ 400 µm width | pH (54.4 mV/pH) | - | No | [14] |
| (GOD), Pt, Au, Stainless steel | 2 | No | 800 µm height/ 400 µm width | Glucose (89.43 μA·mmol^−1^·L^−1^), ROS (35.45 μA·mM^−1^·L^−1^) | 5 days | No | [15] |
| PANI, Au, epoxy siloxane | 25 | Yes | 900 µm height | pH (94 mV/pH) | - | No | [16] |
| ZnO, W | 1 | Yes | 300 µm width | pH (46 mV/pH) | 3 months | No | [17] |
| Graphene , AuNPs, Stainless steel | 1 | Yes | - | pH (29.9 mV/pH) | - | No | [18] |
| PANI, pastes, resins | 4 | No | 1100 µm height/ 1020 µm width | pH (67.2 mV/pH) | - | No | [19] |
| PANI, Au, silane | 1 | No | 250 µm height | pH (62.9 mV/pH) | 20 days | No | [20] |
| PANI, MoS_2_, AN | 1 | Yes | - | pH (51.2 mV/pH) | - | No | [21] |
| CNT, Ag, sMN | 2 | No | 700 µm height/ 250 µm width | UA (7.16 mA·mM^−1^), ROS (16.5 mA·mM^−1^) | - | No | [22] |
| Pt, rGO, Stainless steel | 1 | No | 800 µm height/ 225 µm width | ROS (134 μA·mM^−1^) | - | No | [23] |
| ZnO， Stainless steel | 1 | No | 680 µm height/ 250 µm width | ROS (2.07 mA·mM^–1^·cm^–2^) | - | No | [24] |
| IrOx, Stainless steel | 7 | Yes | 1600 µm height/ 1600 µm width | pH (75.62 mV/pH) | - | No | [25] |
| Pt, MoS2, Au, AN | 1 | Yes | - | ROS (3.73 μA·mM^−1^) | - | No | [26] |
| Carbon, polystyrene | 1 | No | - | pH (57.5 mV/pH) | - | No | [27] |
| Pt, parylene, PGMA | 1 | Yes | 800 µm height | ROS (1.73 μA·mM^−1^) | - | Mo | [28] |
| CNT, PB | 1 | Yes | 1000 μm height | Glucose (16.56 μA·mmol^−1^·cm^−2^), ROS (451 μA·mM^−1^·cm^−2^) | - | No | [29] |
| Pt/PANI, PEDOT:PSS, Au, Stainless steel | 4 | Yes | 800 µm height/ 200 µm width | ROS (435 μA·mM^−1^·cm^−2^),  pH (50.32 mV/pH) | 4 days | Yes | This work |

**Section 2. Design of the RMNEA-integrated system**


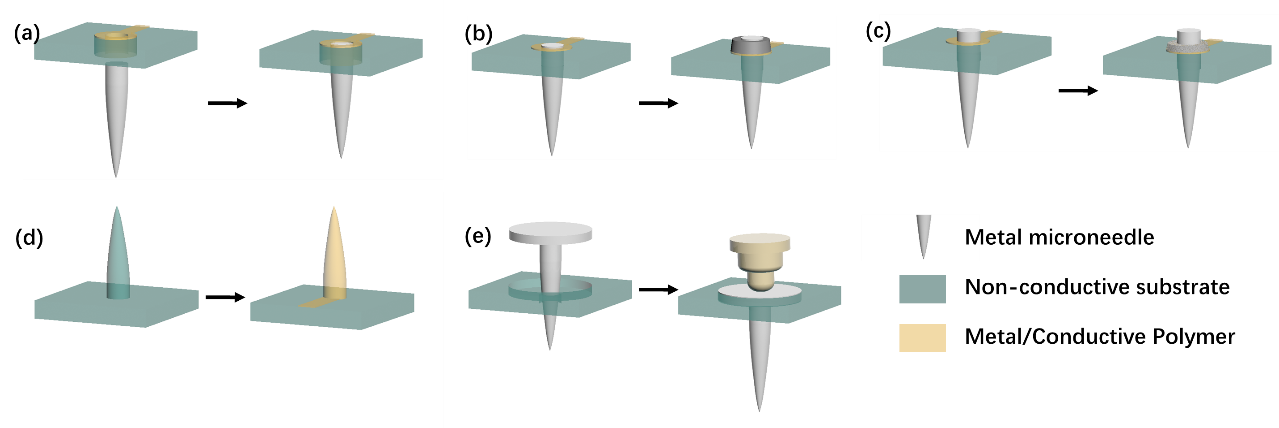


**Figure S1.** Electrical connection methods between single microneedles and substrates. (a) Plug-in connection method. (b) Solder connection method. (c) Silver paste bonding connection method. (d) Deposition connection method. (e) Assembly connection method.

**Table S4.** Comparison between this work and other representative electrical connection methods between single microneedles and substrates

| Connection method | Stability of interface | Reusability of substrate | Replaceable of microneedle | Main Limitation | Ref. |
| --- | --- | --- | --- | --- | --- |
| Plug-in | Medium | Yes | No | - | [30] |
| Solder | Good | No | No | High temperature | [31] |
| Silver paste bonding | Weak | No | No | Long curing time | [32] |
| Deposition | Weak | No | No | High cost | [9, 16] |
| Assembled | Medium | Yes | Yes | - | This work |

**
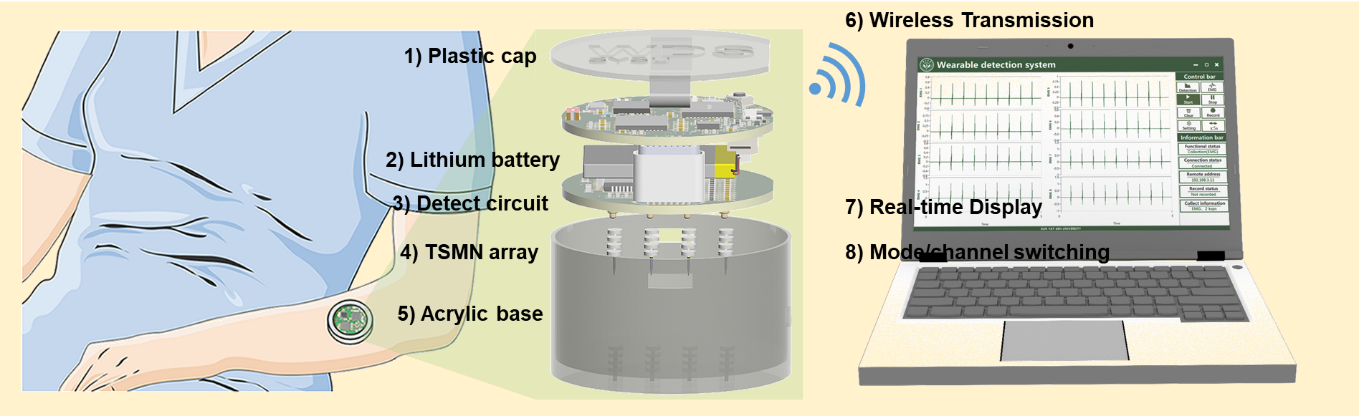
**

**Figure S2.** Components of RMNEA-integrated system.

**
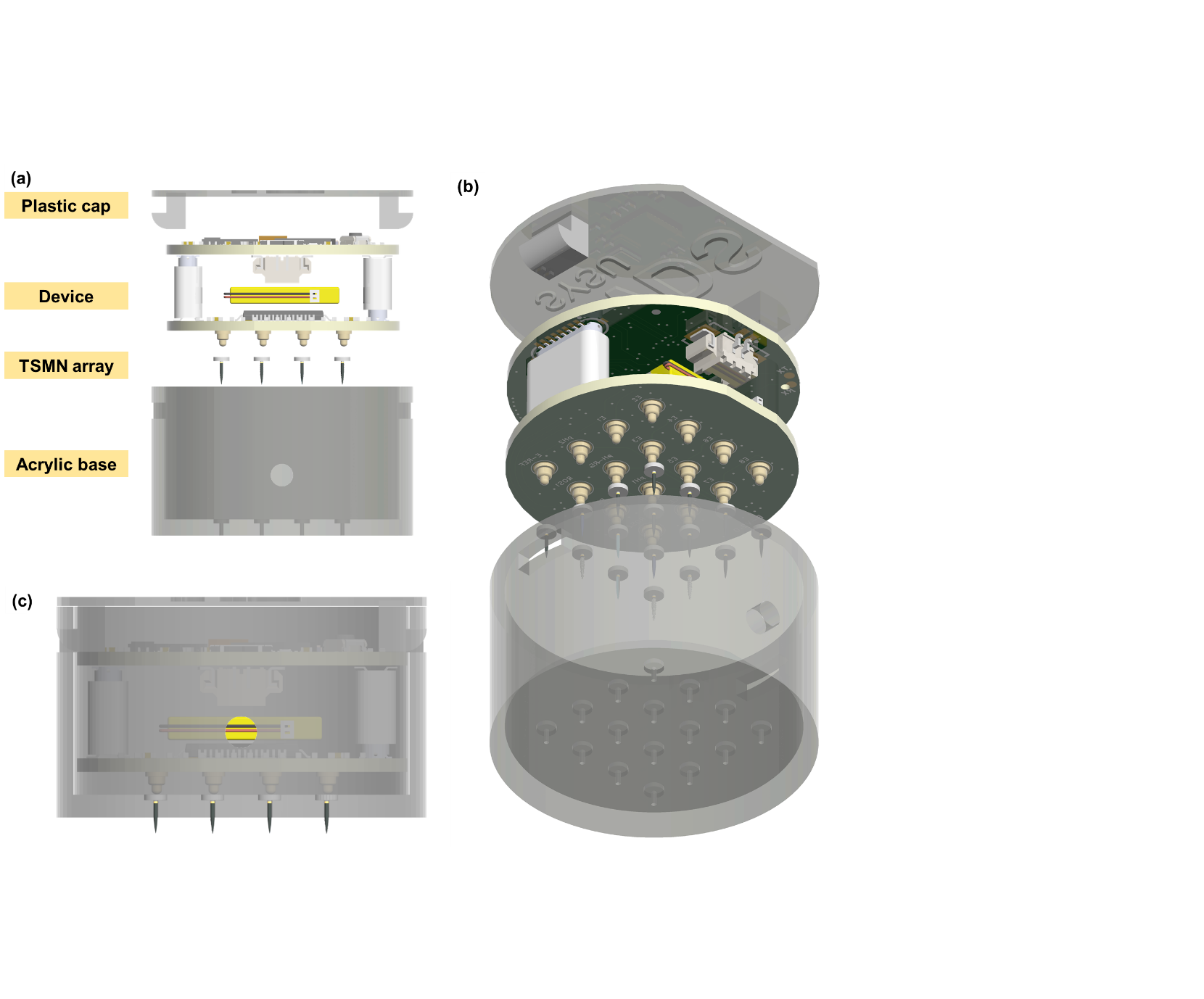
**

**Figure S3.** Schematic diagram of RMNEA-integrated system. (a) Exploded view of the RMNEA-integrated system (side view). (b) Exploded view of the RMNEA-integrated system (bottom view). (c) Side view of RMNEA-integrated system.


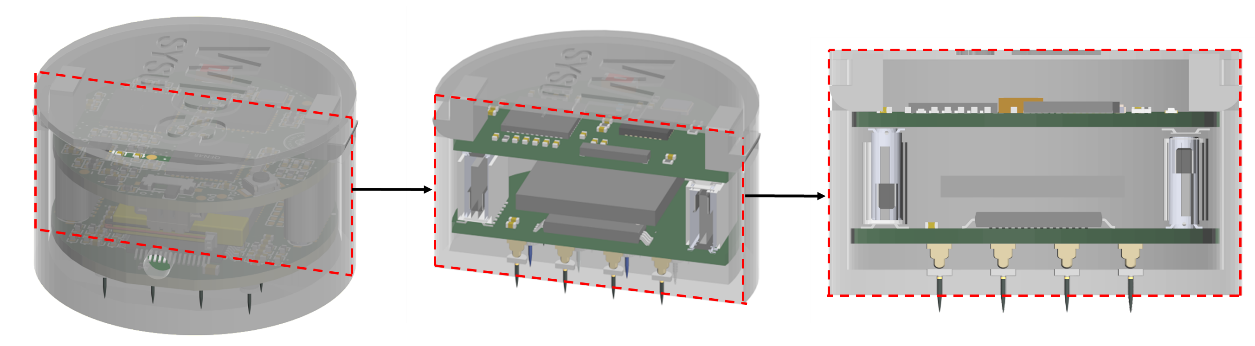


**Figure S4.** Cross section view of RMNEA-integrated system. When the plastic cap is properly engaged with the base, its downward pressure on the underlying detection circuit ensures that the pogo pin exerts continuous force on the TSMNs, thereby establishing a reliable and stable electrical connection.


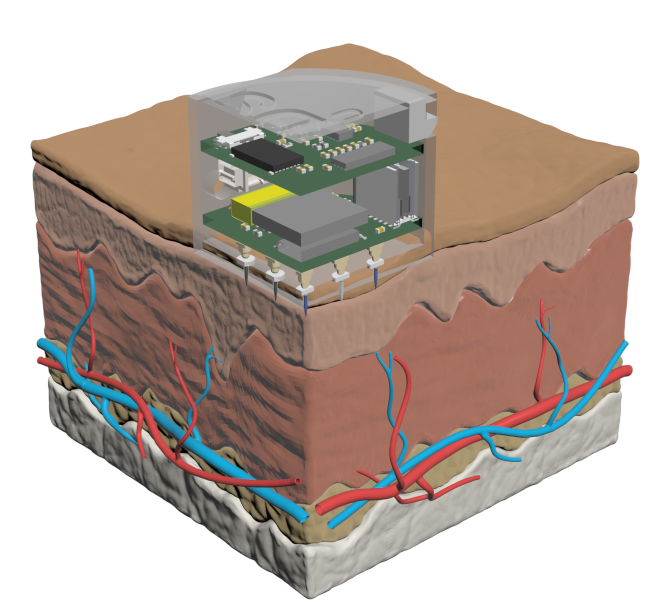


**Figure S5.** Cross sectional view of RMNEA-integrated system/skin interface, where TSMNs penetrate the skin and reach the epidermis.


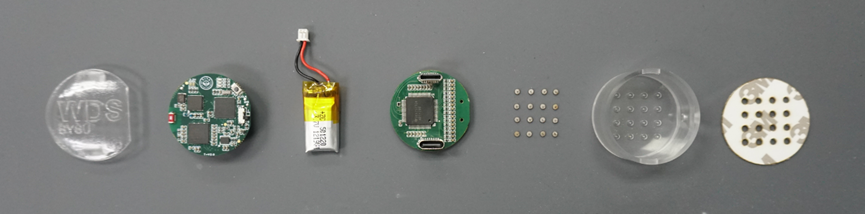


**Figure S6.** Disassembled components of the RMNEA-integrated system. From left to right are plastic cap, upper PCB, battery, lower PCB, 4×4 TSMN array, acrylic base, and double-sided disposable medical tape.


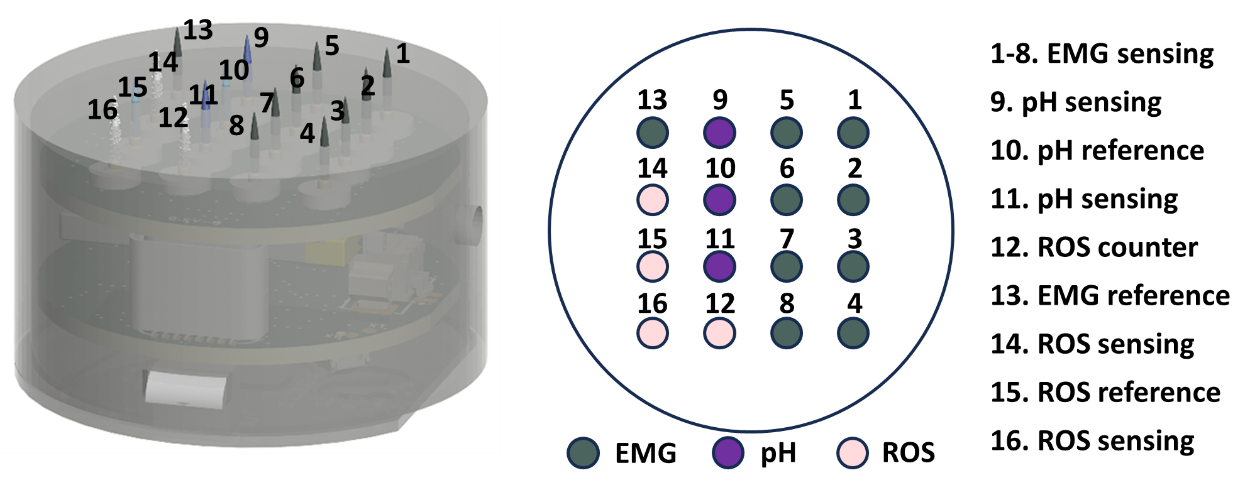


**Figure S7.** Design of the RMNEA-integrated system. In this design, the RMNEA-integrated system contains a total of 16 TSMNs, each of which is individually addressable. The RMNEA-integrated system contains three detection functions (EMG, pH, and ROS).


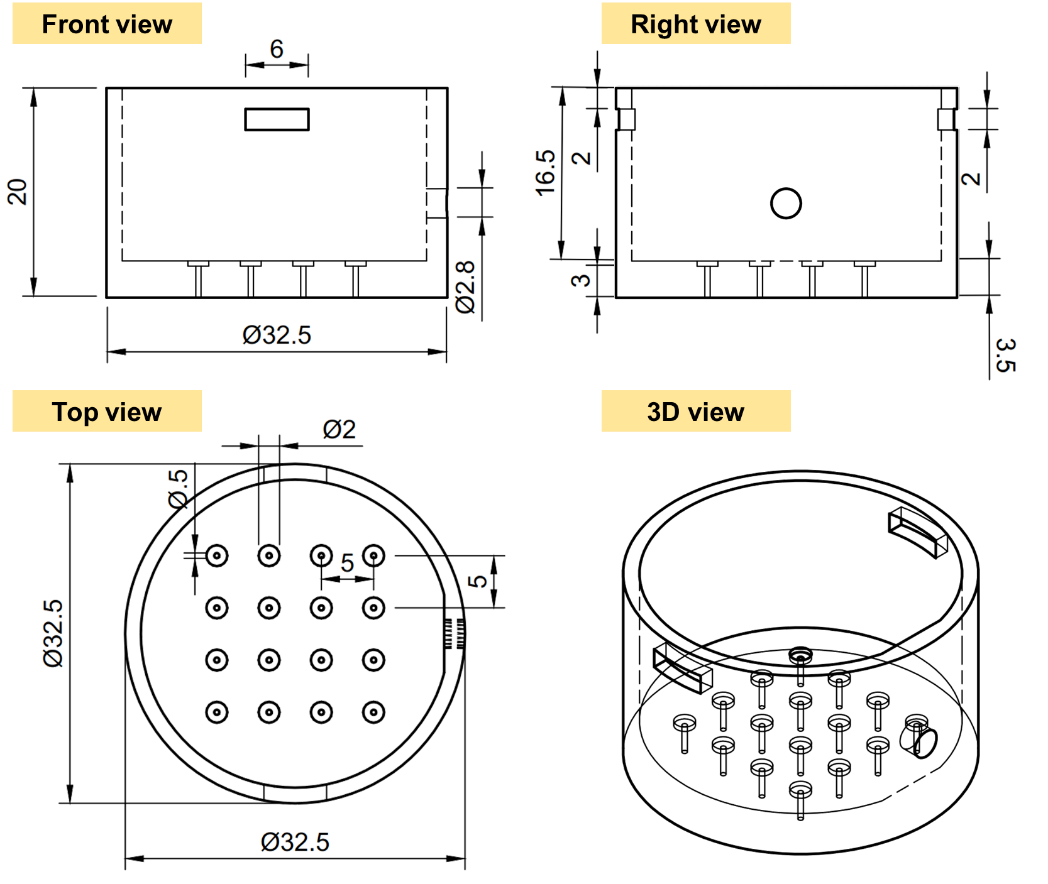


**Figure S8.** Design of the base. Engineering drawings of the actual design dimensions of the base (front view, right view, top view and 3D view). The unit: mm.


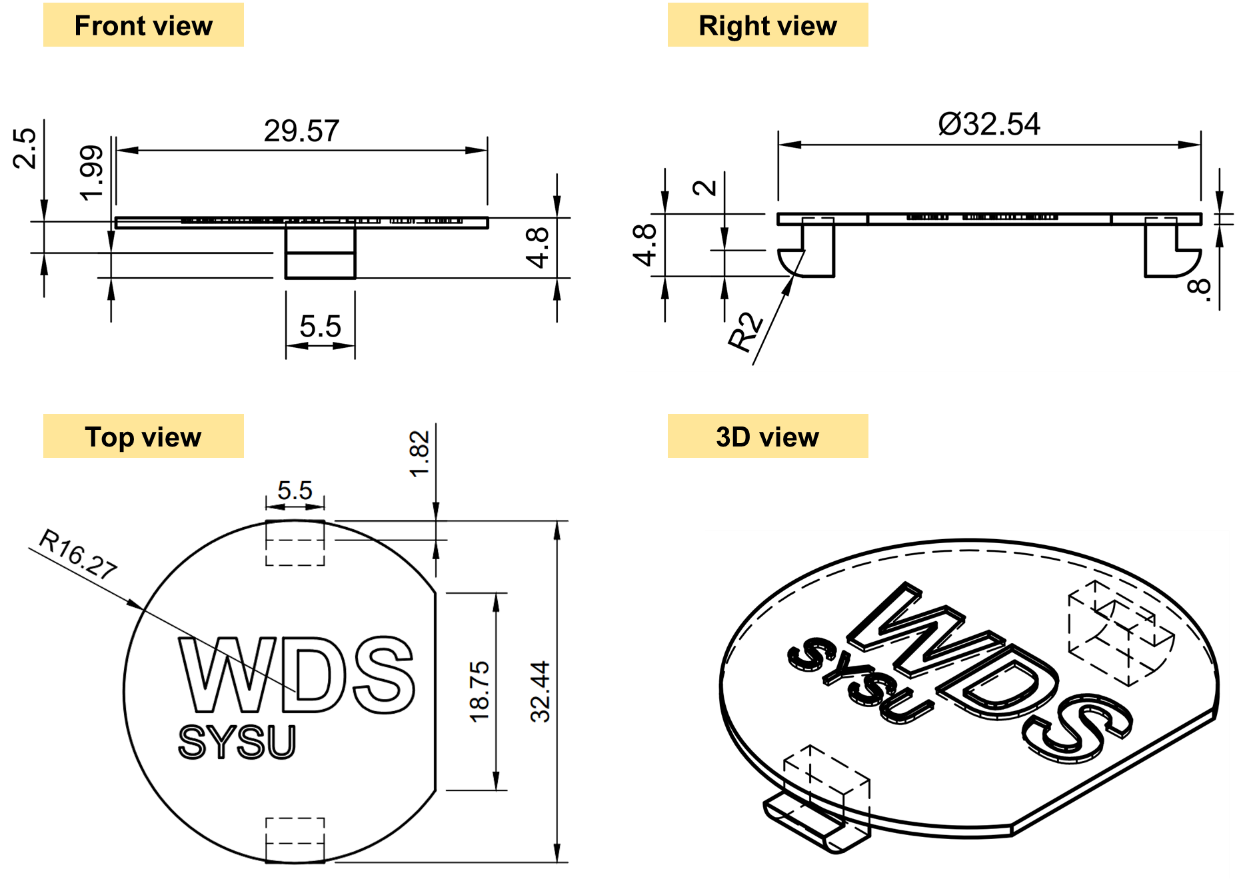


**Figure S9.** Design of the cap. Engineering drawings of the actual design dimensions of the cap (front view, right view, top view, and 3D view). The unit: mm.


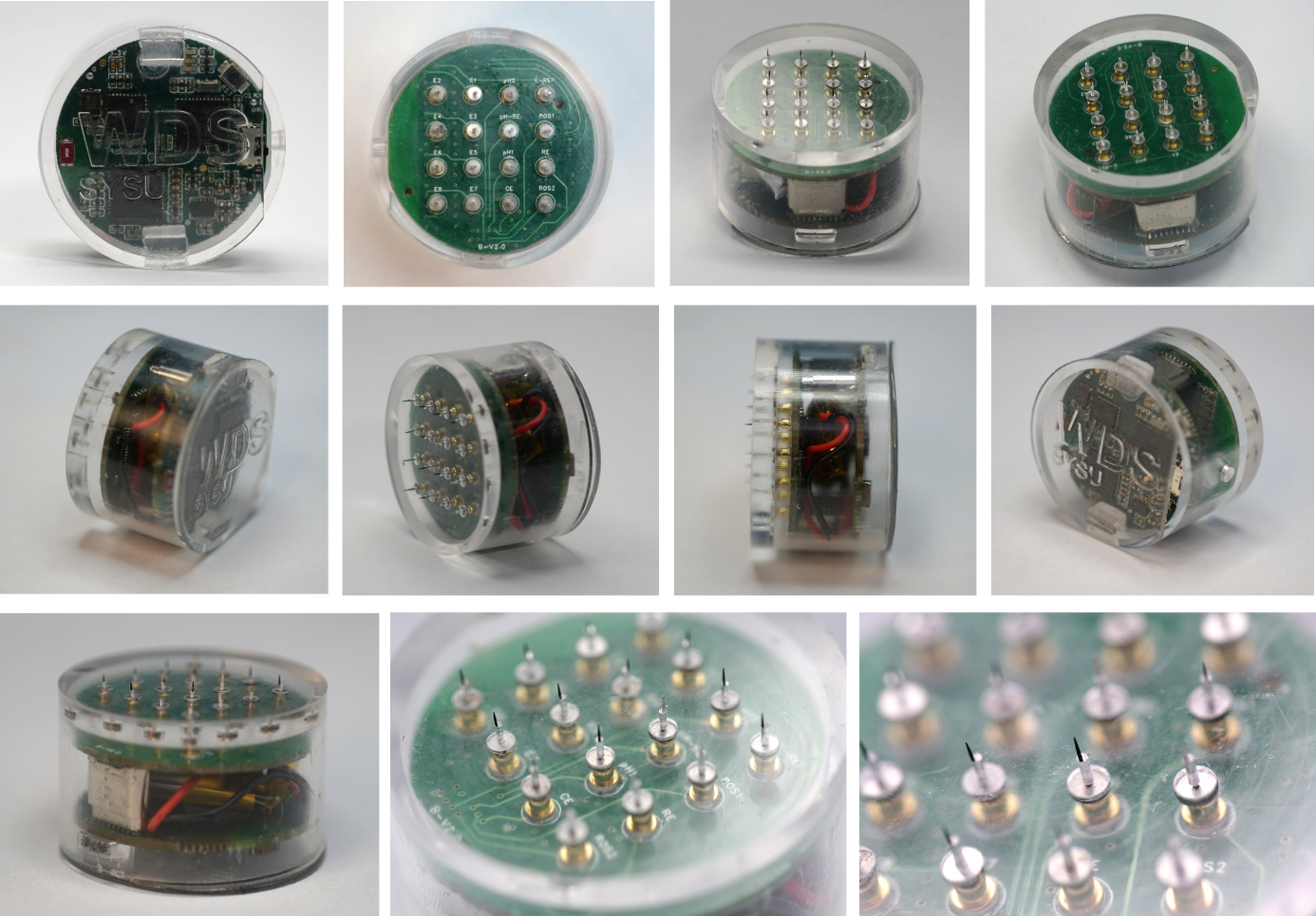


**Figure S10.** Photos of RMNEA integrated system.


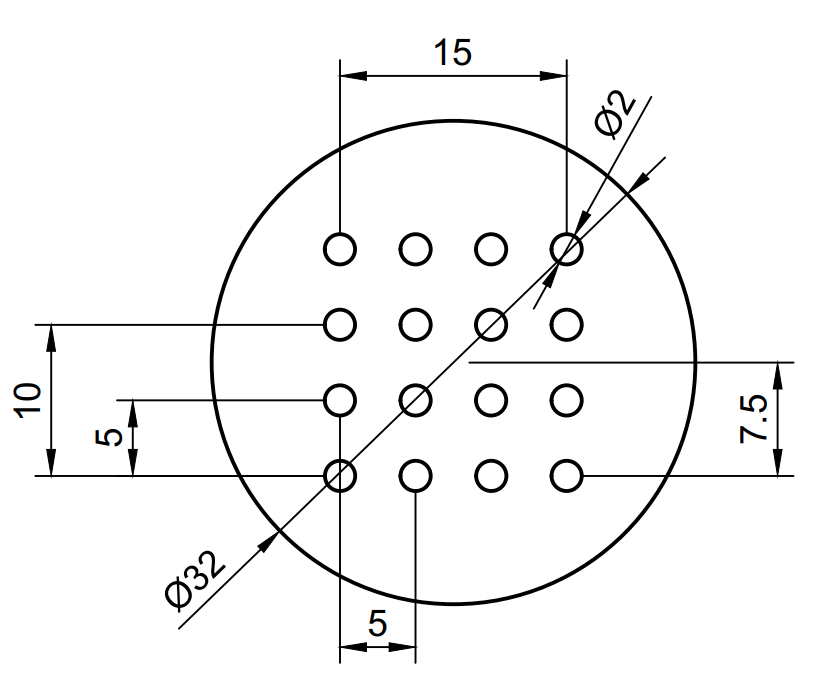


**Figure S11.** Detailed engineering drawing of a double sided medical tape. The unit: mm.

**Section 3. Characterisation and elemental analysis of electrode surface morphology**

**
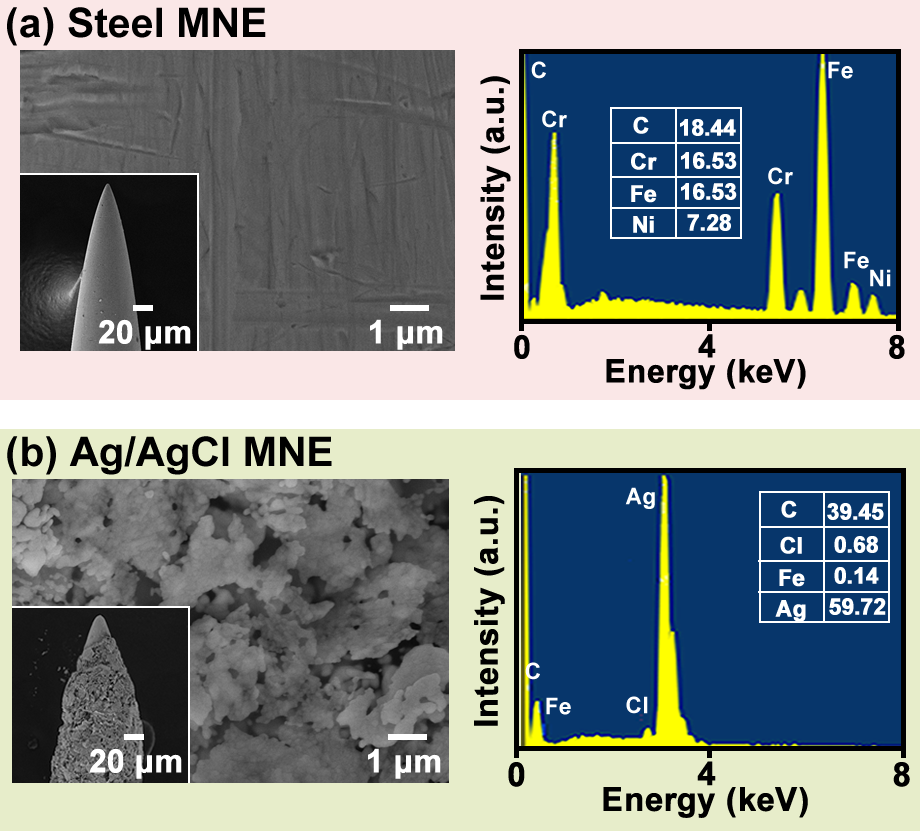
**

**Figure S12.** Morphological characterisation of TSMN. (a) SEM image of the Steel MNE and the corresponding EDX spectrum. (b) SEM image of Ag/AgCl MNE and the corresponding EDX spectrum.

**
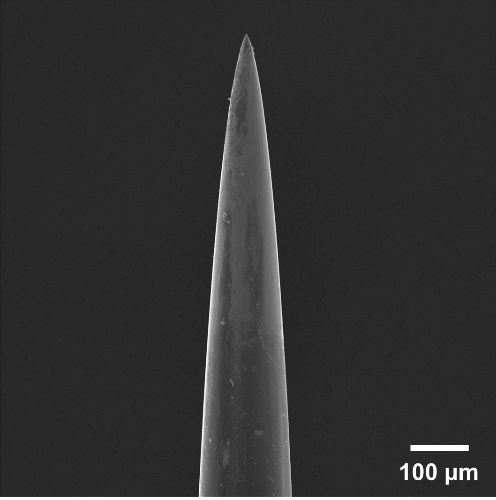
**

**Figure S13.** SEM image of the TSMN beyond the base. The portion of the TSMN beyond the base is conical, with a length of 800 μm.

**Section 4. Simulation of TSMNs penetration through the skin**

**Table S5.** Simulation related physical parameters

|  | Thickness | Density | Frictional coefficient | Young's modulus | Poisson's Ratio |
| --- | --- | --- | --- | --- | --- |
| Stratum corneum | 0.02 mm^[33]^ | 1300 kg/m^3[34]^ | 0.42^[35]^ | 34 kPa^[36]^ | 0.48^[37]^ |
| Dermis | 1.5 mm | 1200 kg/m^3[34]^ | 0.42^[35]^ | 3.4 MPa^[36]^ | 0.48^[37]^ |


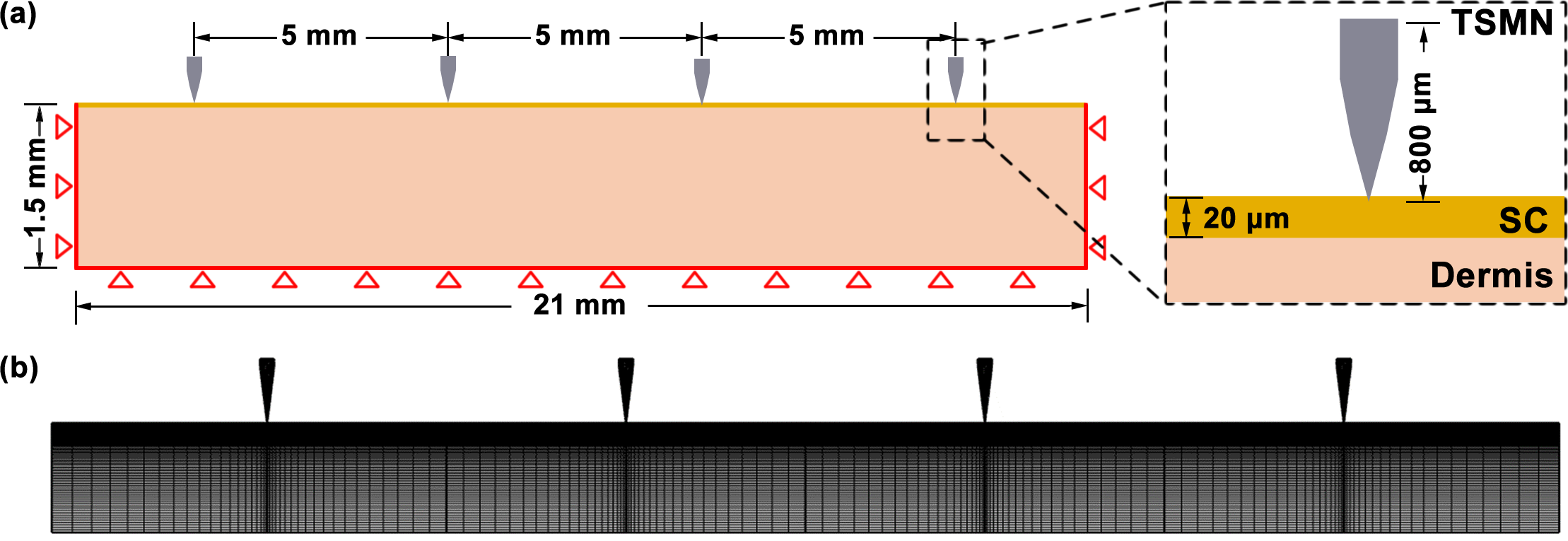


**Figure S14.** Finite element model of TSMN penetration and retraction in the skin. (a) Detailed dimensional parameters of the simulation model. (b) A finite element model of penetration and retraction of TSMNs in the skin.


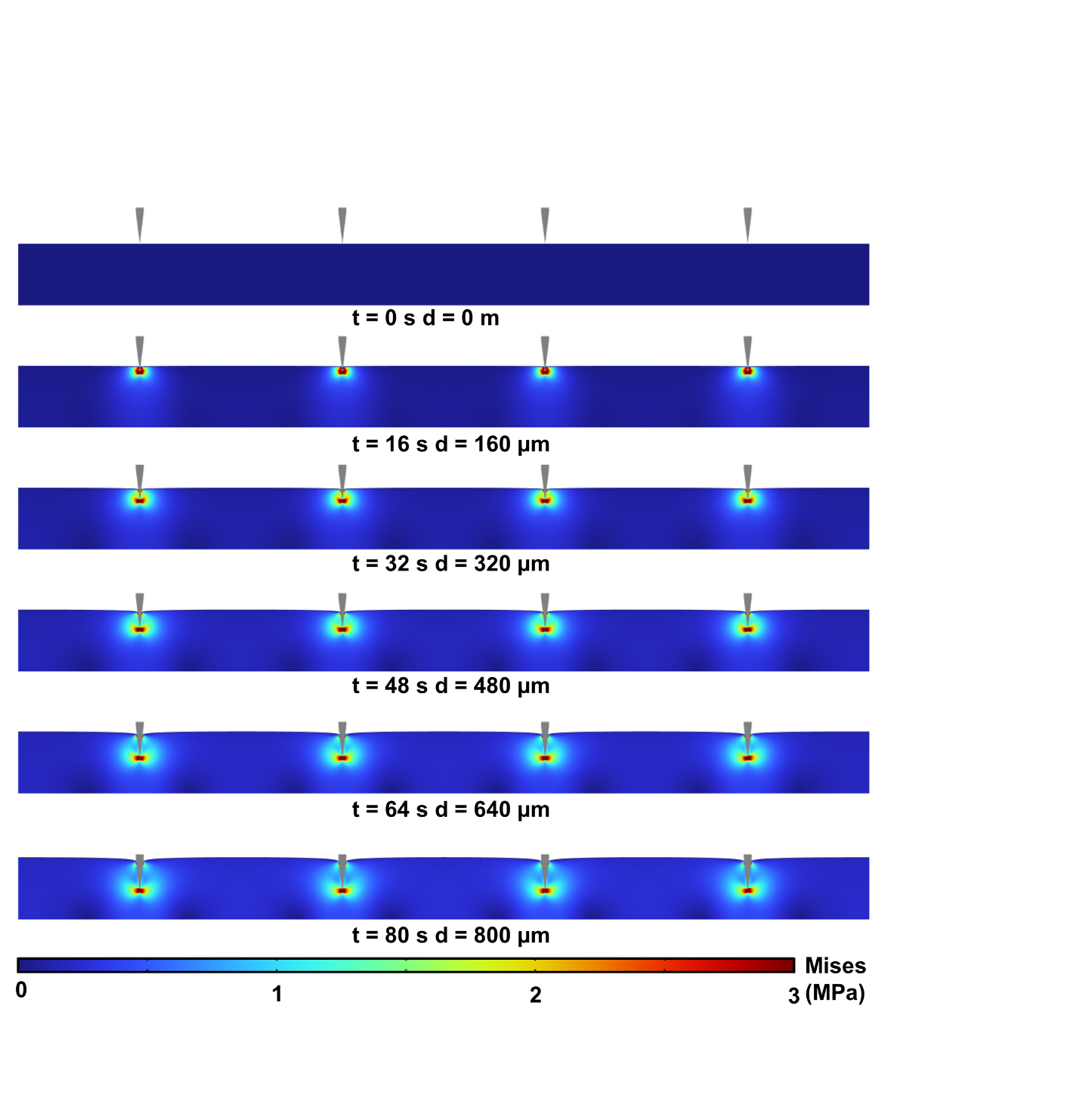


**Figure S15.** The process of inserting TSMNs into the skin (time: 80 seconds).


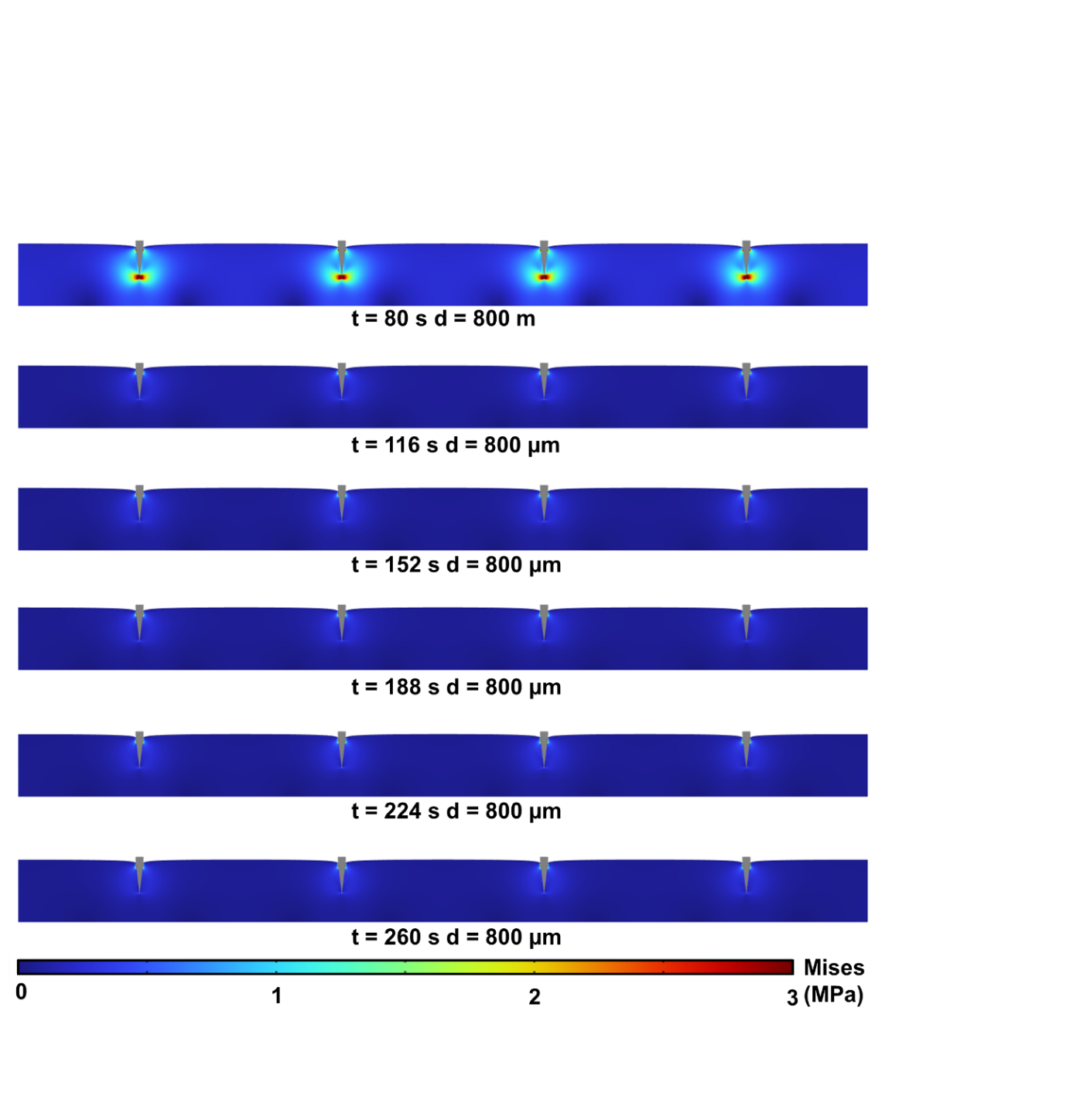


**Figure S16.** The process of TSMNs resting inside the skin (duration: 180 seconds).


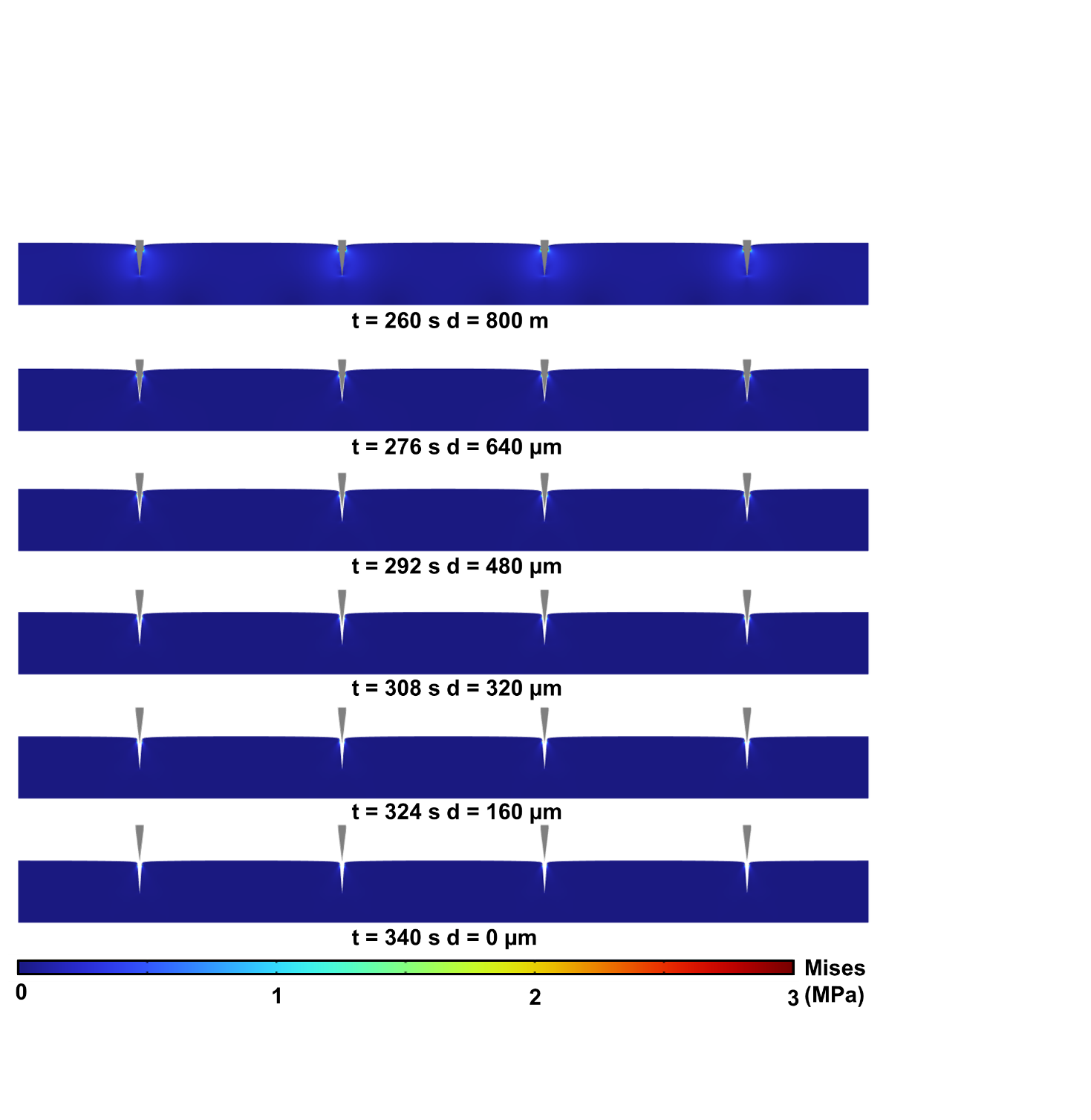


**Figure S17.** The process of removing TSMNs from the skin (duration: 80 seconds).

**Section 5. Mechanical testing of TSMNs penetration into the skin**


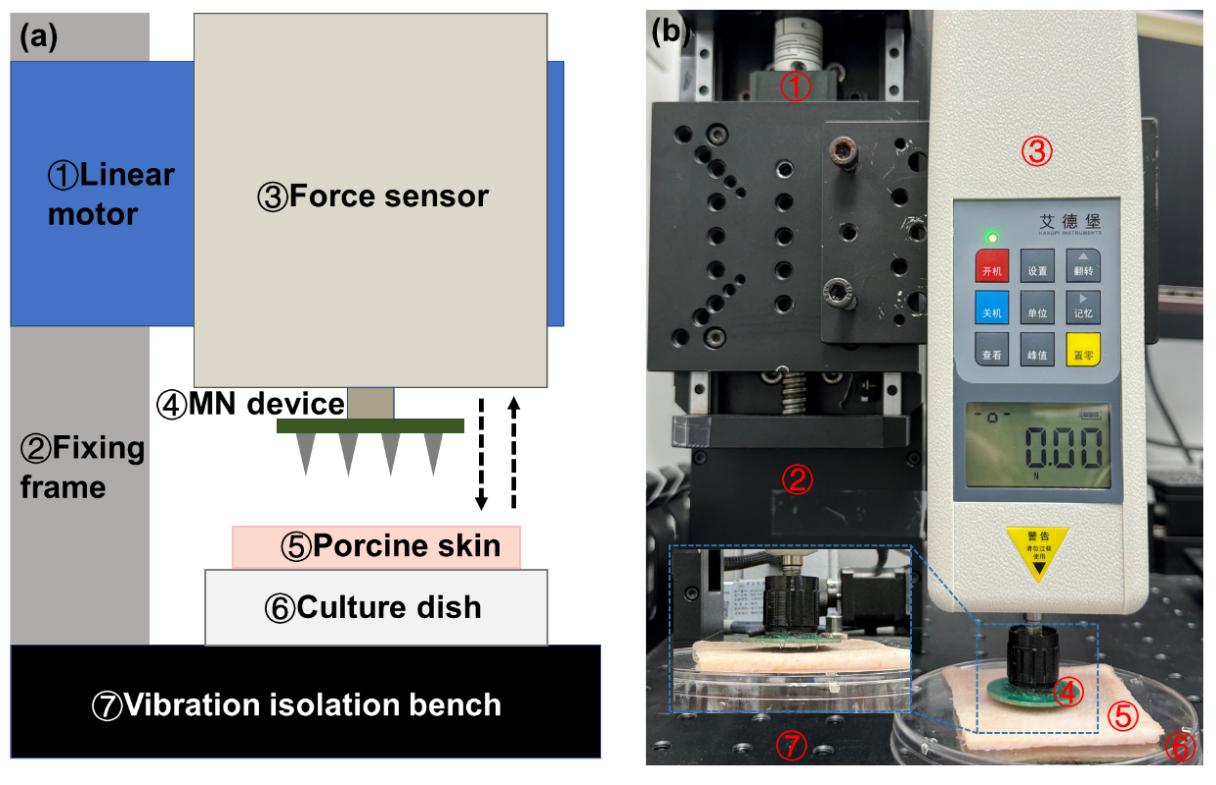


**Figure S18.** Schematic diagram of the RMNEA insertion-removal skin mechanics testing device. The RMNEA was first fixed to the test bar of the HP-20 dynamometer. Next, a fresh pork skin sample was selected and placed on the platform below the dynamometer. Subsequently, the force gauge was moved vertically downwards at a constant speed of 0.2 mm/s using a GCD-203050M electric moving stage to enable the RMNEA to be inserted smoothly and vertically into the pigskin sample. Once the microneedles were fully inserted, the movement of the displacement stage was immediately stopped and the TSMNs remained stationary in the pigskin for up to 3 seconds. After the waiting time, the electric moving stage was activated to vertically pull the RMNEA out of the pigskin at the same speed as during insertion.


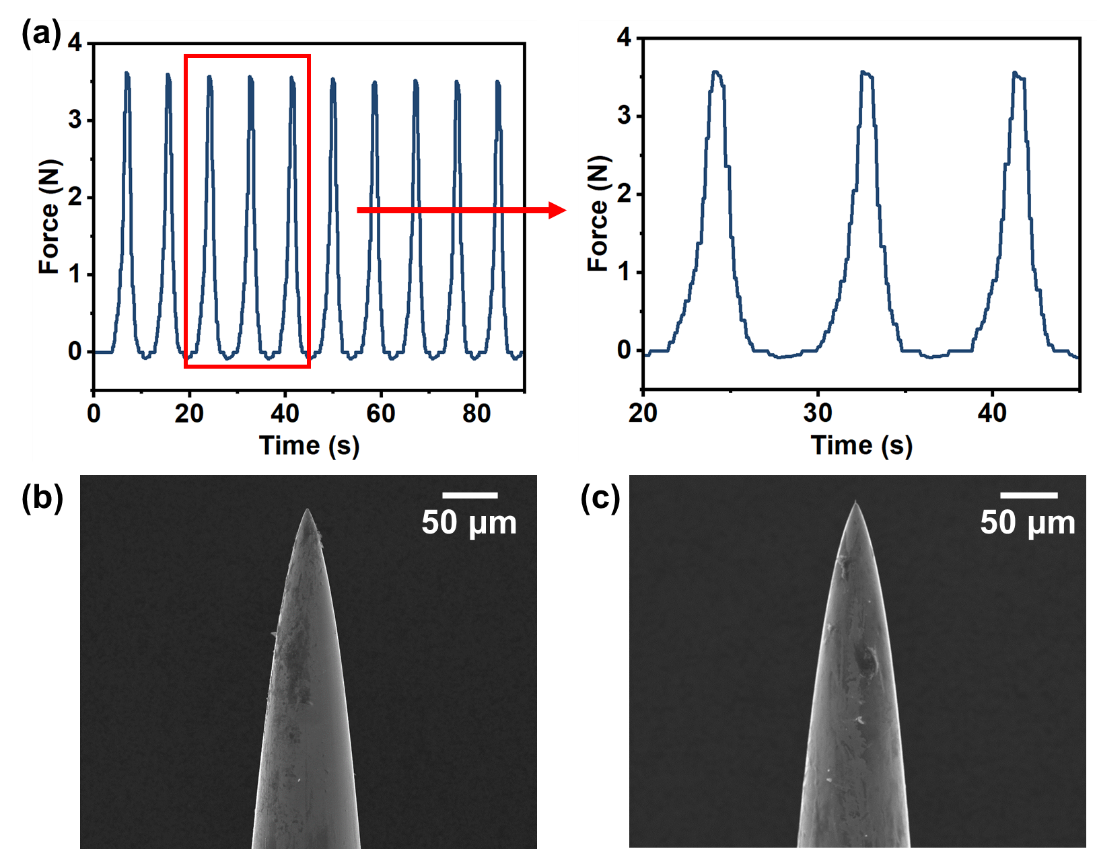


**Figure S19.** RMNEA repeated insertion and removal skin test. (a)Time-strain graph for repeatedly inserting and removing the RMNEA 10 times on pig skin. (b) SEM image of the magnified RMNEA before repeated skin insertion and removal. (c) SEM image of the magnified RMNEA after the 10th cycle of skin insertion and removal.

**Section 6. Electrochemical characterization of sensing electrodes**

**Note S5.** Discussion on the advantages of PEDOT:PSS coating in sensor applications in this article.

PEDOT:PSS coatings have several significant advantages in sensor applications. Firstly, PEDOT:PSS is a stable coating materials, that allows us to fabricate it on single microneedle electrode with high robustness. The coating of PEDOT:PSS through electroplating is comparable with our reconfigurable design of microneedle electrode array and high-throughput fabrication process. Additionally, PEDOT:PSS dramatically reduces the electrode impedance, which effectively improves the signal-to-noise ratio, resulting in higher sensitivity in both electrophysiological and electrochemical detection. By providing greater capacitance, PEDOT:PSS enables the electrode to store more charge during electrochemical processes, improving signal transmission efficiency and response speed. This high charge storage capacity ensures superior electrode stability, especially during rapid electrochemical reactions.

**Table S6.** Comparison of this work with modified coatings on electrodes

| Modified Coating | Modification method | Reduction of Impedance (%) | Enhancement of CSC | Stability | Detection | Ref. |
| --- | --- | --- | --- | --- | --- | --- |
| Gold nanosheet | Drop Coating | 81 % (1 kHz) | - | 6 months | LFP | [38] |
| Pt-black | Electrodeposition | 77 % (1 kHz) | 5.2 | 3 months | ECoG | [39] |
| PEDOT/MWCNT | Electrodeposition | 38 % (1 kHz) | 4.9 | - | ECoG | [40] |
| PEDOT:PSS | Electrodeposition | 92 % (100 Hz) | 251 | - | EMG, EEG | [41] |
| SWCNTs/PEDOT:PSS | Electrodeposition | 97 % (1 kHz) | - | - | LFP, DA | [42] |
| GO/PEDO:PSS | Electrodeposition | 93 % (100 Hz) | 28.5 | - | DA | [43] |
| rGO-PEDOT：PSS/Nafion | Electrodeposition, Drop Coating | 99 % (100 Hz) | 103 | - | DA, 5-HT | [44] |
| Pt-grass | Electrodeposition | ~ 95 % (100 Hz) | ~ 40 | - | - | [45] |
| Graphene | Polymer-free transfer | 20 % (1 kHz) | 1.2 | - | - | [46] |
| IrOx/Pt | Electrodeposition | 93 % (1 kHz) | 7.1 | - | LFP | [47] |
| IrOx | Electrodeposition | 20 % (1 kHz) | 30 | - | - | [48] |
| Nanozyme | Electrodeposition | ~ 88 % (1 kHz) | 26 | 1 month | LFP | [49] |
| Porous Pt | Sputtering deposition | ~ 89 % (1 kHz) | 1.6 | - | LFP | [50] |
| Pt nanoparticles | Spark ablation | 77% (1 kHz) | 15 | - | - | [51] |
| PEDOT/CNT | Electrodeposition | 98 % (1 kHz) | 65 | - | EMG | [52] |
| CNT | TCVD | 90 % (1 kHz) | ~ 6 | - | - | [53] |
| PEDOT:PSS | Electrodeposition | 98 % (100 Hz) | 38.4 | 4 day | EMG, ROS, pH | This work |


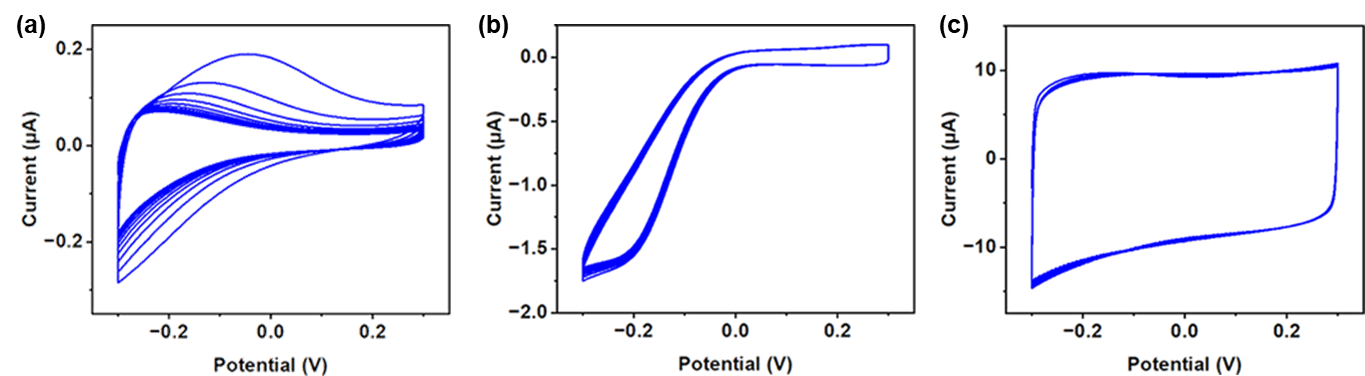


**Figure S20.** The electrochemical stability of the (a)Steel MNE, (b) Au MNE, (c) PEDOT:PSS/Au MNE upon 10 CV cycles. The CV curves were recorded at a scan rate of 100 mV/s in a mixed solution containing 5 mM potassium ferricyanide and 0.1 mM KCl.

**
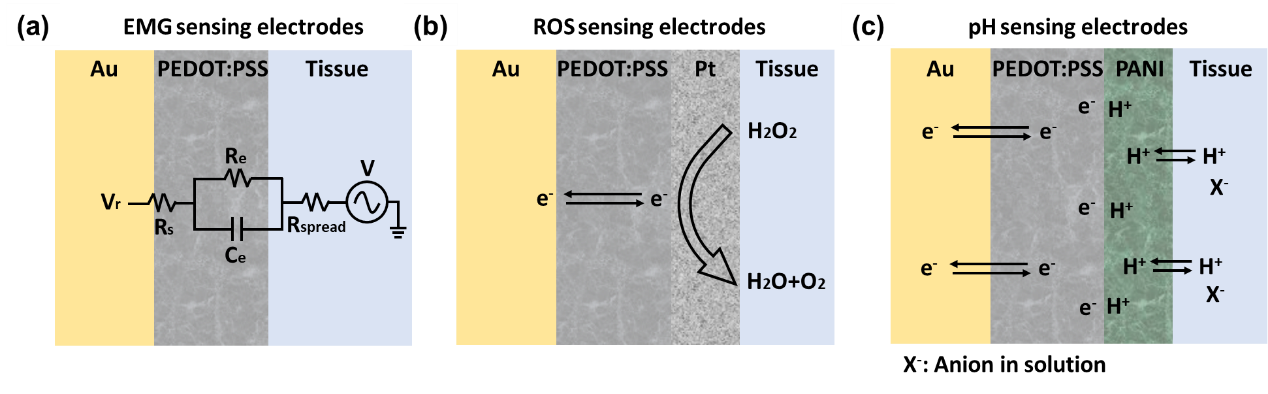
**

**Figure S21.** PEDOT: PSS was used as an electronic medium on various sensing TSMNs to enhance the sensitivity of the sensors. (a) PEDOT:PSS enhanced the electron transfer at the electrode surface, which reduced the interfacial impedance of the electrode and improved the quality of the EMG signal^[54]^. (b) PEDOT:PSS increased the area of the ROS sensing reaction and served as a medium for converting ion flow into electron flow, thereby improving the sensitivity of the ROS sensor. (c) When hydrogen ions entered the ion selective membrane (ISM) and caused a change in potential, PEDOT:PSS was able to efficiently transport electrons and quickly reflected the potential change, ensuring a sensitive response of the electrode to pH changes.


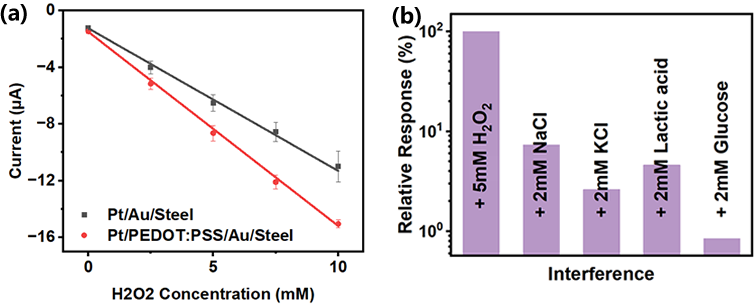


**Figure S22.** In vitro electrochemical performance characterization of ROS sensors. (a) Comparison of ROS current response between Pt/Au electrode and Pt/PEDOT:PSS/Au MNE, N=3 groups. (b) Pt/PEDOT:PSS/Au MNE selectivity diagram for H_2_O_2_ sensing. Test the selectivity of electrode detection by sequentially adding 2 mM NaCl, 2 mM KCl, 2 mM Lactic acid, or 2 mM Glucose to the test solution of the electrode. For better discussion, consider the first response signal as 100%, and then normalize the subsequent signals and convert them to relative values.


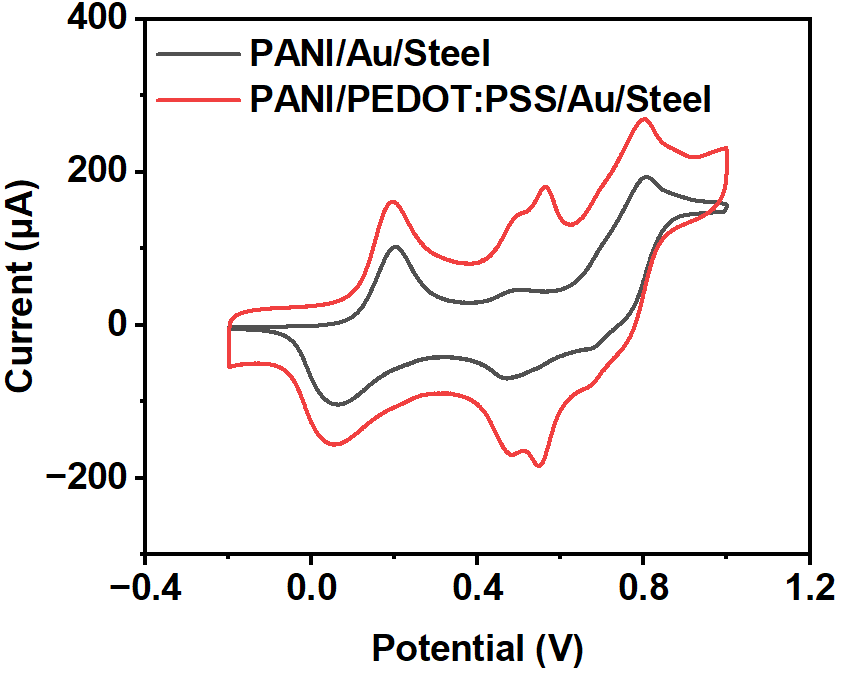


**Figure S23.** A CV curve of PANI/Au/Steel MNEs and PANI/PEDOT:PSS/Au/Steel MNEs recorded in H_2_SO_4_ (0.5 M) at potentials ranging from -0.2 V to 1 V at scan rate of 100 mV s⁻¹.


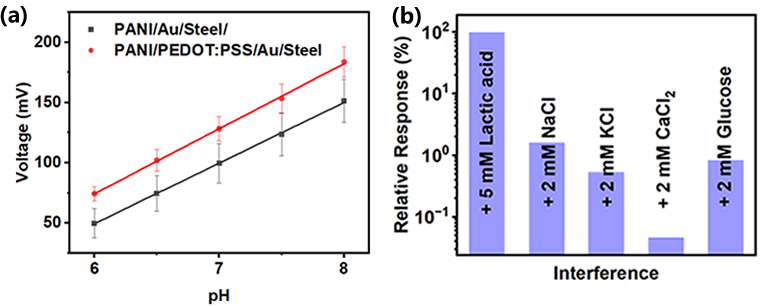


**Figure S24.** In vitro electrochemical performance characterization of pH sensors. (a) Comparison of voltage response between PANI/Au MNE and PANI/PEDOT:PSS/Au MNE, N=3 groups. (b) Pt/PEDOT:PSS/Au MNE selectivity diagram for pH sensing. Test the selectivity of electrode detection by sequentially adding 2 mM NaCl, 2 mM KCl, 2 mM CaCl_2_, or 2 mM Glucose to the test solution of the electrode. For better discussion, consider the first response signal as 100%, and then normalize the subsequent signals and convert them to relative values.

**Section 7. Design and development of the detection device**

**Table S7.** Technical specifications of detection device

| Feature | Configuration |
| --- | --- |
| EMG | |
| Channels | 8 |
| Input mode | Differential |
| PGA | 24 |
| Supply mode | Bipolar ± 2.5 V |
| Data rate | 2000 SPS |
| pH | |
| Channels | 2 |
| Input mode | 2-electrode |
| PGA | 9 |
| Data rate | 1 SPS |
| ROS | |
| Channels | 2 |
| Input mode | 3-electrode |
| R_TIA_ | 160 kΩ |
| Data rate | 1 SPS |

**Table S8.** Comparison between this work and other representative electrophysiological/electrochemical detection circuit.

| Number of  channels | Sampling Rate (Hz) | ADC Resolution | Battery (mAH) | PCB Size (cm^2^) | Wireless Technology | Ref. |
| --- | --- | --- | --- | --- | --- | --- |
| ECG(1), Glucose(1) | - | ECG(24 bits) | 220 | - | BLE | [55] |
| ECG(1), Glucose(1) | - | ECG(24 bits) | - | 10.62 | BLE | [56] |
| ECG(1), Impedance(1), pH(1), Lactate(1) | ECG (140 Hz) | ECG/Impedance/pH Lactate (10 bits) | 80 | 4.08 | BLE | [57] |
| EMG (8) | EMG (400 Hz) | EMG (12 bits) | - | 15 | BLE | [58] |
| EMG (8) | EMG (1 kHz) | EMG (24 bits) | 400 | 42.5 | BLE | [59] |
| EMG (4) | EMG (1.6 kHz) | EMG (12 bits) | - | 8.5 | Custom 2.4 GHz | [60] |
| EMG (8) | EMG (1 kHz) | EMG (24 bits) | - | - | BLE | [61] |
| ROS (1), Glucose (1) | - | ROS/Glucose(12 bits) | - | 22.9 | BLE | [62] |
| ROS (1), Glucose (1), UA(1) | - | ROS/Glucose UA /(12 bits) | - | 22.4 | BLE | [63] |
| pH(1), Na (1), K(1) , Ca(1),Uric acid(1) | - | pH/Na/K/Ca/Uric acid(12 bits) | 2000 | - | BLE | [64] |
| EMG (8), ROS (2), pH (2) | EMG (2 kHz), ROS (1 Hz), pH (1 Hz) | EMG (24 bits), ROS /pH (16 bits) | 150 | 6.6 | Wi-Fi | This work |


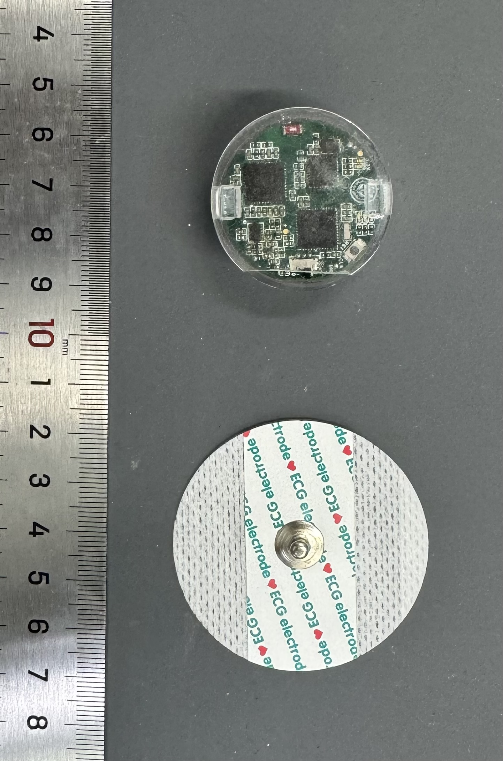


**Figure S25.** Comparison of size between device and commercial Ag/AgCl electrode (LT-302, Shanghai Litu Medical Devices Co., Ltd.).


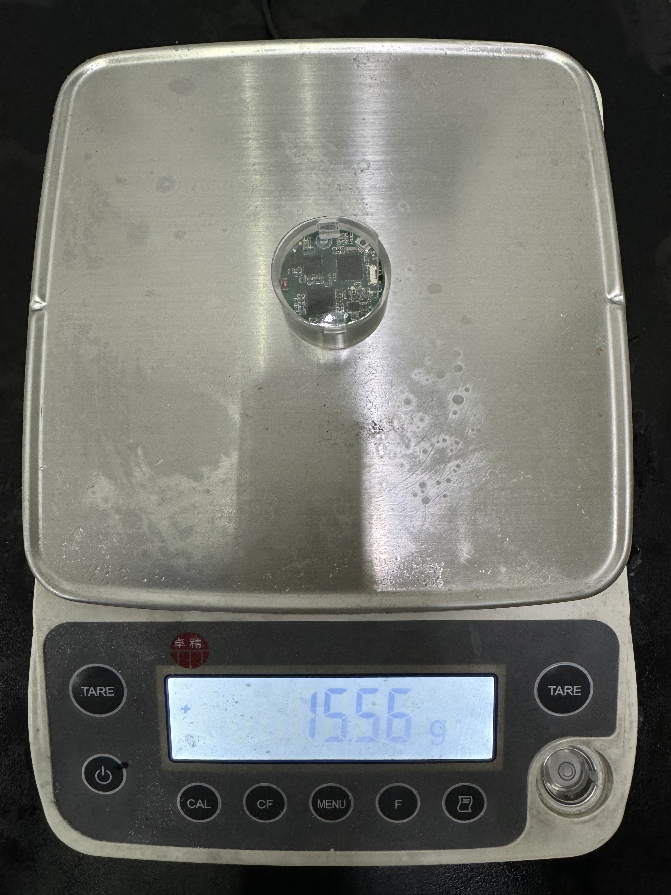


**Figure S26.** Weight measurement of the detection device.


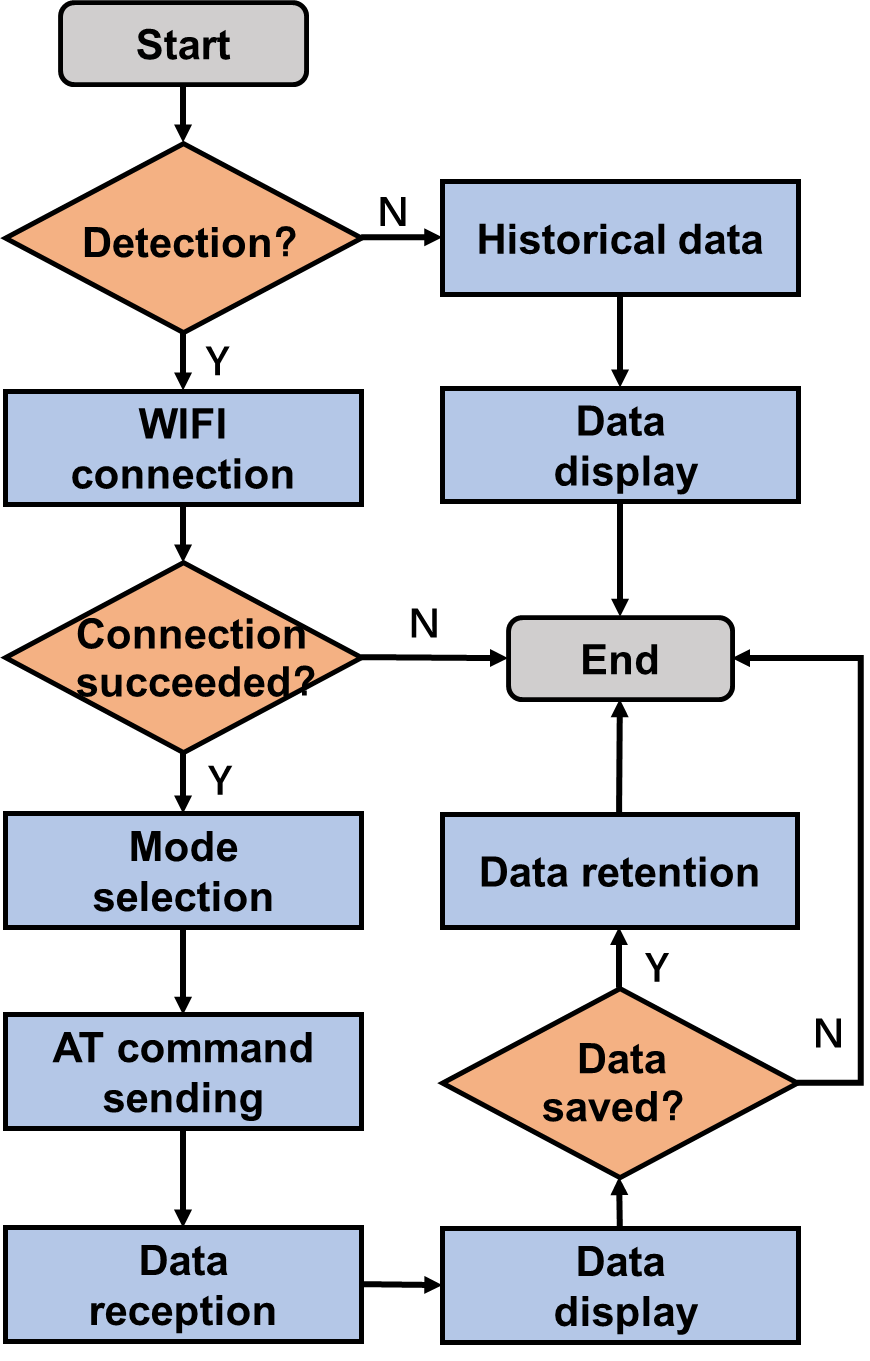


**Figure S27.** Flowchart of the acquisition software. First, the user first selects detection or playback. When the selection mode is detection, after connecting to the detection device through Wi-Fi. After successful connection, when the detection type is selected, control commands will be sent to the detection device and the detection data returned from detection device will be received and dynamic curves will be established and displayed. The historical detection data can be saved according to whether it is needed to be saved or not. When selecting the mode as playback, the corresponding file is selected for playback and displayed as a dynamic curve.


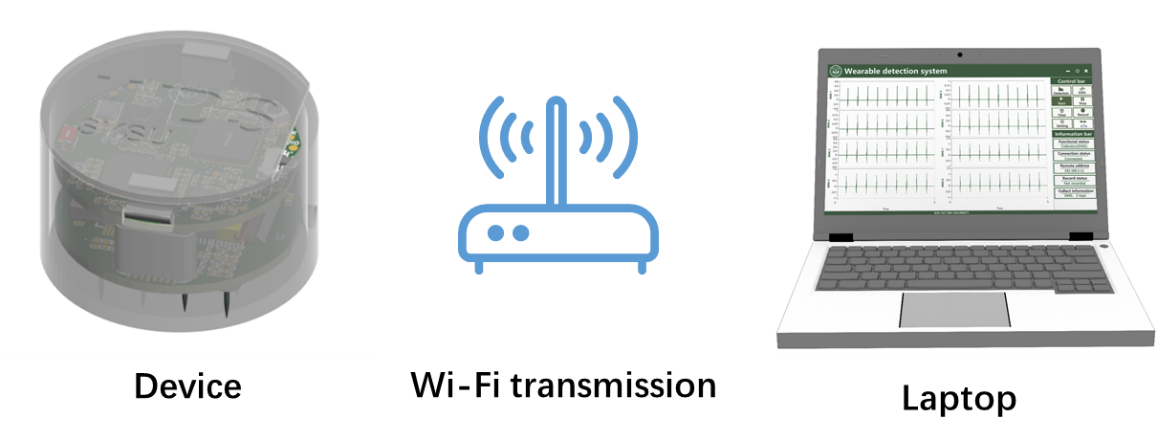


**Figure S28.** Diagram of the wireless communication between the device and the laptop. The Wi-Fi module of the detection device is configured in STA mode, allowing it to connect to routers in the local area network as a client. At the same time, the computer is also connected to the same router through Wi-Fi, ensuring that the computer and the detection device are in the same local area network. The Wi-Fi module of the detection device will package the collected EMG signals and electrochemical data into data packets through TCP protocol and send them to the router. The router will forward these packets to the connected computer. The computer receives data packets from the detection device, decodes and displays them, and finally displays the detected physiological signals in real time.


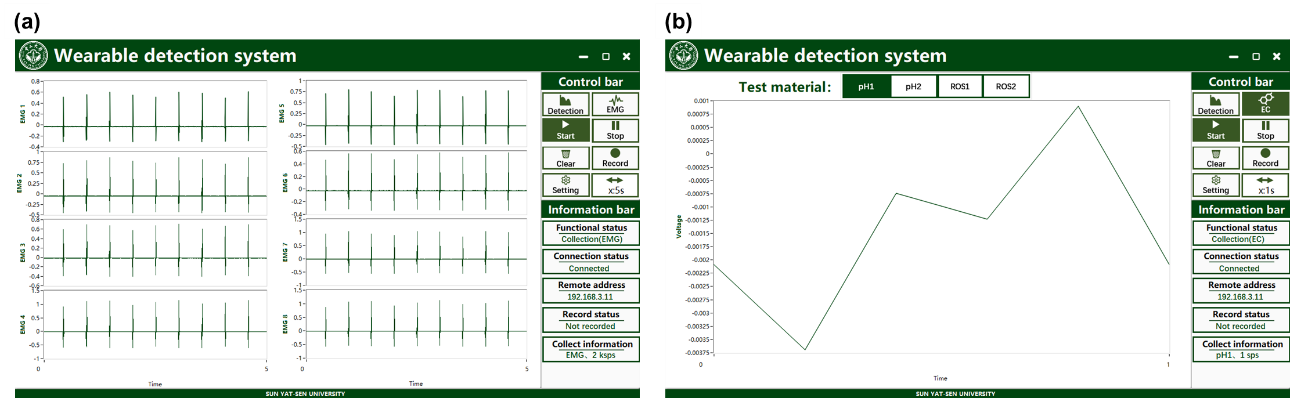


**Figure S29.** Software user interface. (a) When the detected signal is EMG, the waveform display area shows the waveform graphs of eight channels. The user interface is divided into five parts: title bar, control bar, information bar, waveform display area, and copyright information area, which can provide a clear user interface and enable users to conveniently control and monitor the physiological signals of the wearable device. The control bar includes "Function", "Mode", "Start", "Stop", "Clear", "Record", "Settings" and "X-axis range". "Function" is used to select whether the software is used for acquisition or playback. "Mode" is used to select whether the currently detected signal is EMG or electrochemical. The "Start" and "Stop" buttons are used to control the start and stop of physiological signal recording. "Clear" deletes the data currently displayed in the waveform. The "Record" button is used to save the acquired physiological signal data as a TDMS file, and the saving of physiological signal data is terminated when the "Stop" button is clicked. "Settings" is used for network configuration options, setting the network protocol and port settings for pairing and connecting the wearable device. "X-axis range" is used to set the horizontal scale range of the waveform graph. (b) When the detected signal is electrochemical, the waveform display area shows the waveform graph of one channel.


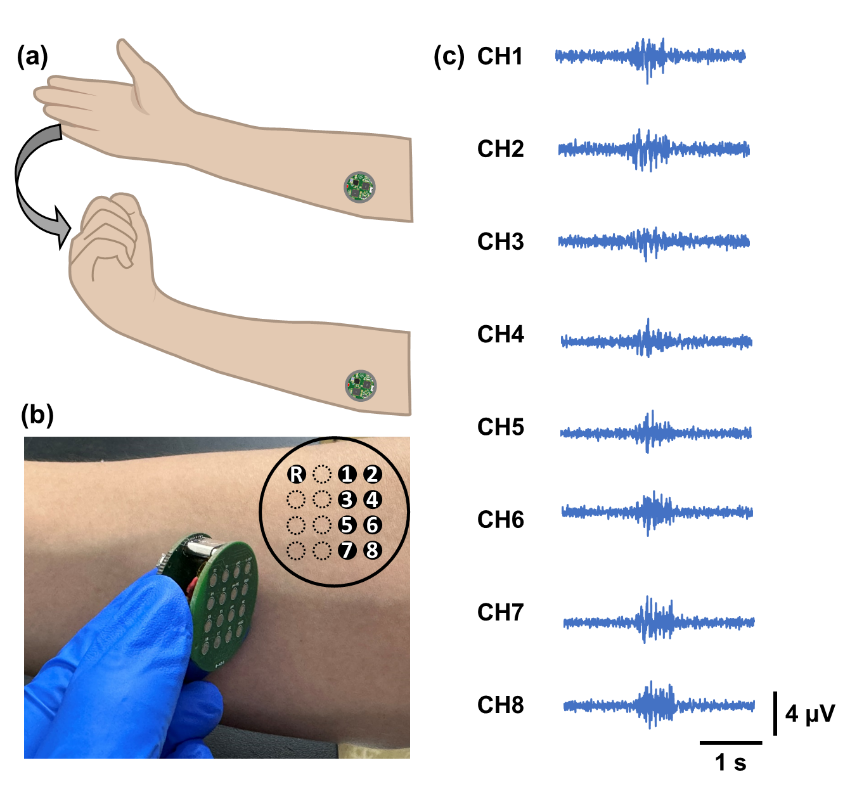


**Figure S30.** Functional assessment of EMG detection. (a) Acquisition of EMG signals from a fist-clenching movement. (b) The detection device without pogo pin connectors was pressed onto the arm and the electrodes were placed on the forearm to record EMG. (c) Raw signals recorded during the movement.


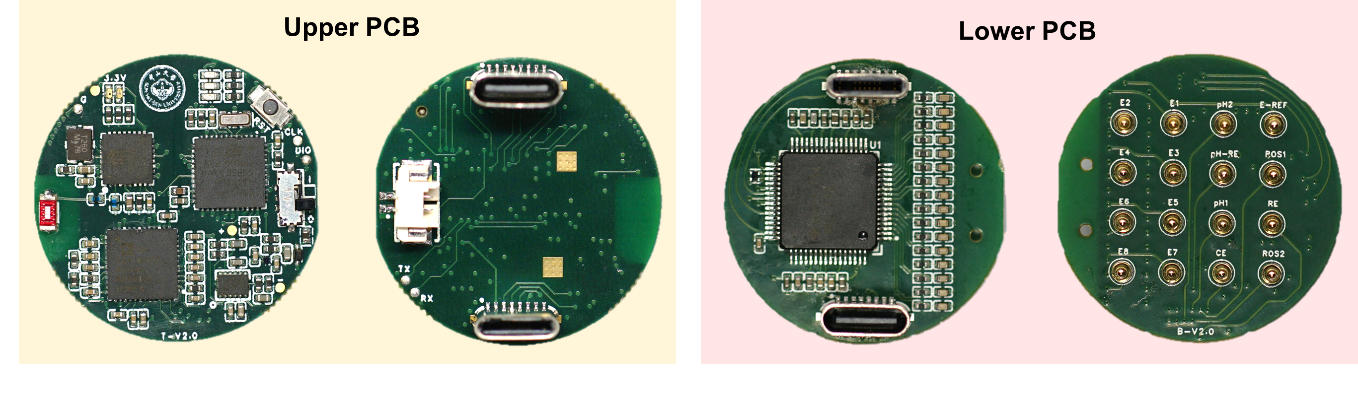
**Figure S31.** Photos of the front and back of two PCBs.


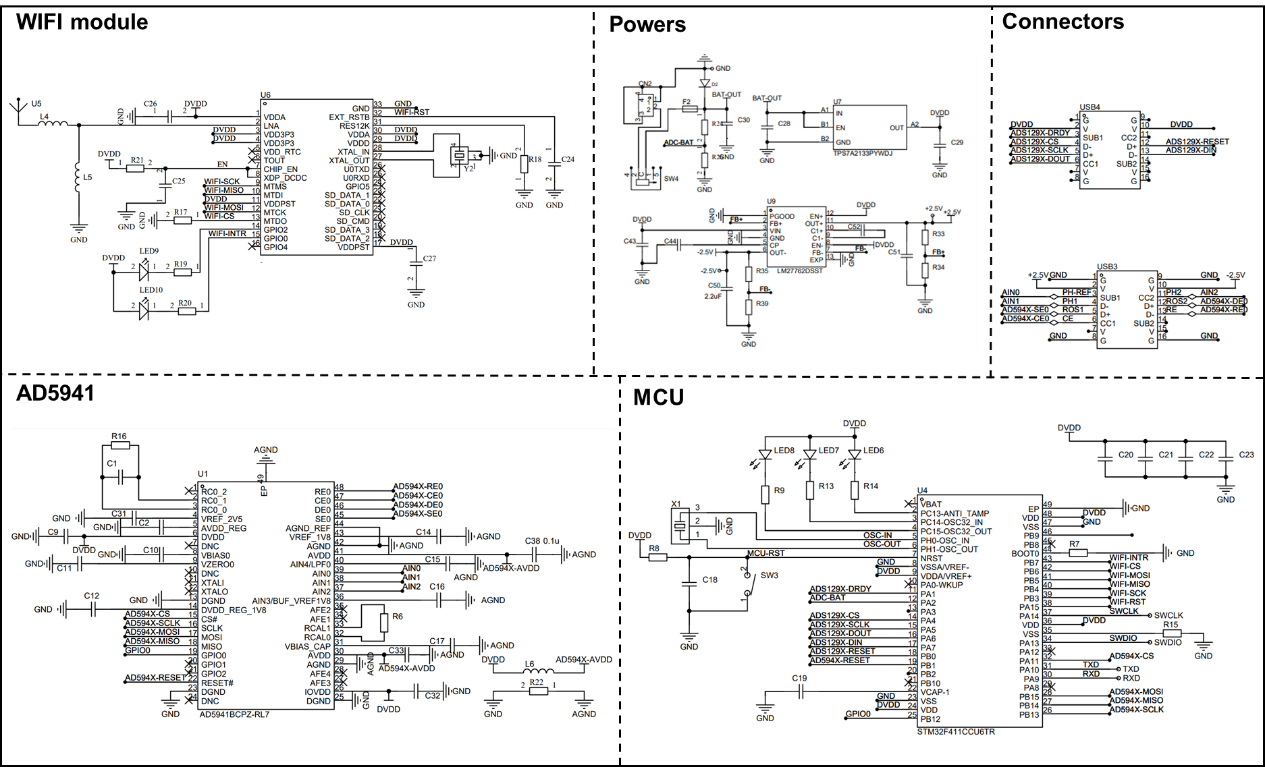


**Figure S32.** Schematic circuit design of the upper PCB. The PCB mainly consists of a power supply module, a Wi-Fi module, a microcontroller unit (MCU) module and an electrochemical analogue front end (AFE) module.


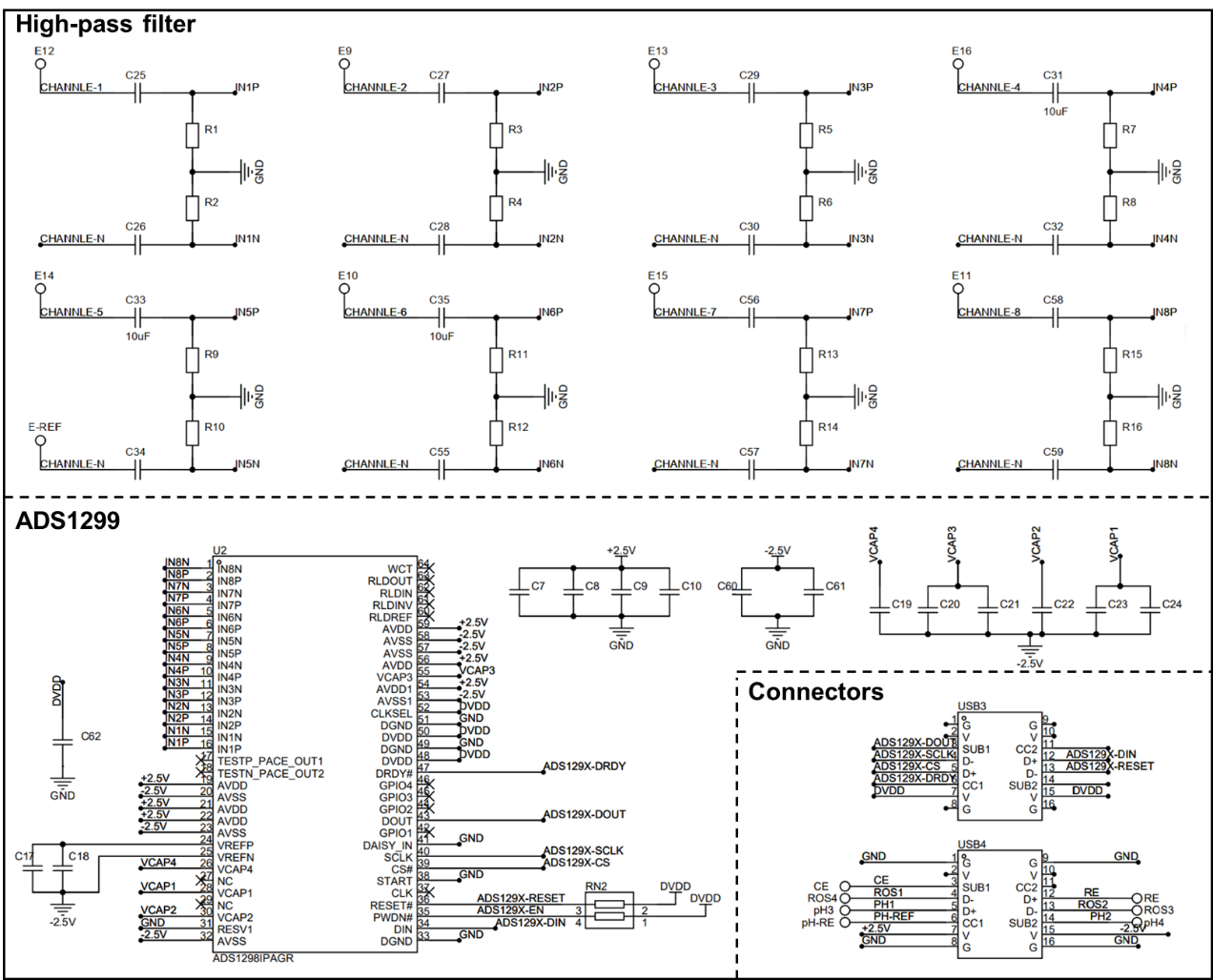


**Figure S33.** Schematic circuit design of the lower PCB. The PCB mainly consists of a first-order high-pass filter circuit, and an EMG analogue front-end (AFE) module.


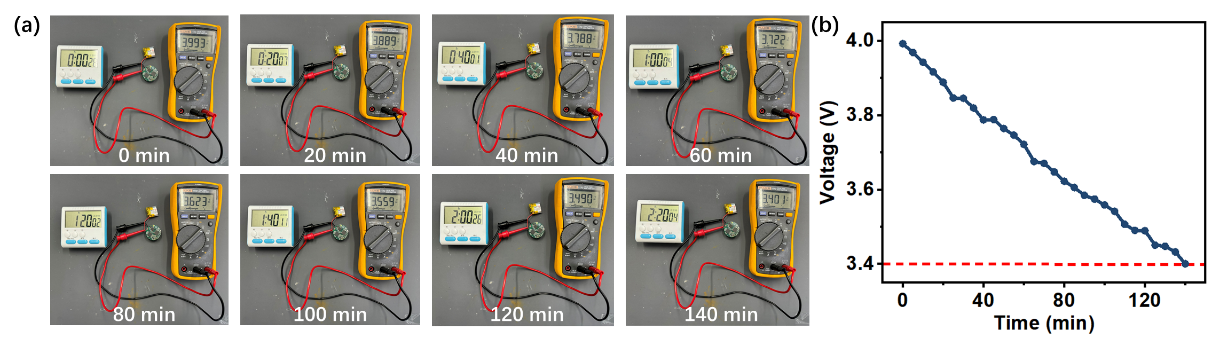


**Figure S34.** The endurance of the detection device in standby mode. (a) Battery voltage over time for detection device in standby mode. (b) Battery voltage-time curves. The detection device is powered by converting the lithium battery output to 3.3 V using a LDO. When the voltage of the lithium battery is greater than 3.4 V, the LDO can output 3.3 V normally.


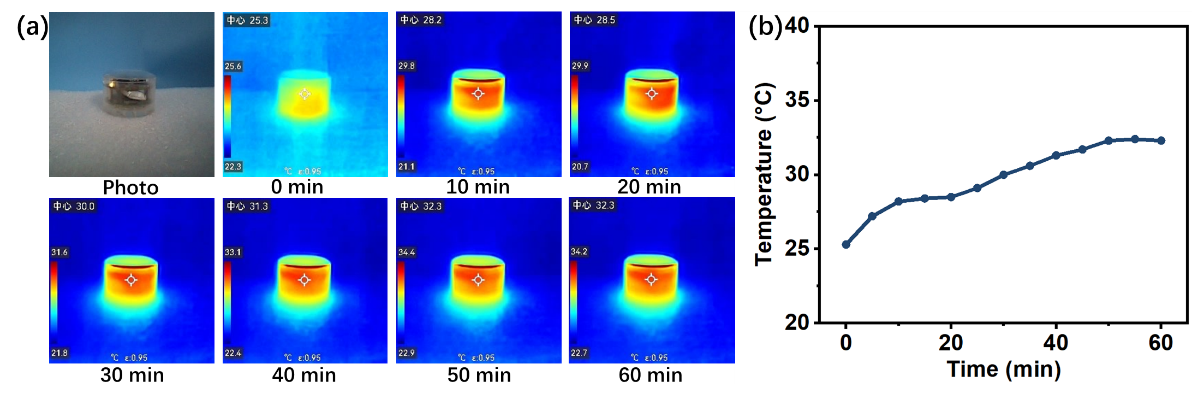


**Figure S35.** Temperature rise of the device during operation. (a) Thermal image of the device. (b)Temperature plot of the hot spot of the device.


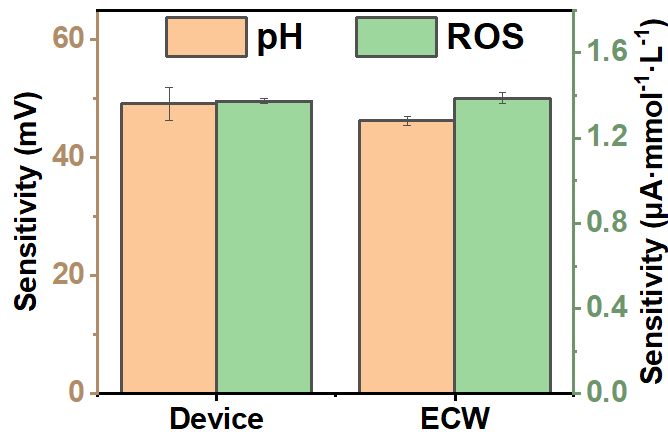


**Figure S36.** Compare the results of pH and ROS detection using the device and a commercial electrochemical workstation (ECW). For pH sensing, the detection sensitivity of the prepared device was 49.15 mV, and the detection sensitivity for ECW was 46.26 mV. For ROS sensing, the detection sensitivity of the prepared device was 1.37 μA/mM, and the detection sensitivity for ECW was 1.39 μA/mM.

**Section 8.** **Animal studies**

**
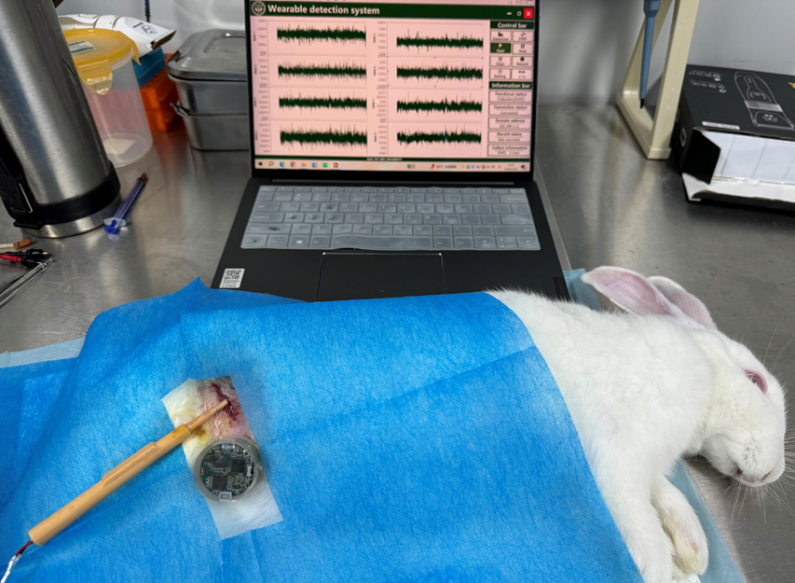
**

**Figure S37.** Schematic diagram of the in vivo experimental setup. Healthy New Zealand rabbits were selected as experimental subjects. Before the experiment, the rabbit needs to be anaesthetised and immobilised. The sciatic nerve was surgically exposed at the hind limb site of the rabbit. The exposed sciatic nerve was clamped using double hooked electrodes. Appropriate current stimulation was applied to the sciatic nerve through the double hook electrodes with the aim of inducing an action potential in the sciatic nerve, which in turn was transmitted to the muscle and generated CMAP. The RMNEA-integrated system was placed on the leg muscle to record physiological signals from the leg muscle tissue, including CMAP, ROS and pH.


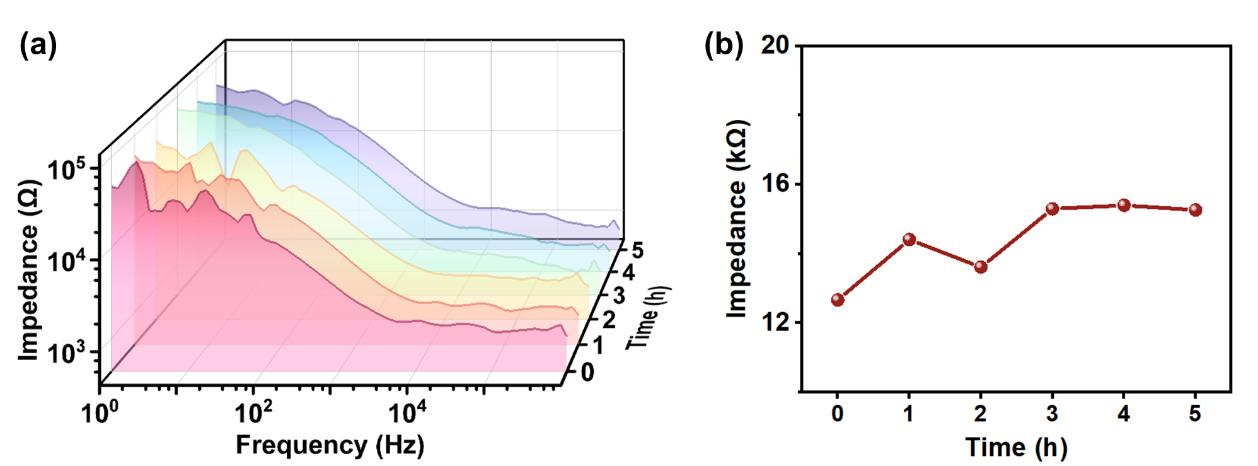


**Figure S38.** Changes in TSMN electrode-rabbit skin impedance. (a) Long-term monitoring of electrode-rabbit skin impedance using TSMN for 6 hours. (b) Change in TSMN electrode-rabbit skin impedance at 100 Hz for 6 hours.


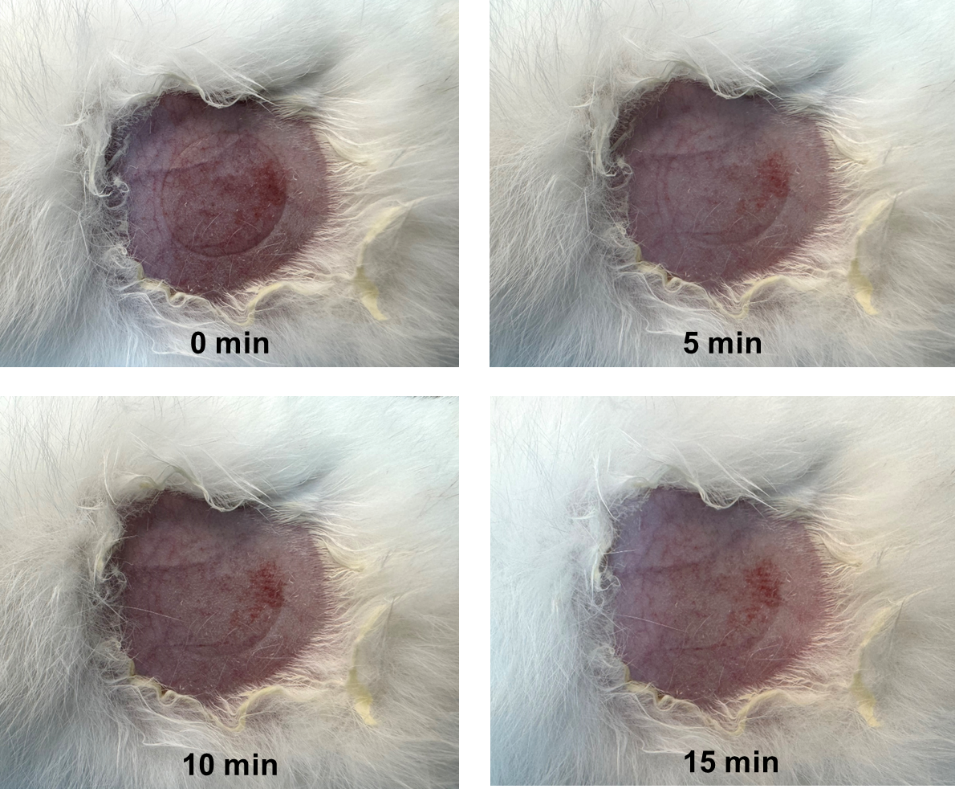


**Figure S39.** The rabbit skin was pressed once by the RMNEA and the skin began to recover after removal of the RMNEA-integrated system. Surface morphology of the punctured skin was recorded every 5 min.

**Note S6.** Calibration of Electrochemical Sensing in Muscle Fatigue Model

For the detection of ROS concentration and pH value, the average value of the data collected for two minutes is calculated, and calibration is performed based on the in vitro detection data. Set the ROS concentration $\mathrm{ROS}_{0h}$or pH value $\mathrm{pH}_{0h}$at 0 hours as the reference value measured in blood. In the subsequent data collection, calculation formulas are as follows

$\mathrm{ROS}_{1h}$ = $\frac{y_{1h}-y_{0h}}{k_{ROS}}+\mathrm{ROS}_{0h}$, $\mathrm{pH}_{1h}$ = $\frac{x_{1h}-x_{0h}}{k_{pH}}+\mathrm{pH}_{0h}$

$\mathrm{ROS}_{2h}$ = $\frac{y_{2h}-y_{0h}}{k_{ROS}}+\mathrm{ROS}_{0h}$, $\mathrm{pH}_{2h}$ = $\frac{x_{2h}-x_{0h}}{k_{pH}}+\mathrm{pH}_{0h}$

$\mathrm{ROS}_{3h}$ = $\frac{y_{3h}-y_{0h}}{k_{ROS}}+\mathrm{ROS}_{0h}$, $\mathrm{pH}_{3h}$ = $\frac{x_{3h}-x_{0h}}{k_{pH}}+\mathrm{pH}_{0h}$

$\mathrm{ROS}_{4h}$ = $\frac{y_{4h}-y_{0h}}{k_{ROS}}+\mathrm{ROS}_{0h}$, $\mathrm{pH}_{4h}$ = $\frac{x_{4h}-x_{0h}}{k_{pH}}+\mathrm{pH}_{0h}$

$\mathrm{ROS}_{5h}$ = $\frac{y_{5h}-y_{0h}}{k_{ROS}}+\mathrm{ROS}_{0h}$, $\mathrm{pH}_{5h}$ = $\frac{x_{5h}-x_{0h}}{k_{pH}}+\mathrm{pH}_{0h}$

Where y represents the current value detected by the detection device for ROS, and x represents the voltage value detected by the detection device for pH. $k_{ROS}$ and $k_{pH}$ are the slopes of the curves obtained from in vitro testing.

**Note S7.** Calibration of Electrochemical Sensing in SNI Model

For the detection of ROS concentration and pH value, the average value of the data collected for two minutes is calculated, and calibration is performed based on the in vitro detection data. Set the ROS concentration $\mathrm{ROS}_{con}$or pH value $\mathrm{pH}_{con}$of the control group to the reference value measured in blood. In the subsequent data collection, calculation formulas are as follows

$\mathrm{ROS}_{SNI 1d}$ = $\frac{y_{SNI 1d}-y_{con}}{k_{ROS}}+\mathrm{ROS}_{con}$, $\mathrm{pH}_{SNI 1d}$ = $\frac{x_{SNI 1d}-x_{con}}{k_{pH}}+\mathrm{pH}_{con}$

$\mathrm{ROS}_{SNI 2d}$ = $\frac{y_{SNI 2d}-y_{con}}{k_{ROS}}+\mathrm{ROS}_{con}$, $\mathrm{pH}_{SNI 2d}$ = $\frac{x_{SNI 2d}-x_{con}}{k_{pH}}+\mathrm{pH}_{con}$

Where y represents the current value detected by the detection device for ROS, and x represents the voltage value detected by the detection device for pH.$k_{ROS}$ and $k_{pH}$ are the slopes of the curves obtained from in vitro testing.

Multiple punctures were made into the dorsal skin of New Zealand rabbits using the RMNEA, and areas of dorsal skin with punctures were labelled. At 24 hours after the puncture, the dorsal skin area with punctures was dissected to ensure that the sampling area completely covered the puncture point and its surrounding skin tissue. At the same time, a sample of the same size was also taken from the back skin area without puncture as a control. The fixed skin samples were subjected to the steps of dehydration, transparency, wax dipping and embedding to make paraffin blocks. The paraffin blocks were then cut into thin slices using a slicer and stained with hematoxylin and eosin (H&E). It was observed that there was no significant increase in inflammatory cells in the skin with punctures, suggesting that the inflammatory response induced by the RMNEA was relatively mild.


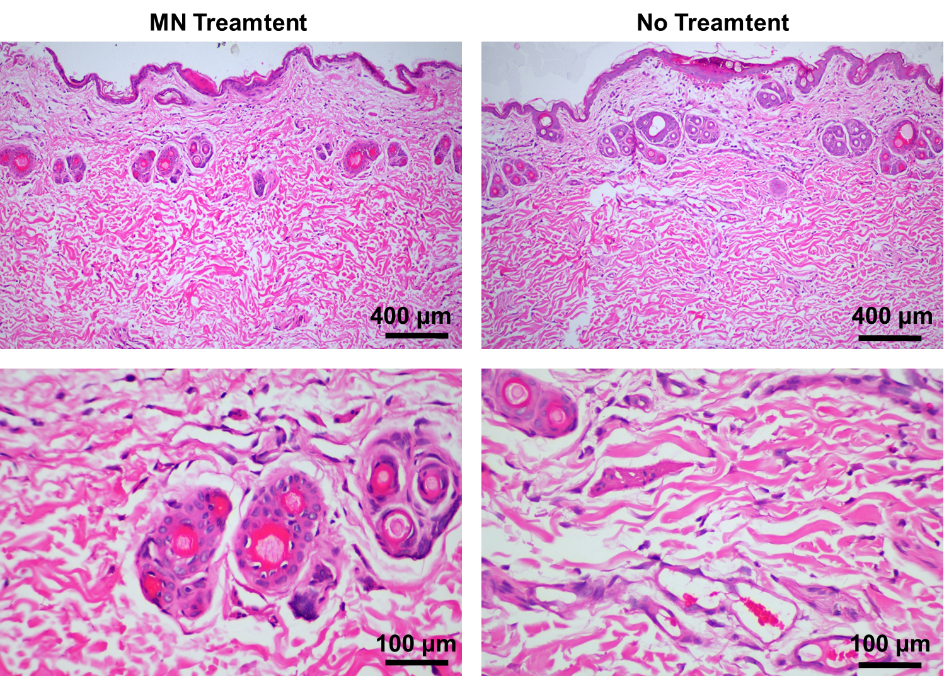


**Figure S40.** Enlarged imaging of skin slices with H&E staining.

At the end of the experiment, New Zealand rabbits were euthanised by injection of an overdose of anaesthetic. An incision was made along the original surgical path to remove the sciatic nerve tissue at the site of injury, which was approximately 1 cm in length, and these nerve samples were immersed in 4% paraformaldehyde for fixation. The nerve samples were processed through various levels of alcohol dehydration, followed by clearing, paraffin impregnation and embedding to make longitudinal sections of approximately 5 μm thickness. The sections were treated with xylene dewaxing and all levels of ethanol dehydration before HE staining (hematoxylin-eosin staining). At the same time, normal sciatic nerve tissue was also removed for the same processing procedure. Significant pathological changes exhibited by the sciatic nerve at the site of injury were observed, including severe impairment of axonal axon function, abnormal proliferation and disorganised arrangement of nerve fibre tissues, collagenisation of the local area, and inflammatory reaction of the tissues surrounding the nerve transverse muscle, suggesting the presence of damage to the sciatic nerve.


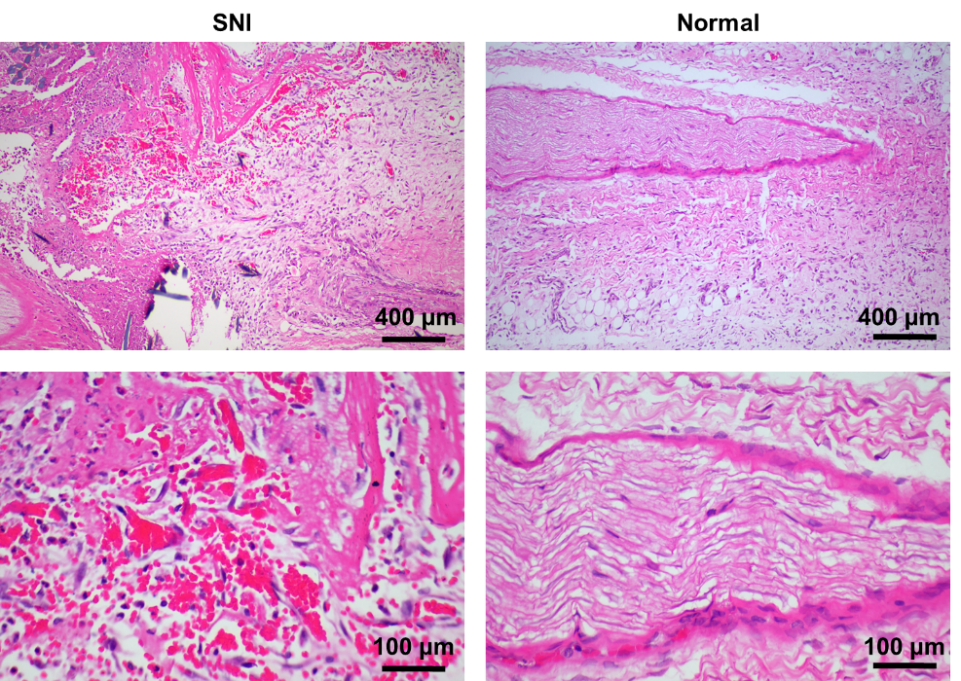


**Figure S41.** Enlarged imaging of sciatic nerve tissue slices with H&E staining.

References:

[1] H. Kim, J. Lee, U. Heo, D. K. Jayashankar, K.-C. Agno, Y. Kim, C. Y. Kim, Y. Oh, S.-H. Byun, B. Choi, *Science Advances* **2024**, 10, eadk5260.

[2] H. Ji, M. Wang, Y. Wang, Z. Wang, Y. Ma, L. Liu, H. Zhou, Z. Xu, X. Wang, Y. Chen, *npj Flexible Electronics* **2023**, 7, 46.

[3] Y. Kudo, M. Arai, N. Miki, *Micro & Nano Letters* **2017**, 12, 545.

[4] J. Li, Y. Ma, D. Huang, Z. Wang, Z. Zhang, Y. Ren, M. Hong, Y. Chen, T. Li, X. Shi, *Nano-Micro Letters* **2022**, 14, 132.

[5] D. Byun, S. J. Cho, S. Kim, *Journal of Micromechanics and Microengineering* **2013**, 23, 125010.

[6] L. Ren, S. Xu, J. Gao, Z. Lin, Z. Chen, B. Liu, L. Liang, L. Jiang, *Sensors* **2018**, 18, 1191.

[7] M. Arai, Y. Nishinaka, N. Miki, *Japanese Journal of Applied Physics* **2015**, 54, 06FP14.

[8] H. Zhang, W. Pei, Y. Chen, X. Guo, X. Wu, X. Yang, H. Chen, *IEEE Transactions on Biomedical Engineering* **2015**, 63, 1136.

[9] Q. Zhao, E. Gribkova, Y. Shen, J. Cui, N. Naughton, L. Liu, J. Seo, B. Tong, M. Gazzola, R. Gillette, *Science Advances* **2024**, 10, eadn7202.

[10] M. Mahmood, S. Kwon, H. Kim, Y. S. Kim, P. Siriaraya, J. Choi, B. Otkhmezuri, K. Kang, K. J. Yu, Y. C. Jang, *Advanced Science* **2021**, 8, 2101129.

[11] R. Wang, X. Jiang, W. Wang, Z. Li, *Sensors and Actuators B: Chemical* **2017**, 244, 750.

[12] Y. Hou, Z. Li, Z. Wang, H. Yu, *Microsystems & nanoengineering* **2021**, 7, 53.

[13] K. J. Krieger, J. Liegey, E. M. Cahill, N. Bertollo, M. M. Lowery, E. D. O'Cearbhaill, *Advanced Materials Technologies* **2020**, 5, 2000518.

[14] J. J. García-Guzmán, C. Perez-Rafols, M. Cuartero, G. A. Crespo, *ACS sensors* **2021**, 6, 1129.

[15] X. Huang, B. Liang, S. Zheng, F. Wu, M. He, S. Huang, J. Yang, Q. Ouyang, F. Liu, J. Liu, *Bio-Design and Manufacturing* **2024**, 7, 14.

[16] W. Lee, S.-h. Jeong, Y.-W. Lim, H. Lee, J. Kang, H. Lee, I. Lee, H.-S. Han, S. Kobayashi, M. Tanaka, *Science Advances* **2021**, 7, eabi6290.

[17] G. K. Mani, K. Miyakoda, A. Saito, Y. Yasoda, K. Kajiwara, M. Kimura, K. Tsuchiya, *ACS Applied Materials & Interfaces* **2017**, 9, 21651.

[18] L. Tang, D. Du, F. Yang, Z. Liang, Y. Ning, H. Wang, G.-J. Zhang, *Scientific reports* **2015**, 5, 11627.

[19] M. Parrilla, A. Vanhooydonck, M. Johns, R. Watts, K. De Wael, *Sensors and Actuators B: Chemical* **2023**, 378, 133159.

[20] M. Dervisevic, E. Dervisevic, L. Esser, C. D. Easton, V. J. Cadarso, N. H. Voelcker, *Biosensors and Bioelectronics* **2023**, 222, 114955.

[21] J.-X. Zhou, F. Ding, L.-N. Tang, T. Li, Y.-H. Li, Y.-J. Zhang, H.-Y. Gong, Y.-T. Li, G.-J. Zhang, *Analyst* **2018**, 143, 4469.

[22] K. Wang, Q. Ding, M. Qi, W. Zhang, Y. Hou, R. Cao, C. Li, L. Xu, L. Wang, J. S. Kim, *Advanced Functional Materials* **2024**, 2316820.

[23] Q. Jin, H. J. Chen, X. Li, X. Huang, Q. Wu, G. He, T. Hang, C. Yang, Z. Jiang, E. Li, *Small* **2019**, 15, 1804298.

[24] F. Liu, Z. Lin, Q. Jin, Q. Wu, C. Yang, H.-J. Chen, Z. Cao, D.-a. Lin, L. Zhou, T. Hang, *ACS applied materials & interfaces* **2019**, 11, 4809.

[25] C. Zuliani, F. S. Ng, A. Alenda, A. Eftekhar, N. S. Peters, C. Toumazou, *Analyst* **2016**, 141, 4659.

[26] J.-X. Zhou, L.-N. Tang, F. Yang, F.-X. Liang, H. Wang, Y.-T. Li, G.-J. Zhang, *Analyst* **2017**, 142, 4322.

[27] C. Hegarty, A. McConville, R. J. McGlynn, D. Mariotti, J. Davis, *Materials Chemistry and Physics* **2019**, 227, 340.

[28] Y. Abe, S. Yamaguchi, H. Abe, R. Takizawa, Y. Tatsui, M. Nishizawa, *Microelectronic Engineering* **2022**, 265, 111877.

[29] J. Zhu, L. Wang, S. Xu, L. Peng, Z. Gao, S. Liu, S. Xi, S. Ma, W. Cai, *Sensors and Actuators B: Chemical* **2024**, 419, 136436.

[30] Y. Yang, C. Sheng, F. Dong, S. Liu, *Biosensors and Bioelectronics* **2024**, 256, 116280.

[31] Z. Liu, X. Xu, S. Huang, X. Huang, Z. Liu, C. Yao, M. He, J. Chen, H.-j. Chen, J. Liu, *Microsystems & Nanoengineering* **2024**, 10, 72.

[32] X. Huang, B. Liang, S. Huang, Z. Liu, C. Yao, J. Yang, S. Zheng, F. Wu, W. Yue, J. Wang, *Theranostics* **2024**, 14, 1662.

[33] P. Tapfumaneyi, M. Imran, Y. Mohammed, M. S. Roberts, *Frontiers in Drug Delivery* **2022**, 2, 957732.

[34] S. Tayyaba, M. W. Ashraf, N. Afzulpurkar, M. Khaleeq ur Rahman, presented at ASME International Mechanical Engineering Congress and Exposition **2013**.

[35] Z. Chen, Y. Lin, W. Lee, L. Ren, B. Liu, L. Liang, Z. Wang, L. Jiang, *ACS applied materials & interfaces* **2018**, 10, 29338.

[36] W. Shu, H. Heimark, N. Bertollo, D. J. Tobin, E. D. O'Cearbhaill, A. N. Annaidh, *Acta Biomaterialia* **2021**, 135, 403.

[37] C. Li, G. Guan, R. Reif, Z. Huang, R. K. Wang, *Journal of The Royal Society Interface* **2012**, 9, 831.

[38] M. Vafaiee, F. A. Mahyari, A. Kalantarian, M. Janahmadi, R. Mohammadpour, P. Sasanpour, *Biosensors and Bioelectronics: X* **2024**, 18, 100485.

[39] M. E. E. Alahi, Y. Liu, S. Khademi, A. Nag, H. Wang, T. Wu, S. C. Mukhopadhyay, *Biosensors* **2022**, 12, 1044.

[40] N. A. Alba, Z. J. Du, K. A. Catt, T. D. Kozai, X. T. Cui, *Biosensors* **2015**, 5, 618.

[41] Y. Chen, W. Pei, S. Chen, X. Wu, S. Zhao, H. Wang, H. Chen, *Sensors and Actuators B: Chemical* **2013**, 188, 747.

[42] E. He, S. Xu, Y. Dai, Y. Wang, G. Xiao, J. Xie, S. Xu, P. Fan, F. Mo, M. Wang, *ACS sensors* **2021**, 6, 3377.

[43] S. H. Ko, S. W. Kim, Y. J. Lee, *Scientific reports* **2021**, 11, 21101.

[44] S. H. Ko, S. W. Kim, S. H. Lee, Y. J. Lee, *Scientific Reports* **2023**, 13, 20274.

[45] C. Boehler, T. Stieglitz, M. Asplund, *Biomaterials* **2015**, 67, 346.

[46] B. Körbitzer, P. Krauß, S. Belle, J. J. Schneider, C. Thielemann, *ChemNanoMat* **2019**, 5, 427.

[47] Q. Zeng, S. Yu, Z. Fan, Y. Huang, B. Song, T. Zhou, *Nanomaterials* **2022**, 12, 3445.

[48] C. Chen, S. Ruan, X. Bai, C. Lin, C. Xie, I.-S. Lee, *Materials Science and Engineering: C* **2019**, 103, 109865.

[49] S. Liu, Y. Wang, Y. Zhao, L. Liu, S. Sun, S. Zhang, H. Liu, S. Liu, Y. Li, F. Yang, *Advanced Materials* **2024**, 36, 2304297.

[50] B. Fan, A. V. Rodriguez, D. G. Vercosa, C. Kemere, J. T. Robinson, *Journal of neural engineering* **2020**, 17, 036029.

[51] N. B. Babaroud, S. J. Rice, M. C. Perez, W. A. Serdijn, S. Vollebregt, V. Giagka, *Nanoscale* **2024**, 16, 3549.

[52] N. Chen, B. Luo, A. C. Patil, J. Wang, G. G. L. Gammad, Z. Yi, X. Liu, S.-C. Yen, S. Ramakrishna, N. V. Thakor, *ACS nano* **2020**, 14, 8059.

[53] W. Yi, C. Chen, Z. Feng, Y. Xu, C. Zhou, N. Masurkar, J. Cavanaugh, M. M.-C. Cheng, *Nanotechnology* **2015**, 26, 125301.

[54] X. Du, L. Yang, N. Liu, *Small Science* **2023**, 3, 2300008.

[55] S. Imani, A. J. Bandodkar, A. M. V. Mohan, R. Kumar, S. Yu, J. Wang, P. P. Mercier, *Nature Communications* **2016**, 7, 11650.

[56] T. Li, B. Liang, Z. Ye, L. Zhang, S. Xu, T. Tu, Y. Zhang, Y. Cai, B. Zhang, L. Fang, X. Mao, S. Zhang, G. Wu, Q. Yang, C. Zhou, X. Cai, X. Ye, *Biosensors and Bioelectronics* **2022**, 198, 113855.

[57] B. Gil, S. Anastasova, G. Z. Yang, *Sensors* **2019**, 19, 1616.

[58] H. Lee, S. Lee, J. Kim, H. Jung, K. J. Yoon, S. Gandla, H. Park, S. Kim, *npj Flexible Electronics* **2023**, 7, 20.

[59] S. Benatti, F. Casamassima, B. Milosevic, E. Farella, P. Schönle, S. Fateh, T. Burger, Q. Huang, L. Benini, *IEEE Transactions on Biomedical Circuits and Systems* **2015**, 9, 620.

[60] X. Liu, J. Sacks, M. Zhang, A. G. Richardson, T. H. Lucas, J. Van der Spiegel, *IEEE Transactions on Circuits and Systems II: Express Briefs* **2016**, 64, 1257.

[61] S. Benatti, F. Montagna, V. Kartsch, A. Rahimi, D. Rossi, L. Benini, *IEEE Transactions on Biomedical Circuits and Systems* **2019**, 13, 516.

[62] X. Huang, B. Liang, S. Zheng, F. Wu, M. He, S. Huang, J. Yang, Q. Ouyang, F. Liu, J. Liu, H.-j. Chen, X. Xie, *Bio-Design and Manufacturing* **2024**, 7, 14.

[63] S. Huang, C. Yao, M. He, X. Huang, Z. Liu, J. Chen, L. Jiang, H.-j. Chen, X. Xie, *Advanced Engineering Materials* **2024**, 26, 2301662.

[64] Z. Liu, J. Liu, T. Sun, D. Zeng, C. Yang, H. Wang, C. Yang, J. Guo, Q. Wu, H.-J. Chen, X. Xie, *ACS Sensors* **2021**, 6, 3112.
